# Synthesis and binding studies of two new macrocyclic receptors for the stereoselective recognition of dipeptides

**DOI:** 10.3762/bjoc.6.5

**Published:** 2010-01-19

**Authors:** Ana Maria Castilla, M Morgan Conn, Pablo Ballester

**Affiliations:** 1 Institute of Chemical Research of Catalonia (ICIQ), Avgda. Països Catalans 16, 43007 Tarragona, Spain; 2 Amherst College, Amherst, MA 01002, USA; 3 now at PTC Therapeutics, Inc., 100 Corporate Court, South Plainfield, NJ 07080, USA; 4 Catalan Institution for Research and Advanced Studies (ICREA), Passeig Lluís Companys, 23, 08018 Barcelona, Spain

**Keywords:** dipeptides, host–guest, macrocyclic, molecular recognition, receptors, stereoselective

## Abstract

We present here the design, synthesis, and analysis of a series of receptors for peptide ligands inspired by the hydrogen-bonding pattern of protein β-sheets. The receptors themselves can be regarded as strands 1 and 3 of a three-stranded β-sheet, with cross-linking between the chains through the 4-position of adjacent phenylalanine residues. We also report on the conformational equilibria of these receptors in solution as well as on their tendency to dimerize. ^1^H NMR titration experiments are used to quantify the dimerization constants, as well as the association constant values of the 1:1 complexes formed between the receptors and a series of diamides and dipeptides. The receptors show moderate levels of selectivity in the molecular recognition of the hydrogen-bonding pattern present in the diamide series, selecting the α-amino acid-related hydrogen-bonding functionality. Only one of the two cyclic receptors shows modest signs of enantioselectivity and moderate diastereoselectivity in the recognition of the enantiomers and diastereoisomers of the Ala-Ala dipeptide (ΔΔ*G*^0^**_1_** (DD-DL) = −1.08 kcal/mol and ΔΔ*G*^0^**_1_** (DD-LD) = −0.89 kcal/mol). Surprisingly, the linear synthetic precursors show higher levels of stereoselectivity than their cyclic counterparts.

## Introduction

Manipulation of protein–protein interactions is gaining interest as they are known to play a critical role in important biological processes such as the normal function of cellular/organelle structure, immune response, enzyme inhibitors, signal transduction, and apoptosis. Rational protein surface recognition poses a challenging test to our actual knowledge of molecular design. Nevertheless, its practice and developments will provide a better understanding of protein–protein interactions. Interestingly, in molecule-based disease therapy, the disruption of protein–protein interactions by small molecules constitutes an alternative approach to the classical active-site enzyme inhibition design. One of the strategies employed for binding protein surfaces relies on the use of arrays of synthetic receptors originally designed for the recognition of oligopeptides. Consequently, the selective recognition of oligopeptides represents an intermediate step toward the recognition of protein surfaces [1]. The studies of host–guest complexes as model systems of peptide–peptide interactions are of particular interest because they may provide insight into the structural basis of the high size/shape specificities and enantioselectivities exhibited by the complex protein–protein recognition processes that occur in biology. Moreover, short oligopeptides are themselves worthwhile targets for recognition and their conformational flexibility represents an added challenge to achieve selective binding. The preparation of synthetic receptors for the selective binding of short oligopeptides has potential applications in the development of diagnostic sensors, separation techniques, and therapeutic agents.

With respect to this latter point, there is a significant interest in the advance of receptors that selectively bind the D-Ala-D-Ala dipeptide, the common target for the vancomycin antibiotics. This group of antibiotics is active against certain aerobic and anaerobic Gram-positive bacteria, and has been used for many years as treatment of last resort in clinical wards [2–4]. However, vancomycin resistance has recently been identified among clinical isolates of several Gram-positive species [5–8]. Therefore, although many examples already exist in the literature [9–10], the design and synthesis of new synthetic receptors for this dipeptide is still a relevant endeavor not only in terms of understanding the interactions that take place during vancomycin action, but also because the structures of the most efficient receptors prepared might be useful as scaffolds for future antibiotics.

Herein, we report the design and synthesis of two new macrocyclic receptors, **1** and **2**, conceived for the binding of dipeptides, in particular for the selective recognition of D-Ala-D-Ala. We also report on the studies performed using these two macrocyclic receptors, as well as their linear precursors, in the molecular recognition of a series of dipeptides and diamides with diverse hydrogen-bonding patterns. We rationalize the observed modulation of their binding affinity as a function of the hydrogen-bonding pattern exhibited by the target molecule. We also describe the levels of stereoselectivity displayed by these receptors in the recognition of the diastereoisomers and enantioisomers of Ala-Ala dipeptide. We explain the differences observed in their binding abilities as a function of conformational rigidity (macrocyclic vs linear receptors).

## Results and Discussion

### Design of the synthetic receptors: general considerations

**Figure 1:**
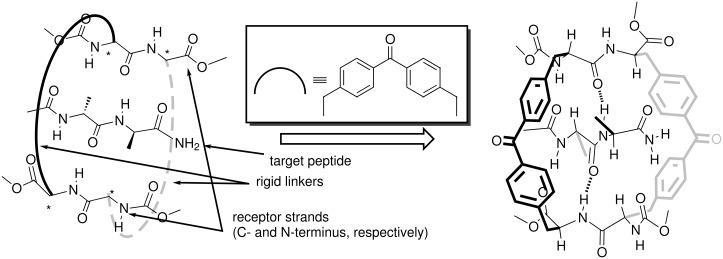
Schematic representation of the design of a host–guest complex based on antiparallel β-sheet geometry. *The presence of a stereogenic center.

The design of the receptors described in this article is based on the interactions that occur in the β-sheets commonly found in the secondary structure of many biologically relevant proteins. We start from a schematic termolecular complex mimicking a three-stranded β-sheet in which the central strand corresponds to the target guest peptide and the two outer strands constitute the structure of the host (Figure 1). In this design, we employ some of the properties of the β-sheet structure – the convergence of hydrogen-bonding patterns and the presence of exposed side chains. It is worth mentioning that the β-sheet structure has already been used as the inspirational binding motif for the preparation of other synthetic receptors for peptides [11–16]. However, we believe that our design includes some novelties. To reduce the conformational flexibility and confer a certain degree of preorganization to this type of receptor, the use of one or two linkers connecting the two peptide strands is mandatory. The principal difference with respect to previous designs of β-sheet-based synthetic receptors is that the connection between the two peptide strands, used as the receptor’s binding sites, emerges from their side chains and not from their C- or N-terminus. In our final design, we propose the introduction of two linkers connecting the two peptide strands affording, a macrocyclic structure. In doing so, we expect that the molecular recognition properties of the designed receptor will also benefit from the *macrocyclic effect* [17–20]. Simple molecular modeling studies [21] revealed that a benzophenone unit would be ideally suited to span the gap between two methyl side chains emerging from alanyl residues of the outer strands in the three-stranded β-sheet complex (Figure 1).

**Figure 2:**
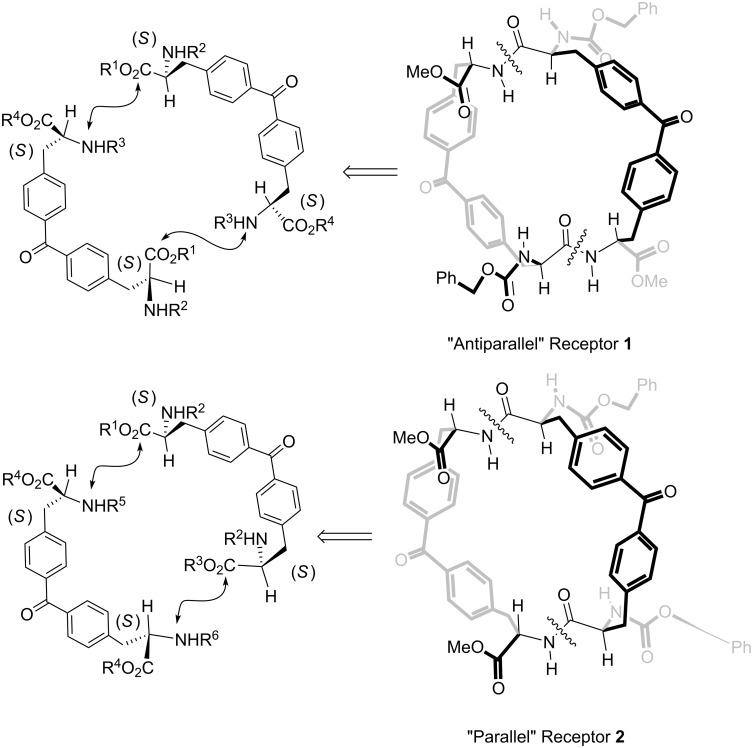
Molecular structures of the two designed receptors **1** and **2** having different relative orientations of the peptide strands.

In proteins, adjacent β-strands can form hydrogen bonds in antiparallel, parallel, or mixed arrangements. In an antiparallel arrangement, the successive β-strands alternate directions so that the N-termini of two adjacent strands are at opposite ends. In a parallel arrangement, all of the N-termini of successive strands are oriented in the same direction [22]. In contrast, successive strands in a mixed-mode arrangement may be parallel or antiparallel to each other. To examine the influence of the relative orientation of the two receptor strands on their binding abilities, we conceived and synthesized two analogous receptors mimicking these two types of arrangements present in the β-sheet structure (Figure 2). The outer peptide strands of receptor **1** are arranged antiparallel to each other (“antiparallel receptor”). That is, the stereogenic center of the C-terminus of one strand is covalently connected to the stereogenic center of the N-terminus of the other strand. Receptor **1** is anticipated to form a mixed-mode sheet structure with an included peptide ligand. Conversely, the two outer strands of receptor **2** are oriented parallel to each other (“parallel receptor”), such that the covalent connections between strands join similar stereogenic centers, C-terminus with C-terminus and N-terminus with N-terminus. Receptor **2** is anticipated to form an antiparallel sheet structure with the included peptide ligand. This change in connectivity does not involve any inversion of the stereogenic centers but only a modification in the sequence of peptide-coupling reactions that yield the cyclic structure, and will be explained below.

**Figure 3:**
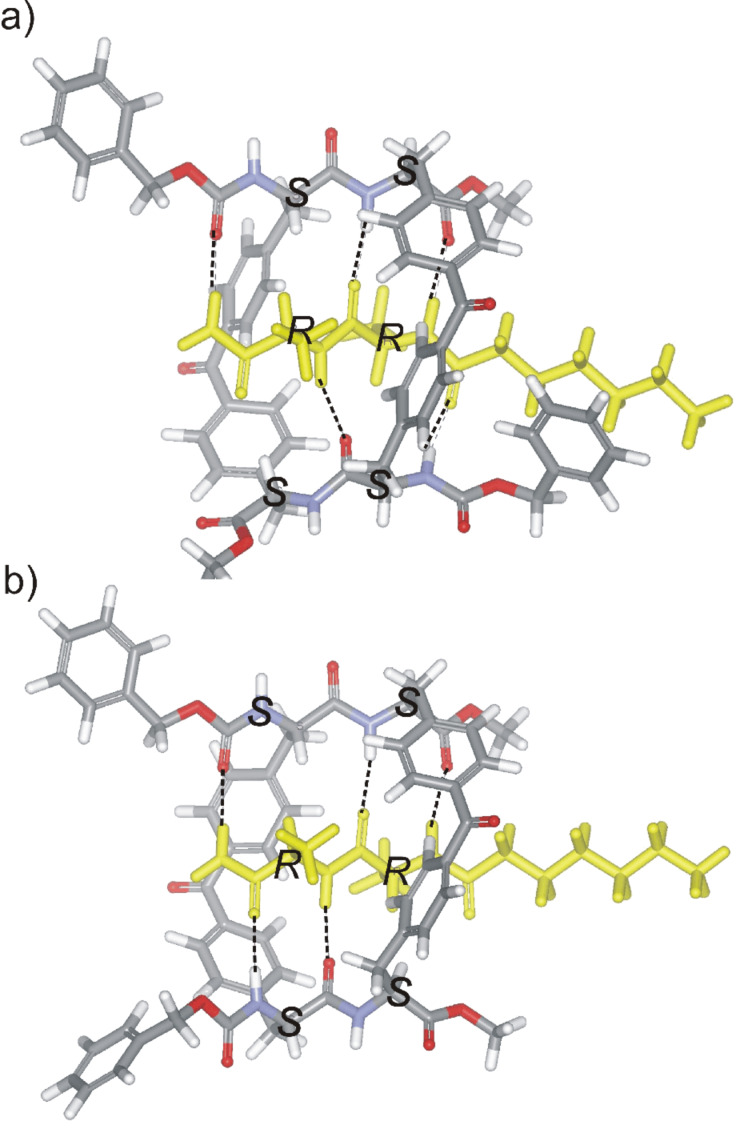
CAChe minimized structures for the “*endo*” complexes formed between receptors **1** (a) and **2** (b) and the *n*-C_6_H_13_CO-D-Ala-D-Ala-NH_2_ dipeptide. The absolute configuration of the stereogenic centers are indicated with a capital letter. Five intermolecular hydrogen bonds are also shown as black dashed lines.

The exploration of the conformational space of both macrocycles, using molecular modeling, indicated the existence of a built-in cavity. These studies also suggested that the reduced conformational flexibility of the receptors avoids the complete collapse of the cavity through the formation of intramolecular hydrogen bonds. Moreover, we were able to minimize structures for the complexes formed between both receptors and *n*-C_6_H_13_CO-D-Ala-D-Ala-NH_2_ in which the dipeptide is threaded through the macrocycle (Figure 3). In these minimized structures, the hydrogen-bonding groups of the receptor converge toward the center of the macrocycle. The macrocycle is large enough to accommodate the threading dipeptide without incurring any substantial steric clashes. We also observed appropriate complementarity between the hydrogen-bonding groups of substrate and receptor (Figure 3). The analysis of the structures of the minimized complexes revealed that they are stabilized by the formation of the same number of hydrogen bonds, that is, five. The hypothesized “*endo*” structure for the complexes of **1** and **2** with *n*-C_6_H_13_CO-D-Ala-D-Ala-NH_2_, in which the ligands thread through the receptor’s macrocycle, also allows for the possibility of binding short amino acid sequences not necessarily located on the edges of larger peptides.

Other considerations, apart from preventing intramolecular hydrogen bond formation, related to the use of bis(alanyl)benzophenone rigid linkers include: a) to avoid steric clashes between the methyl groups of the target peptide and the benzophenone linking chains, the stereogenic centers in the linkers must have the (*S*) configuration, opposite to that of the bound peptide (*R*); b) the benzophenone aromatic ring will also provide a hydrophobic pocket for the neighboring side chains of the target peptide, and may promote the formation of additional CH–π and π–π intermolecular interactions. The pleat of the bound D-Ala-D-Ala peptide is inverted because of the unnatural stereochemistry and the resulting complex is not exactly a β-sheet. Attaching the linking groups in the side chains leaves the ends of the receptor strands open, allowing the introduction of additional interactions between the receptor and the chain of a larger peptide.

As schematically depicted in Figure 2, we planned that both receptors could be obtained through cyclic dimerization, through the formation of two peptide bonds, of two *S*,*S*-bis(alanyl)benzophenone units **3**. In turn, the synthesis of the protected *S*,*S*-bis(alanyl)benzophenone units **3** could be easily achieved by means of Stille carbonylative cross-coupling reactions of two adequately bisprotected *S*-phenylalanine derivatives, iodo-aryl **4** and trimethylstannyl-aryl **5**, following experimental procedures described in recent literature reports [23].

## Synthesis

**Scheme 1:**
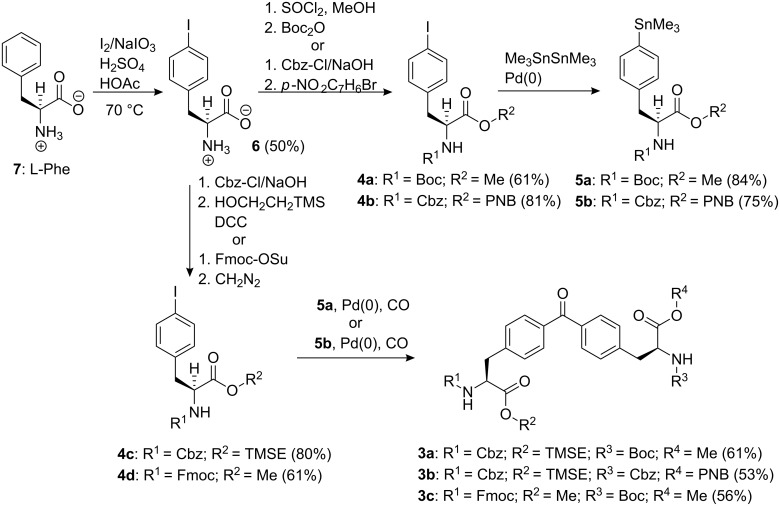
Synthesis of tetraprotected bis(alanyl)benzophenones **3** from L-phenylalanine **7**.

The synthetic strategy designed for the construction of receptors **1** and **2** involves the use of a carbonylative cross-coupling reaction between two aryl derivatives (iodo-aryl **4** and trimethylstannyl-aryl **5**) to prepare 4,4’-bis(alanyl)benzophenones **3**, followed by macrocyclization of two molecular units of **3**. The macrocyclization reaction of two 4,4’-bis(alanyl)benzophenones **3** will be promoted by the sequential and regioselective formation of two peptide bonds between them, the first one through an intermolecular reaction and the second one intramolecularly, affording the desired macrocyclic structures **1** and **2**. The main dissimilarity between the two synthetic strategies resides in the type of functional groups that each 4,4’-bis(alanyl)benzophenone **3** supplies to the macrocyclization reaction. Thus, for the synthesis of antiparallel receptor **1** each benzophenone unit will provide, in an alternative way, one carboxylic and one amino function to the final macrocyclic skeleton. Conversely, for the synthesis of antiparallel receptor **2**, one benzophenone unit will donate its two carboxylic acid functions while the other will participate with its two amino groups. To achieve the regioselective control demanded in the macrocyclization reactions, a precise selection of the orthogonal protecting groups to be included in the bis-amino acid functionalities of the benzophenone derivatives **3** is needed. The starting material for both synthetic routes is 4-iodo-L-phenylalanine (**6**). We prepared **6** in multigram scale starting from commercial L-phenylalanine (**7**) by following a described procedure [24] consisting in the iodination of **7** in acetic acid solution in the presence of I_2_, NaIO_3_, and sulfuric acid (Scheme 1). We obtained (*S*)-**6** in enantiomerically pure form in 50% yield. Since we plan to assemble the 4,4’-bis(alanyl)benzophenones **3** by a Stille carbonylative cross-coupling reaction, the required trimethylstannyl derivatives **5** should be easily prepared from adequately diprotected phenylalanine iodides **4** (Scheme 1).

The mild reaction conditions used in the carbonylative cross-coupling permit the use of common protecting groups of peptide synthesis [24]. This characteristic of the carbonylative cross-coupling reaction allowed us to achieve the differential protection of the two amino acid moieties present in the 4,4’-bis(alanyl)benzophenones **3** by protecting separately the functional groups in the reaction partners, **4** and **5**, before attempting the cross-coupling. We prepared a single orthogonally protected benzophenone **3a** for the synthesis of the antiparallel macrocycle **1**. In contrast, the synthesis of parallel receptor **2** called for the preparation of two differently protected bis(alanyl)benzophenone units, **3b** and **3c**. All iodo-phenyl derivatives **4** were prepared in high yields using standard procedures (Scheme 1). Thus, 4-iodo-L-phenylalanine **6** (I-Phe) was converted into the methyl ester hydrochloride by treatment with thionyl chloride in methanol followed by acylation of the amino group with *tert*-butyl dicarbonate to yield Boc-I-Phe-OMe, **4a**. Iodo-L-phenylalanine **6** was acylated under Schotten–Baumann conditions with benzyl chloroformate to obtain the N-protected amino acid Cbz-I-Phe. This compound was esterified with 2-(trimethylsilyl)ethanol using DCC as coupling agent, providing Cbz-I-Phe-TMSE, **4c**. In a different reaction, Cbz-I-Phe was treated with 4-nitrobenzyl bromide and triethylamine to afford Cbz-I-Phe-PNB, **4b** [25]. Finally, **6** was treated with Fmoc hydroxysuccinimide (Fmoc-OSu) [26–29] to obtain the Fmoc N-protected amino acid that was subsequently esterified with diazomethane affording Fmoc-I-Phe-OMe, **4d**.

The diprotected aryl iodides, **4a** and **4b**, were converted uneventfully to the corresponding diprotected aryl trimethylstannane derivatives, **5a** and **5b**, by reaction with hexamethylditin catalyzed by Pd(0) under inert atmosphere. The organo-stannanes **5** showed signs of decomposition with time, and they were freshly prepared just before being used in the cross-coupling reaction.

The 4,4’-bis(alanyl)benzophenones **3** were prepared in an orthogonally protected form by carbonylative coupling between diprotected iodo-aryl derivatives **4** and diprotected aryl trimethylstannanes **5**, using the experimental conditions described by Morera and Ortar for similar substrates [23]. The reactions were performed at 90 °C under atmospheric CO pressure in the presence of PdCl_2_/PPh_3_, proceeding smoothly to give derivatives **3** with isolated yields, after column chromatography, in the range of 53–61%. This complete synthetic sequence is reminiscent of the work of Lei et al. for the preparation of phosphinate bis-amino acids [24]. This convergent route allows the installation of diverse and differentiable functionality in a small molecule like **3**.

**Scheme 2:**
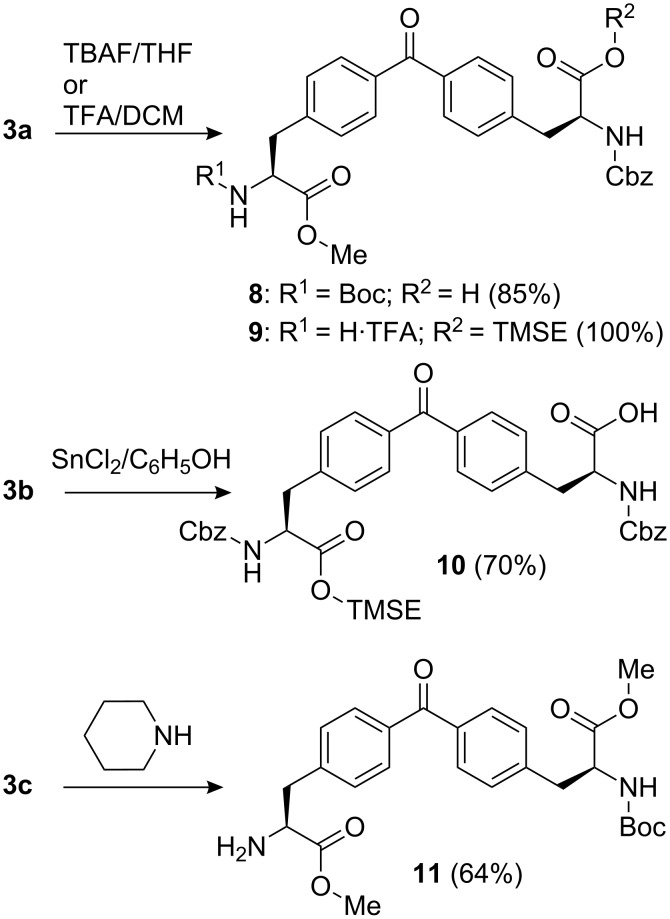
Deprotection reactions of bis(alanyl)benzophenone units **3**.

The sequential peptide coupling of two units of 4,4’-bis(alanyl)benzophenones **3a** should lead to the construction of the designed macrocyclic bis-dipeptide receptor **1**. Benzophenone **3a** was converted into the carboxylic acid **8** by treatment with tetrabutylammonium fluoride [25] (Scheme 2). In a separate reaction, **3a** was treated with trifluoroacetic acid to remove the Boc group and produce the trifluoroacetic salt of amine **9** [25]. Both deprotection reactions proceeded uneventfully in almost quantitative yields. The PNB group in **3b** was removed using a mixture of SnCl_2_ and phenol in acid media and the Fmoc [30] in **3c** using piperidine to obtain **10** and **11**, respectively (Scheme 2).

**Scheme 3:**
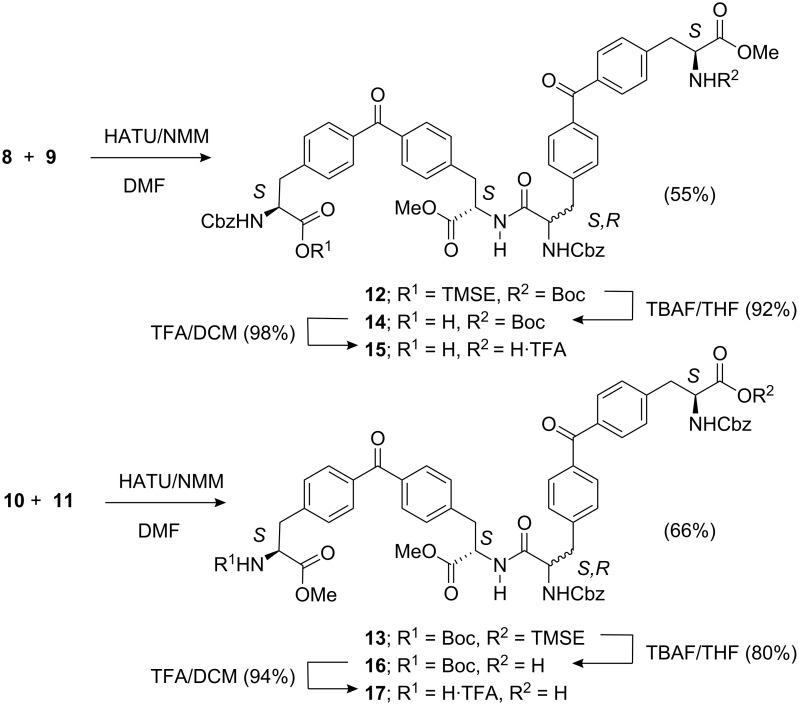
Synthesis of the linear tetrapeptides **15** and **17** as mixtures of diastereoisomers.

Next, we carried out intermolecular peptide-coupling reactions between **8** and **9**, as well as between **10** and **11** to obtain the linear tetrapeptides **12** and **13**, direct precursors of receptors **1** and **2**, respectively. The best results for the coupling reactions were obtained when using a combination of HATU/NMM [31–32] in DMF at room temperature (Scheme 3). The analysis of the crude reaction mixtures by HPLC and ^1^H NMR spectroscopy revealed that both tetrapeptides, **12** and **13**, were obtained as mixtures of two diastereoisomers. Most likely, the stereogenic center in the α-position with respect to the carboxylic group undergoing activation during peptide coupling was partially epimerized. The all-*S* diastereoisomers, (*S*)-**12** and (*S*)-**13**, were the major products detected in the crude reaction mixture. They were isolated as pure compounds using preparative reverse-phase HPLC and fully characterized by a complete set of high-resolution spectra.

However, the subsequent sequence of reactions directed toward the macrocyclic receptors utilized, as starting material, the diastereomeric mixture of **12** or **13** obtained by flash chromatography purification of the reaction crude. The deprotections of the diastereoisomeric mixtures were carried out using standard methods. First, we used fluoride to cleave the TMSE group, and subsequently, we removed the Boc group by the action of TFA. We obtained the bis-deprotected tetrapeptides **15** and **17** in high yield (70–80%).

**Figure 4:**
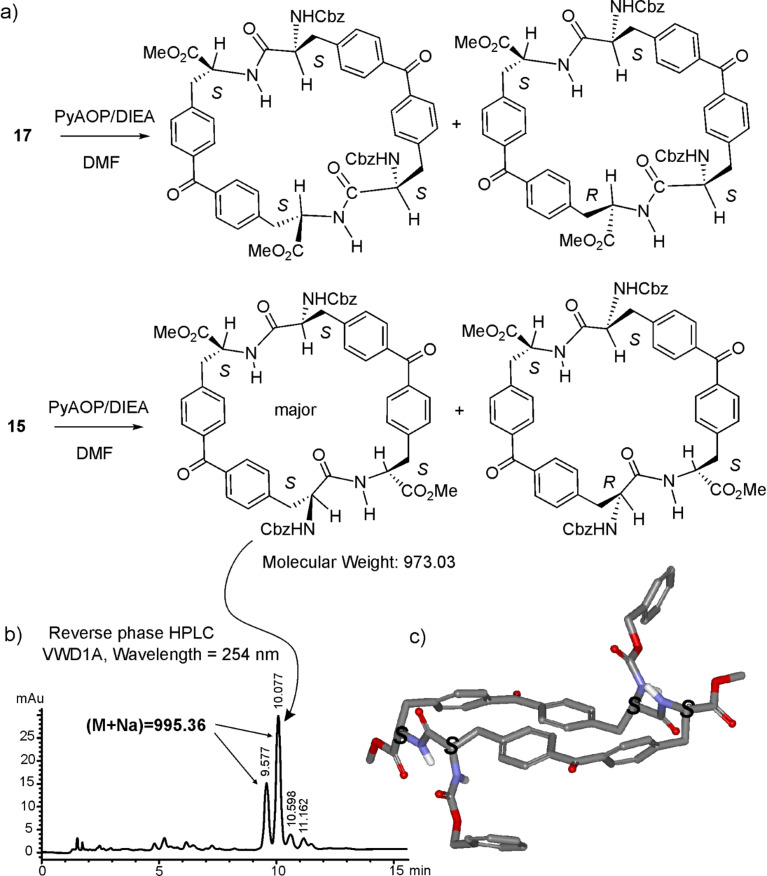
a) Molecular structures of the two major diastereoisomers of the cyclic receptors obtained from the intramolecular coupling reactions of **15** and **17**. b) HPLC chromatogram of the purified fraction containing the mixture of diastereoisomers of **1**. c) X-ray structure of the receptor **1**.

The macrocyclization reactions of the linear tetrapeptides, **15** and **17**, were carried out under high-dilution conditions. Using a syringe pump and under inert atmosphere, a DMF solution of the corresponding linear tetrapeptide was added dropwise, over a period of 12 h, to a stirred DMF solution containing the coupling agent and the base. The purification of the crude macrocyclization reactions using flash chromatography afforded the expected macrocyclic products in acceptable yields but as complex mixtures of diastereoisomers. HPLC–MS analysis of the isolated fraction showed the presence of four different peaks in the chromatogram producing ions with molecular mass value corresponding to the expected cyclic structure. We tentatively assigned the two major peaks to cyclic diastereoisomers formed during the intramolecular peptide-coupling reaction of the all-*S* linear tetrapeptide. As discussed above, one of the two diastereoisomers is probably the outcome of the epimerization reaction experienced by the stereogenic center in the α-position with respect to the carboxylic group undergoing activation. Likewise, the two minor peaks should correspond to cyclic diastereoisomers formed from macrocyclization and concomitant epimerization reactions experienced by the minor linear tetrapeptide *S*,*S*,*S*,*R* also incorporated into the starting material. Figure 4b depicts the HPLC chromatogram obtained from the analysis of the purified fraction containing the mixture of diastereoisomers of receptor **1**. Using normal-phase preparative HPLC, we isolated the two major products of the macrocyclization reaction of **15** as pure compounds. The structures of the isolated products were assigned by means of standard spectroscopic techniques and symmetry considerations to cyclic diastereoisomers of receptor **1**. Furthermore, the structure of the major product of the cyclization of **15** was also characterized in the solid state by X-ray diffraction and proved to be the desired all-*S* antiparallel cyclic receptor **1**. The results obtained in the macrocyclization of tetrapeptide **17** were completely analogous. The all-*S* diastereoisomer corresponds to macrocyclic receptor **2**, and was the major product isolated from the purification of the reaction mixture using normal-phase preparative HPLC. Receptor **2** was fully characterized by means of standard spectroscopic techniques.

**Figure 5:**
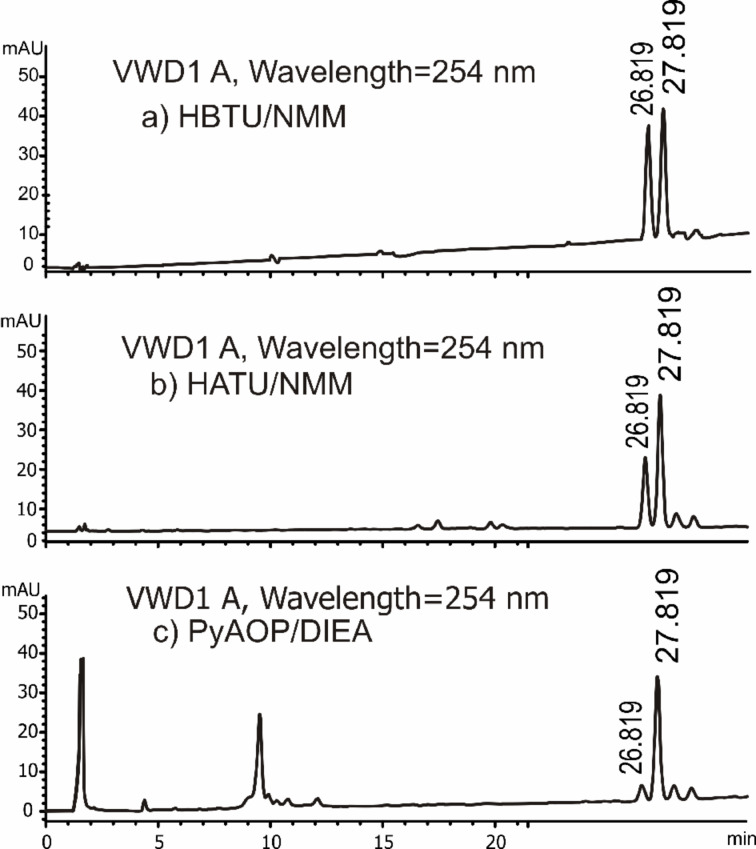
Reverse-phase HPLC chromatograms of the purified fraction obtained from macrocyclization reactions yielding **1** using different coupling agents. The all-*S* cyclic receptor **1** has a retention time of *t*_r_ = 26.8 min, and is the major component in the three analyzed mixtures. The peak with retention time of *t*_r_ = 27.8 min corresponds to the *R*,*R*,*R*,*S*-**1** diastereoisomer.

Initially, we used HBTU/NMM [25,33–34] for activation of the intramolecular peptide bond formation. We observed considerable epimerization at the stereogenic α-carbon. We assessed the coupling reaction using different coupling methods, HATU/NMM [35] and PyAOP/DIEA [36], and found that although the overall reaction yields were independent of the coupling method, the epimerization diminished substantially when the PyAOP/DIEA [35–36] combination was used (Figure 5c).

## Conformational studies

**Figure 6:**
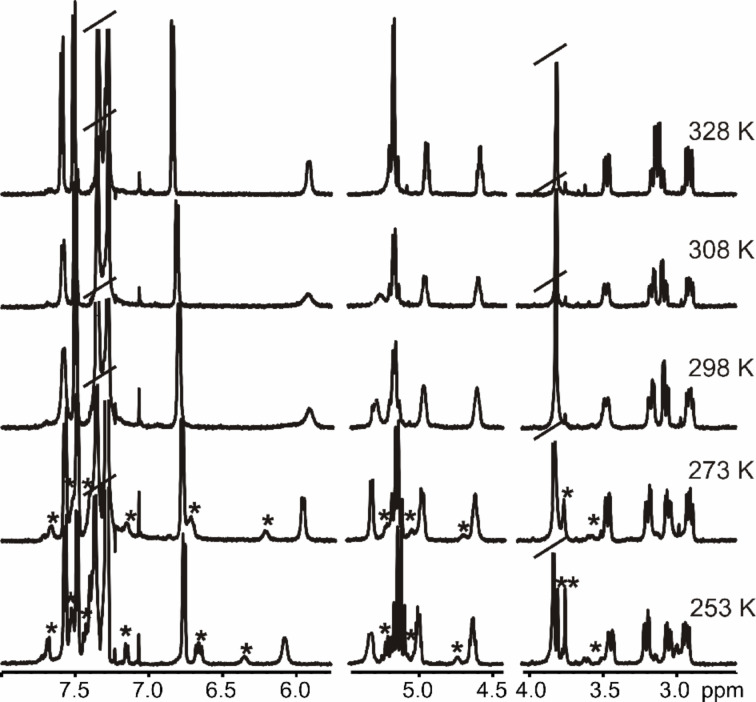
Variable-temperature ^1^H NMR experiments of **1** in chloroform-*d* solution. The proton signals that appeared at low temperature are marked with an asterisk.

The ^1^H NMR spectra of chloroform-*d* solutions of the diastereomerically pure all-*S* cyclic receptors **1** and **2**, as well as those of their linear tetraprotected precursors, **15** and **17**, were temperature-dependent (Figure 6). We attribute this temperature dependence to the existence of conformational equilibria that are in a slow chemical exchange regime with respect to the NMR time scale, i.e., the rotation of the C–N single bond in the carbamate protecting groups [37–38].

**Figure 7:**
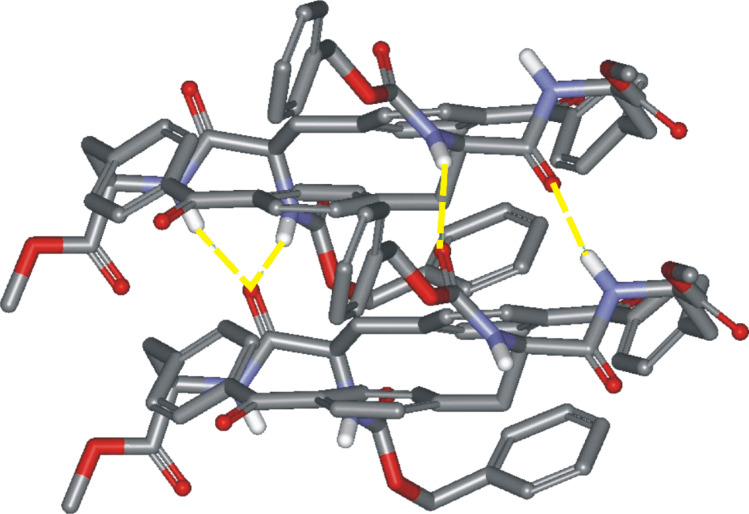
Small fraction of the columnar arrangement observed in solid-state packing of receptor **1**. Two adjacent molecules of **1** interact through the establishment of four hydrogen bonds (yellow dashed lines). For clarity nonpolar hydrogen atoms are omitted.

Upon increasing the temperature of chloroform-*d* solutions of **1**, **2**, **15**, and **17**, the proton signals became sharper and well defined, which is indicative that the chemical exchange due to the conformational equilibria has been accelerated. Conversely, cooling the samples slows down the rate of the chemical exchange. Thus, at low temperature, we observed the appearance of new proton signals that were assigned to different conformations. We observed another general trend in the variable-temperature ^1^H NMR spectra, that is, as the temperature was lowered, the NH signals shifted downfield. This behavior suggested that the cyclic and acyclic peptides may dimerize or oligomerize in chloroform solution through the formation of intermolecular NH···O hydrogen bonds. We have already observed the formation of intermolecular hydrogen bonds in the solid-state structure of receptor **1** (Figure 7).

Before undertaking the study of the binding and molecular recognition properties of the receptor series, and due to their tendency to aggregate in solution, we quantified their dimerization constants in chloroform. The calculation of the dimerization constants relies on the chemical shift changes observed for certain proton signals of the receptors when their ^1^H NMR spectra are acquired at different concentrations. In particular, the receptors’ NH signals shift downfield when the concentration of the solution is increased, indicating the formation of aggregates in the solution that are stabilized through hydrogen bonding. The observed chemical shifts for the NH signals were analyzed mathematically using the HypNMR software and a simple theoretical dimerization binding model [39–40]. We obtained a good fit between the experimental and theoretical data. Additional conclusions can be drawn from the data presented in Table 1. Macrocyclic receptors **1** and **2** show greater tendency to dimerize than their linear precursors. A stronger dimerization tendency for the antiparallel cyclic receptor **1** also becomes apparent.

**Table 1:** Calculated dimerization constant values for the receptor series.

Receptors	*K*_d_ (M^−1^)^a^

**1**	112
**2**	60
**15**	27
**17**	48

^a^Values determined in chloroform-*d* solution at 298 K using ^1^H NMR dilution experiments. All values are associated with at least a 10% error.

**Figure 8:**
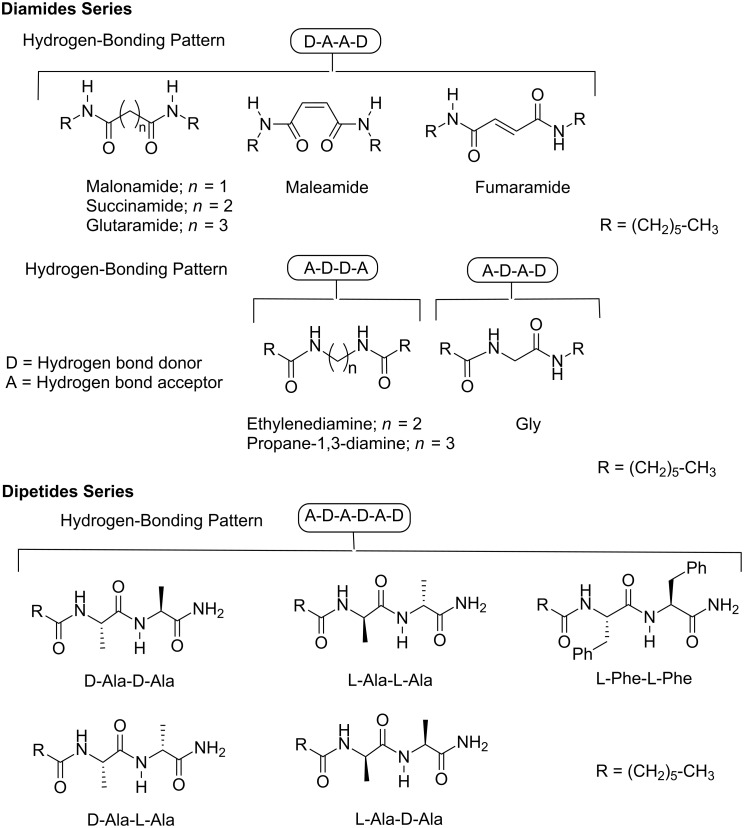
Molecular structures of the guests used in the binding experiments.

We used a wide range of guest molecular structures to examine the molecular recognition properties of receptors **1**, **2**, **15**, and **17** (Figure 8). We selected a series of diamides to evaluate the effect that the hydrogen-bonding pattern produces in the binding affinity. We also investigated the molecular recognition properties of the receptor series with a set of dipeptides. The effect of the size of the amino acid substituents in the dipeptide series (Ala-Ala vs L-Phe-L-Phe) was investigated to shed some light on the geometry of the complexes formed with the cyclic receptors. Finally, the stereoselective recognition properties of the receptors were derived from their binding interactions with the four diastereoisomers of Ala-Ala.

**Figure 9:**
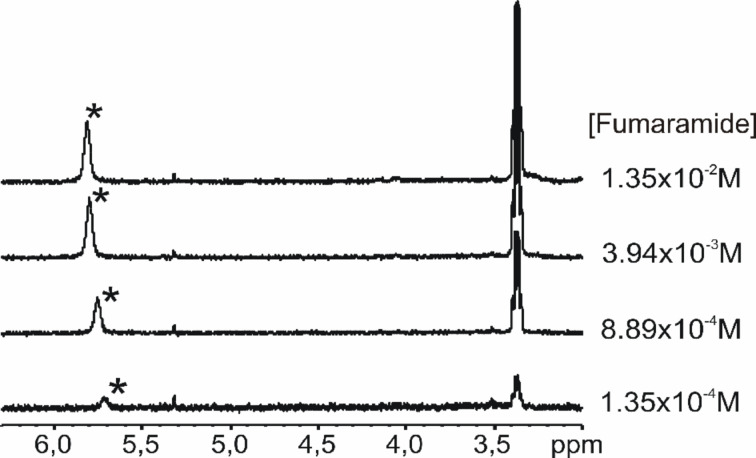
Selected region of the variable-concentration ^1^H NMR spectra acquired using chloroform-*d* solutions of fumaramide. The signal of the NH proton is marked with an asterisk.

The molecular structures of all selected guests have several hydrogen-bonding groups, making them natural candidates to dimerize in solution. Therefore, before studying the interactions of these guests with the receptors, we studied their dimerization behavior in chloroform solution. Using the same methodology described above for the receptors, we calculated the dimerization constants of all guest molecules. The values obtained are summarized in Table 2. With an additional amide group with respect to diamides, the dipeptide dimers can be stabilized by a higher number of hydrogen bonds. The value of the dimerization constant of the diamide of fumaric acid stands out from the rest, likely due to the higher conformational rigidity of this compound (preorganization). Figure 9 depicts the ^1^H NMR spectra acquired in the variable-concentration experiments used for the calculation of the dimerization constant of fumaramide. The NH proton signals experience a significant downfield shift on increasing the concentration of fumaramide.

**Table 2:** Dimerization constant values calculated for the guests used in this study.

Diamides of	*K*_d_ (M^−1^)^a^	Dipeptides	*K*_d_ (M^−1^)^a^

Succinic acid	66	D-Ala-D-Ala	370
Malonic acid	11	L-Ala-L-Ala	346
Ethylenediamine	44	D-Ala-L-Ala	331
Propane-1,3-diamine	44	L-Ala-D-Ala	316
Glutaric acid	<10	L-Phe-L-Phe	346
Maleic acid	31		
Fumaric acid	478		
Gly	22		

^a^Values determined in chloroform-*d* solution at 298 K using ^1^H NMR dilution experiments. All values are associated with at least a 10% error.

**Figure 10:**
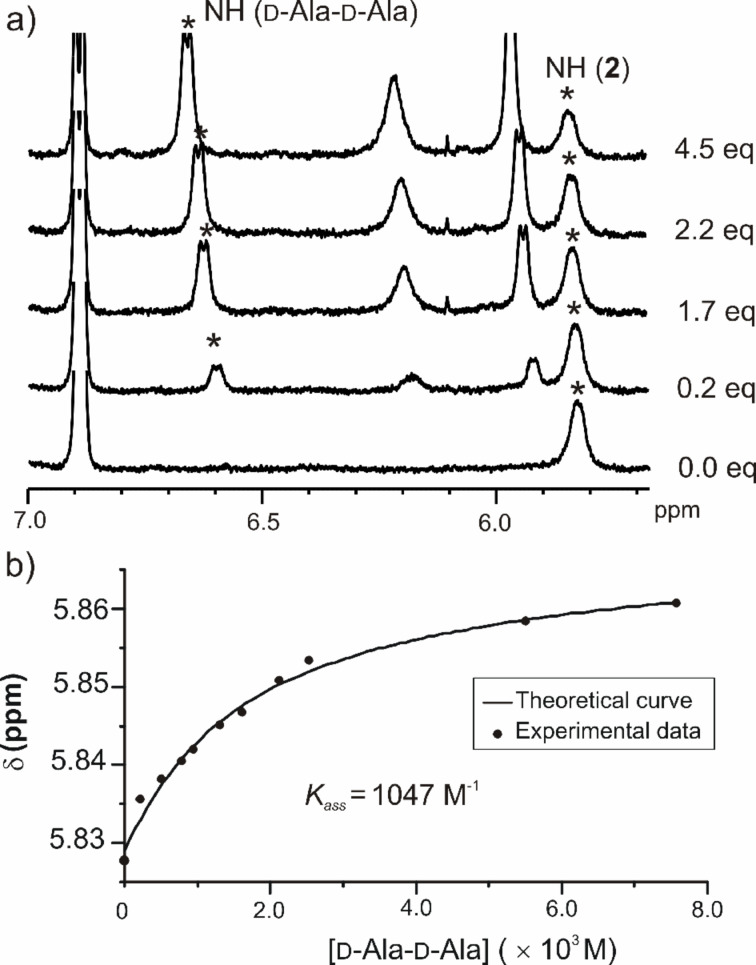
a) Selected region of a series ^1^H NMR spectra acquired during titration of receptor **2** with *n*-C_6_H_13_-D-Ala-D-Ala-NH_2_; b) fit of the experimental data of the titration to the theoretical binding isotherm of the formation of a complex with 1:1 stoichiometry.

Having determined the dimerization tendency of host and guest molecules, we initiated the study of the molecular recognition properties of the receptors toward the different guests. All binding constants were determined using ^1^H NMR titration experiments. As an example, Figure 10a shows a series of spectra acquired during the titration of receptor **2** with *n*-C_6_H_13_-D-Ala-D-Ala-NH_2_. We monitored the chemical shift changes experienced by the NH proton signals of the receptor and of the guest when a 1 mM chloroform-*d* solution of the receptor is treated with incremental amounts of the guest. The titration data were fitted to a theoretical binding model considering the exclusive formation of a 1:1 complex, and the existence of dimeric aggregates of both the receptor and the guest. Figure 10b depicts the experimental data of the titration fitted to the theoretical binding isotherm derived from the above-mentioned theoretical model. The values of the calculated stability constants for the 1:1 complexes are summarized in Table 3 and Table 4.

**Table 3:** Binding constants (*K*_ass_) and free energies of complexation (−Δ*G*^0^ at 298 K) of the 1:1 complexes formed between the cyclic receptors **1** and **2** and the different guests used in this study.

Cyclic receptors	“Antiparallel”	Receptor 1	“Parallel”	Receptor 2
	*K*_ass_^a^ (M^−1^)	−Δ*G*^0^ (kcal/mol)	*K*_ass_^a^ (M^−1^)	−Δ*G*^0^ (kcal/mol)

**Guests** (DAAD)^b^				
Malonamide (1CH_2_)	1380	4.2	398	3.5
Succinamide (2CH_2_)	100	2.7	72	2.5
Glutaramide (3CH_2_)	<5	<1	<5	<1
Maleamide (2CH)	692	3.8	–^c^	–^c^
Fumaramide (2CH)	6309	5.1	7940	5.3
**Guests** (ADDA)^b^				
Ethylenediamine (2CH_2_)	123	2.8	3800	4.8
Propane-1,3-diamine (3CH_2_)	<5	<1	<5	<1
**Guest** (ADAD)^b^				
Gly (1CH_2_)	275	3.3	457	3.6
**Guests** (ADADAD)^b^				
D-Ala-D-Ala	6606	5.2	1047	4.1
L-Ala-L-Ala	3311	4.8	912	4.0
D-Ala-L-Ala	1047	4.1	1148	4.2
L-Ala-D-Ala	1445	4.3	759	3.9
L-Phe-L-Phe	5012	5.0	2089	4.5

^a^All values are associated with at least a 10% error. ^b^Hydrogen-bonding pattern; D = donor, A = acceptor. ^c^Not calculated.

**Table 4:** Binding constants (*K*_ass_) and free energies of complexation (−Δ*G*^0^ at 298 K) of the 1:1 complexes formed between the acyclic receptors **15** and **17** and the different guests used in this study.

Acyclic receptors	“Antiparallel”	**15**	“Parallel”	**17**
	*K*_ass_^a^ (M^−1^)	−Δ*G*^0^ (kcal/mol)	*K*_ass_^a^ (M^−1^)	−Δ*G*^0^ (kcal/mol)

**Guests** (DAAD)^b^				
Malonamide (1CH_2_)	104	2.7	91	2.6
Succinamide (2CH_2_)	<5	<1.0	158	2.9
Glutaramide (3CH_2_)	–^c^	–^c^	126	2.8
Maleamide (2CH)	95	2.6	–^c^	–^c^
Fumaramide (2CH)	973	4.0	7413	5.2
**Guests** (ADDA)^b^				
Ethylenediamine (2CH_2_)	446	3.6	33	2.0
Propane-1,3-diamine (3CH_2_)	78	2.5	–^c^	–^c^
**Guest** (ADAD)^b^				
Gly (1CH_2_)	158	2.9	417	3.5
**Guests** (ADADAD)^b^				
D-Ala-D-Ala	6309	5.2	8318	5.3
L-Ala-L-Ala	1202	4.2	4365	4.9
D-Ala-L-Ala	436	3.6	190	3.1
L-Ala-D-Ala	794	3.9	436	3.6
L-Phe-L-Phe	–^c^	–^c^	–^c^	–^c^

^a^All values are associated with at least a 10% error. ^b^Hydrogen-bonding pattern; D = donor, A = acceptor. ^c^Not calculated.

The analysis of the tabulated data allowed us to draw several conclusions (Table 3 and Table 4). The macrocyclic receptors do not show any affinity for the complexation of diamides in which the two amide groups are spanned by three methylene groups (glutaramide and propane-1,3-diamine). However, these receptors do form complexes with the rest of diamides showing certain degree of selectivity in response to the hydrogen-bonding pattern (Table 3). The antiparallel macrocycle **1** exhibits a moderate preference for the hydrogen-bonding pattern DAAD (D = hydrogen bond donor, A = hydrogen bond acceptor) instead of ADAD when just one methylene group spans the two amide groups (ΔΔ*G*^0^ (malonamide-Gly) = −0.96 kcal/mol). In contrast, parallel receptor **2** effectively discriminates in favor of the hydrogen-bonding pattern ADDA when two methylene groups span the amide groups (ΔΔ*G*^0^ (ethylenediamine-succinamide) = −2.35 kcal/mol). The calculated stability constants are, in general, lower than the values expected for a complex that can be stabilized by an array of not adjacent four hydrogen bonds in chloroform solution (*K* ≈ 10^4^ M^−1^). The stability constant values determined for the complexes formed by both cyclic receptors and fumaramide are more consistent with our estimate. Most likely, the high thermodynamic stability calculated for the complexes of fumaramide in comparison with the rest of diamides resides in the reduced conformational flexibility of the substrate (ΔΔ*G*^0^**_1_** (fumaramide-succinamide) = −2.46 kcal/mol and ΔΔ*G*^0^**_2_** (fumaramide-succinamide) = −2.79 kcal/mol). When the association constant values obtained for the DAAD hydrogen-bonding pattern are compared, it becomes evident that both cyclic receptors exhibited a marked preference for the diamides in which the NH–CO groups are separated by just one methylene group (ΔΔ*G*^0^**_1_** (malonamide-succinamide) = −1.56 kcal/mol and ΔΔ*G*^0^**_2_** (malonamide-succinamide) = −1.01 kcal/mol).

**Figure 11:**
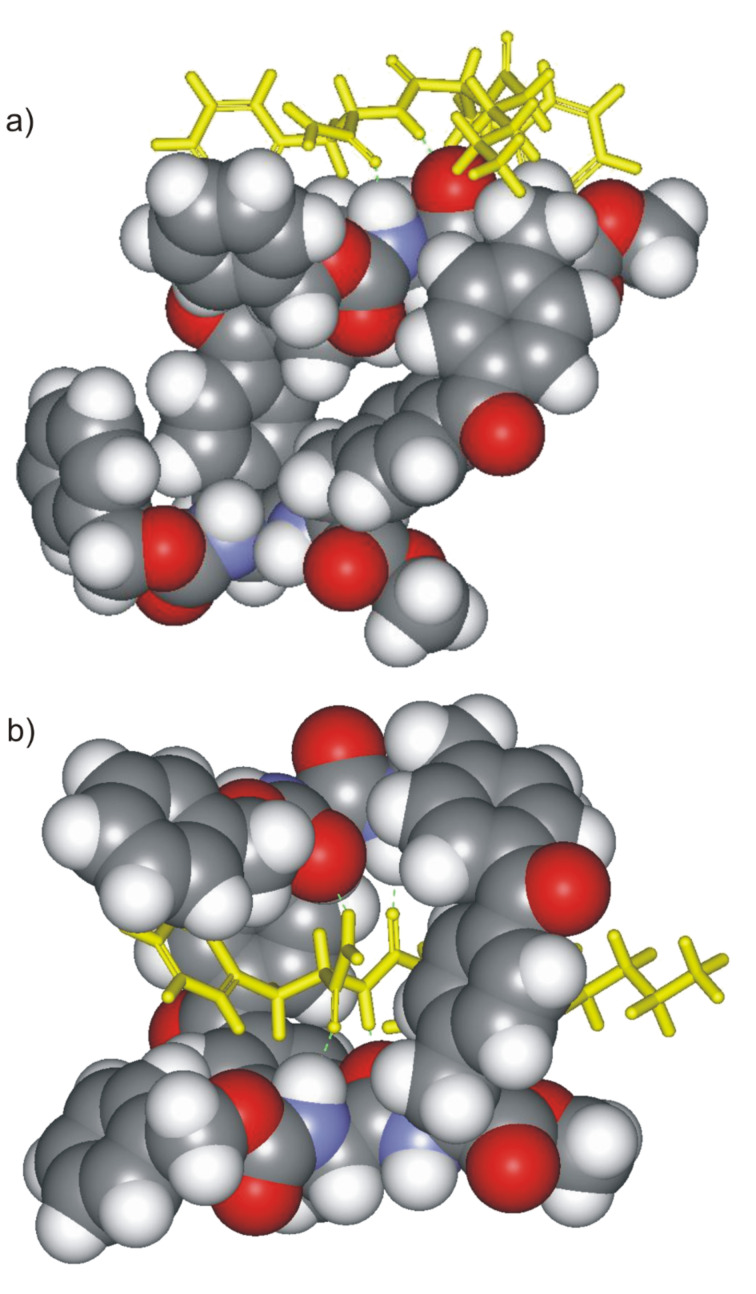
CAChe minimized structures for two possible binding geometries, a) *exo* and b) *endo* complexes formed between receptor **1** and *n*-C_6_H_13_-L-Phe-L-Phe-NH_2_. The macrocyclic receptor is shown as CPK model and the dipeptide in yellow stick representation.

Not surprisingly, the binding affinities calculated for the cyclic and acyclic receptors toward the dipeptide series were higher than those for the diamides. Dipeptides have an additional amide hydrogen-bonding group. The degree of stereoselectivity displayed by the cyclic and acyclic receptors was low (two possible binding geometries for the complexes formed between the macrocyclic receptors and *n*-C_6_H_13_-L-Phe-L-Phe-NH_2_ are shown in Figure 11).

The cyclic antiparallel receptor **1** showed reduced signs of enantioselectivity and moderate diastereoselectivity in the recognition of the enantiomers and diastereoisomers of the Ala-Ala dipeptide (ΔΔ*G*^0^**_1_** (DD-DL) = −1.08 kcal/mol and ΔΔ*G*^0^**_1_** (DD-LD) = −0.89 kcal/mol). The parallel receptor **2** showed neither enantio- nor diastereoselectivity in the recognition of the same substrates (Table 3). The difference in free energy measured for the complexes of **2** with the four diastereoisomers of Ala-Ala was in the order of 0.3 kcal/mol.

We also investigated the complexation affinity of the cyclic receptors toward *n*-C_6_H_13_-L-Phe-L-Phe-NH_2_, with the aim of gaining some information about the geometry of the complex. Molecular modeling suggested that although the formation of an *endo*-complex in which *n*-C_6_H_13_-L-Phe-L-Phe-NH_2_ is threaded through the macrocyclic ring of the receptor is plausible, the steric clashes detected between the dipeptide side chains and the benzophenone linking units should significantly reduce the binding affinity of the cyclic receptors for this substrate or even favor the formation of an alternative complex with *exo*-geometry. Unexpectedly, the stability constant values that we calculated for the 1:1 complexes of the cyclic receptors and *n*-C_6_H_13_-L-Phe-L-Phe-NH_2_ were higher than those for any of the complexes with Ala-Ala (Table 3). Probably, additional intermolecular interactions between the receptors and the phenyl side chains are responsible for the increase in affinity. The low stereoselectivity exhibited by the cyclic receptors, together with the lack of selectivity for the size of the amino acid side chain, encourages us to propose that the geometry of the 1:1 complex is, most likely, *exo*-cyclic. In other words, the dipeptide is not threaded through the cyclophane skeleton of the receptor but bound externally. This hypothesis is also supported by the fact that we were unable to observe upfield shifts in any of the protons of the dipeptide during the binding experiments. The inclusion of the dipeptide in the aromatic cavity of the receptor should produce the shielding of some of its protons due to the anisotropic magnetic current produced by the aromatic rings.

The linear receptors **15** and **17** seem to be more promiscuous in the interaction with the diamides (Table 4). In general the binding affinities are low, except for the fumaramide. The linear receptor **17** shows moderate selectivity for the hydrogen-bonding pattern DAAD instead of ADAD when *n* = 2 (ΔΔ*G*^0^ (succinamide-ethylenediamine) = −0.92 kcal/mol) but selects the hydrogen-bonding pattern ADAD when *n* = 1 (ΔΔ*G*^0^ (Gly-malonamide) = −0.90 kcal/mol).

Surprisingly, linear receptors **15** and **17** exhibited higher levels of stereoselectivity than their cyclic counterparts (Table 4). Receptor **15** displayed the highest enantioselectivity we have measured in the molecular recognition of the D-Ala-D-Ala dipeptide (ΔΔ*G*^0^**_15_** (DD-LL) = −1 kcal/mol) and an acceptable level of diastereoselectivity (ΔΔ*G*^0^**_15_** (DD-DL) = −1.60 kcal/mol). Even higher values of diastereoselectivity were obtained when studying the interaction between the linear receptor **17** and Ala-Ala diastereomers (ΔΔ*G*^0^**_17_** (DD-DL) = −2.18 kcal/mol and ΔΔ*G*^0^**_17_** (DD-LD) = −1.70 kcal/mol). We attribute the surprising and superior stereoselectivity measured for the linear receptors to their higher conformational flexibility compared with the cyclic analogs. This enhanced conformational flexibility allows them to adopt a more effective binding conformation for the sensing of the substrate’s chirality.

## Conclusion

We have designed two macrocyclic receptors for the stereoselective recognition of dipeptides on the basis of the interactions that occur in the β-sheets commonly found in the secondary structure of many biologically relevant proteins. The geometry of the putative complex used in the design of the receptors implies the threading of the dipeptide guest through the macrocyclic skeleton of the receptor. The two designed macrocycles, **1** and **2**, have been synthesized and fully characterized. One of the key synthetic steps, which is common to both synthetic routes, consists in the use of a Stille carbonylative cross-coupling reaction that affords orthogonally tetraprotected 4,4’-bis(alanyl)benzophenone units in good to acceptable yields. Sequential deprotection reactions combined with the formation of two consecutive amide bonds between two units of 4,4’-bis(alanyl)benzophenone produced the macrocyclic receptors in low yield. Notwithstanding the epimerization reactions observed in the formation of the peptide bonds of the macrocyclic structures, both receptors have been isolated as single diastereoisomers. The molecular structure of receptor **1** has been confirmed by single-crystal X-ray diffraction analysis. Although molecular modeling suggested that the cyclic receptors can adopt a conformation with a cavity size large enough to include a peptidic substrate, the X-ray structure obtained for antiparallel receptor **1** shows the collapse of the designed cavity. Although crystal packing may contribute to this conformational change to some degree, the solid-state structure of **1** suggests that the optimal conformation for binding is probably not the lowest-energy conformation. The prepared macrocyclic receptors **1** and **2** as well as their acyclic tetraprotected precursors **15** and **17** show a moderate tendency to aggregate in chloroform solution. Dilution studies carried out at room temperature show that the variation in chemical shift fits a simple theoretical dimerization model, although higher order aggregation cannot be ruled out. Using ^1^H NMR titration experiments we have determined the association constant values of the 1:1 complexes formed between receptors **1**, **2**, **15**, and **17** and a series of diamides and dipeptides. We have observed that each receptor shows different selectivities in the recognition of the hydrogen-bonding patterns present in the diamide series as well as of the number of methylene groups used to separate the two amide functions. However, when the association constant values obtained for the DAAD hydrogen-bonding pattern are compared, it becomes clear that both cyclic receptors exhibited a marked preference for the diamides in which the NH–CO groups are separated by just one methylene group. It is worth noting that a single methylene unit was used as the spacer for the diamide guest used in the receptors’ design. We also investigated the stereoselective recognition properties of the synthesized receptors using the four diastereoisomers of the Ala-Ala dipeptide as guests. The low stereoselectivity displayed by the cyclic receptors, together with their insensitivity to the size of the amino acid chain of the dipeptide guest, allows us to propose that the topology of the 1:1 complexes is not a pseudorotaxane as initially proposed in our design. Most likely, the guests, dipeptides and diamides, bind to the hydrogen-bonding groups that are directed toward the exterior of the aromatic cavity. If macrocyclization results in the receptor adopting a low-energy conformation different from that envisioned in the modeled structures, then preorganization will have created an additional energetic barrier to *endo*-complexation. Finally, the affinity and surprising stereoselectivity exhibited by the linear receptors **15** and **17** are very difficult to rationalize with an *endo*-complex geometry.

We conclude with the caveat that the analysis here pre-supposes that the receptors respond to different ligands with similar binding modes. Due to the complexity of the system, we have not attempted to analyze the possibility that multiple binding modes – *exo*-binding, *endo*-binding – all operate simultaneously and to varying degrees depending on the ligand.

## Supporting Information

Supporting information features experimental procedures, characterization data, NMR spectra of selected compounds and crystallographic data for the solid-state structure of **1**.

File 1 Synthesis and binding studies of two new macrocyclic receptors for the stereoselective recognition of dipeptides

File 2 CIF for the solid-state structure of **1**
