# Supplementary material for: Synthesis and binding studies of two new macrocyclic receptors for the stereoselective recognition of dipeptides
Source: Beilstein J Org Chem. 2010 Jan 19;6:5. doi: 10.3762/bjoc.6.5 (PMC2870531; doi:10.3762/bjoc.6.5)

**SUPPORTING INFORMATION**

Synthesis and binding studies of two new macrocyclic receptors for the stereoselective recognition of dipeptides

Ana Maria Castilla^1^, M. Morgan Conn^2,3^ and Pablo Ballester^*1,4^

Address: ^1^Institute of Chemical Research of Catalonia (ICIQ), Avgda. Països Catalans 16, 43007 Tarragona, Spain; ^2^Amherst College, Amherst, MA 01002, USA; ^3^now at PTC Therapeutics, Inc., 100 Corporate Court, South Plainfield, NJ 07080, USA and ^4^Catalan Institution for Research and Advanced Studies (ICREA), Passeig Lluís Companys, 23, 08018 Barcelona, Spain

Email: Pablo Ballester - pballester@iciq.es

* Corresponding author

**Contents:**

1. General considerations S2

2. Synthesis of 4-iodo-l-phenylalanine S2

3. Synthesis of diprotected 4-iodo-l-phenylalanine derivatives S3–S5

4. Preparation of diprotected stannyl derivatives S6

5. Preparation of bis-alanylbenzophenones S6–S8

6. Cleavage of protecting groups S9–S11

7. Synthesis of linear tetrapeptides S11–S14

8. Synthesis of macrocyclic receptors S15–S16

9. ^1^H and ^13^C NMR spectra S17

**General considerations:** All reagents were obtained from commercial suppliers and used without further purification. All solvents were of HPLC grade quality, obtained commercially and used without further purification. Anhydrous solvents were collected from a solvent purification system SPS-400-6 from Innovative Technologies, Inc. Flash column chromatography was performed with silica gel Scharlab60. ^1^H and ^13^C spectra were recorded on either a Bruker Avance DRX-400 or DRX-500 spectrometer with residual protio solvent as internal standard. Electron spray ionization high resolution mass spectra were obtained on a Waters LCT Premier Mass Spectrometer.

**^1^H NMR titration:** All titrations were carried out on a Bruker 500 MHz spectrometer in CDCl_3_ by using solutions of receptor and adding aliquots of a solution of the substrate (in the same solvent) which was approximately 10 times more concentrated than the receptor solution.

**4-Iodo-l-phenylalanine (7).** A mixture of l-phenylalanine (40.15 g, 243 mmol) in AcOH (200 mL) and concentrated H_2_SO_4_ (29.0 mL, 542 mmol) was stirred while powdered I_2_ (24.65 g, 97.0 mmol) and NaIO_3_ (10.18 g, 51.4 mmol) were added. The mixture was heated to 70 °C for 24 h. After 12 h and after 18 h two more aliquots of NaIO_3_ (2 × 1 g) were added. Completion was indicated by the I_2_ colour fading to orange after 24 h. AcOH was removed by rotary evaporation at 35 °C. The residual viscous oil was diluted with water (400 mL) and washed twice each with 100-mL aliquots of Et_2_O and CH_2_Cl_2_. After decolourization with 5 g of Norit®, the aqueous solution was neutralized with NaOH to precipitate the crude product, which, after chilling, was filtered and rinsed with cold water (800 mL) and then cold ethanol (300 mL). The damp precipitate was recrystallized from AcOH (200 mL) to yield 35.5 g (50%) of 4-iodo-l-phenylalanine as a white solid. mp 249–250 °C; ^1^H NMR (300 MHz, D_2_O/DCl): δ = 7.64 (d, *J* = 8.0 Hz, 2H), 6.94 (d, *J* = 8.0 Hz, 2H), 4.17 (dd, *J* = 5.8, 7.5 Hz, 1H), 3.14 (dd, *J* = 5.8, 14.6 Hz, 1H), 3.03 (dd, *J* = 7.5, 14.6 Hz, 1H); ^13^C NMR (100 MHz, D_2_O/DCl): δ = 170.70, 137.93 (2C), 133.39, 131.21 (2C), 92.88, 53.49, 34.81; IR (KBr, cm^−1^) 2933, 1716, 1699, 1585, 1522, 1488, 1396; MS calcd for C_9_H_11_NO_2_I [M+H]^+^: 291.9844, found 291.9835.

***N*-t-Boc-4-iodo-l-phenylalanine methyl ester (4a).** Thionyl chloride (10.2 g, 0.086 mol) was added dropwise to methanol (10 mL) stirred on an ice bath. Then 4-iodo-l-phenylalanine (5.0 g, 0.017 mol) was added, and the yellow solution was refluxed for 2 h and then rotary evaporated to a white solid. Crystallization from methanol (10 mL) by addition of ether (50 mL) gave 4-iodo-l-phenylalanine methyl ester hydrochloride. This compound (4.5 g, 14.7 mmol) in CH_2_Cl_2_ (25 mL) was treated with *N*-methylmorpholine (4.6 g, 44.5 mmol) and di-*tert*-butyl dicarbonate (4.23 g, 19.2 mmol) at room temperature under inert atmosphere for 5 h. H_2_O was added and CH_2_Cl_2_ removed under reduced pressure. The resulting yellow oil was dissolved in EtOAc, washed with saturated NaHCO_3_, 50 mM citric acid, H_2_O, and saturated NaCl, dried over Na_2_SO_4_, filtered, and rotary evaporated. Crystallization from CH_2_Cl_2_ (10 mL) and hexanes (50 mL) gave Boc derivative of the starting material as a white solid (4.3 g, 61%). mp 75–76 °C; [*α*]_D_^20^ = +47 (*c* 1.0, CH_2_Cl_2_); ^1^H NMR (300 MHz, CDCl_3_): δ = 7.61 (d, *J* = 8.3 Hz, 2H), 6.87 (d, *J* = 8.7 Hz, 2H), 4.96 (d, *J* = 8.4 Hz, 1H), 4.56 (q, *J* = 6.7 Hz, 1H), 3.72 (s, 3H), 3.07 (dd, *J* = 5.9, 14.0 Hz, 1H), 2.97 (dd, *J* = 5.9, 13.8 Hz, 1H), 1.42 (s, 9H); ^13^C NMR (100 MHz, CDCl_3_): δ = 172.03, 155.30, 137.60 (2C), 135.77, 131.34 (2C), 92.52, 80.10, 54.22, 52.29, 37.94, 28.28 (3C); IR (KBr, cm^−1^) 3345, 2958, 1738, 1684, 1525, 1366, 1292, 1162, 1059, 1007; MS calcd for C_15_H_20_NO_4_NaI [M+Na]^+^: 428.0328, found 428.0335.

***N*-[(Benzyloxy)carbonyl]-4-iodo-l-phenylalanine (2-trimethylsilyl)ethyl ester (4c).** To 4-iodo-l-phenylalanine (20.0 g, 68.7 mmol) and NaOH (4.25 g, 106 mmol, 50 mL de H_2_O) in H_2_O (50 mL) were added benzyl chloroformate (13.48 g, 79.0 mmol) and 50 mL of a NaOH solution (4.75 g, 118.8 mmol) in approximately 10 portions alternately over a 30-min period. The cloudy reaction mixture was stirred at room temperature for 1 h until completion. Then it was washed with ether, and concentrated HCl was added to pH 2. Precipitated product was filtered, rinsed with water and hexane, dried in vacuo, and crystallized from 5:1 CH_2_Cl_2_/CH_3_CN to yield 26.43 g (91%) of benzyloxycarbonyl derivative of 4-iodo-l-phenylalanine as a white solid. To a solution of *N*-[(benzyloxy)carbonyl]-4-iodo-l-phenylalanine (2.45 g, 5.78 mmol) in CH_3_CN (20 mL) and pyridine (1.0 mL), on an ice bath, was added 2-(trimethylsilyl)ethanol (0.82 g, 6.94 mmol) and 1,3-dicyclohexylcarbodiimide (DCC) (1.33 g, 6.36 mmol). The mixture was stored in a refrigerator overnight. Oxalic acid 5 M in DMF (0.15 mL) was added and stirred for 1 h to consume any remaining DCC. The precipitate was removed by filtration and washed with EtOAc. The organic filtrate was washed with 0.5 M HCl and saturated NaHCO_3_, dried with MgSO_4_, filtered, and the solvent removed by rotary evaporation. Flash chromatography (EtOAc:hexane 1:9) gave the (trimethylsilyl)ethyl ester as a white solid in 88% yield. mp 45–47 °C; [*α*]_D_^20^ = +24 (*c* 1.0, CH_2_Cl_2_); ^1^H NMR (300 MHz, CDCl_3_): δ = 7.57 (d, *J* = 8.3 Hz, 2H), 7.33 (m, 5H), 6.85 (d, *J* = 8.3 Hz, 2H), 5.42 (d, *J* = 8.1 Hz, 1H), 5.12 (d, *J* = 12.3 Hz, 1H), 5.05 (d, *J* = 12.3 Hz, 1H), 4.60 (dt, *J* = 6.0, 7.9 Hz, 1H), 4.19 (m, 2H), 3.09 (dd, *J* = 5.9, 14.0 Hz, 1H), 2.9 (dd, *J* = 5.9, 14.0 Hz, 1H), 0.94 (m, 2H), 0.053 (s, 9H); ^13^C NMR (100 MHz, CDCl_3_): δ = 171.43, 155.63, 137.60 (2C), 136.32, 135.73, 131.41 (2C), 128.56 (2C), 128.22, 128.11 (2C), 92.60, 66.96, 64.04, 54.80, 37.83, 17.39, −1.44 (3C); IR (KBr, cm^−1^) 3337, 3034, 2954, 1733, 1507, 1250, 1060, 1007, 838, 697; MS calcd for C_22_H_28_NO_4_NaSiI [M+Na]^+^: 548.0731, found 548.0730.

***N*-[(Benzyloxy)carbonyl]-4-iodo-l-phenylalanine *p*-nitrobenzyl ester (4b).** Triethylamine (0.46 mg, 4.5 mmol) was added to a solution of *N*-[(benzyloxy)carbonyl]-4-iodo-l-phenylalanine (1 g, 3 mmol) and 4-nitrobenzyl bromide (0.688 g, 3.15 mmol) in AcOEt (45 mL). The mixture was refluxed at 95 °C overnight. The day after, when the solution was room temperature, it was filtered and washed with AcOEt. The filtrate was washed with aqueous KHSO_4_ 10% (3 × 50 mL), saturated NaHCO_3_ (2 × 20 mL) and water (2 × 50 mL). Following, it was dried over Na_2_SO_4_, filtered and rotary evaporated. The resulting solid was flash chromatographied using as eluent hexane:CHCl_3_ 1:9. **4b** was obtained as a yellow solid (1.18 g, 85%). mp 105–107 °C; [*α*]_D_^28^ = −0.3 (*c* 1.2, CHCl_3_); ^1^H NMR (400 MHz, CDCl_3_): δ = 8.23 (d, *J* = 8.1 Hz, 2H), 7.58 (d, *J* = 8.1 Hz, 2H), 7.36 (m, 7H), 6.84 (d, *J* = 8.1 Hz, 2H), 5.25 (d, *J* = 7.5 Hz, 1H), 5.20 (s, 2H), 5.12 (s, 2H), 4.72 (q, *J* = 6.9 Hz, 1H), 3.07 (d, *J* = 6.36 Hz, 2H); ^13^C NMR (100 MHz, CDCl_3_): δ = 171.09, 155.56, 147.91, 141.92, 139.43, 137.77 (2C), 136.00, 135.11, 131.16 (2C), 128.61 (2C), 128.38 (2C), 128.18 (2C), 123.89 (2C), 92.80, 67.24, 65.69, 54.76, 37.93; IR (ATR, cm^−1^) 3319, 2944, 1745, 1688, 1518, 1342, 1258, 1209, 1005, 1007, 846, 812, 746, 735; MS calcd for C_24_H_21_N_2_O_6_NaI [M+Na]^+^: 583.0342, found 583.0334.

***N*-(9-Fluorenylmethoxycarbonyl)-4-iodo-l-phenylalanine methyl ester (4d).** A solution of Fmoc-OSu in DMF was added to a suspension of 4-iodo-l-phenylalanine (1 g, 3.43 mmol) in aqueous sodium carbonate solution (10%) at 0 °C. The mixture was stirred at r.t. for 2 h. Water was added (200 mL) and it was extracted with AcOEt. The organic layer was washed with HCl 1 N (2×) and water until neutral. The organic layer was washed over Na_2_SO_4_, filtered and rotary evaporated. The solid residue was triturated in a mixture hexane/DCM 3:1. The solid was collected by filtration, washing with hexane and dried in vacuo to obtain pure Fmoc-I-Phe as a white solid. This solid was placed in a round bottom flask and treated with a solution of freshly prepared diazomethane in diethyl ether until persistent yellow colour. The resulting solution was rotary evaporated to obtain the methyl ester of *N*-(9-fluorenylmethylmethoxycarbonyl)-3-(4-iodophenyl)-l-phenylalanine (**4d**) as a white solid (1.02, 61.2%). mp 127–128 °C; [*α*]_D_^29^ = +9.8 (*c* 1.3, CHCl_3_); ^1^H NMR (400 MHz, CDCl_3_): δ = 7.80 (d, *J* = 7.5 Hz, 2H), 7.63 (d, *J* = 8.17 Hz, 2H), 7.59 (dd, *J* = 7.4, 4.3 Hz, 2H), 7.44 (dd, *J* = 7.4, 8.8 Hz, 2H), 7.35 (dd, *J* = 7.4, 8.8 Hz, 2H), 6.84 (d, *J* = 8.12 Hz, 2H), 5.26 (d, *J* = 7.9 Hz, 1H), 4.67 (dd, *J* = 5.5, 7.9 Hz, 1H), 4.49 (dd, *J* = 7.4, 11.03 Hz, 1H), 4.39 (dd, *J* = 6.7, 10.56 Hz, 1H), 4.23 (t, *J* = 6.8 Hz, 1H), 3.68 (s, 3H), 3.12 (dd, *J* = 5.6, 13.9 Hz, 1H), 3.04 (dd, *J* = 5.78, 13.9 Hz, 1H); ^13^C NMR (400 MHz, CDCl_3_): δ = 171.68, 155.47, 143.84, 143.64, 141.42 (2C), 137.69 (2C), 135.43, 131.33 (2C), 127.78 (2C), 127.14 (2C), 125.08 (2C), 120.05 (2C), 92.9, 66.87, 54.56, 52.53, 47.27, 37.79; IR (ATR, cm^−1^) 3336, 2953, 1749, 1686, 1523, 1446, 1260, 1035, 1004, 812, 731; MS calcd for C_25_H_22_NO_4_NaI [M+Na]^+^: 550.0491, found 550.0505.

**General procedure for the synthesis of diprotected trimethylstannyl-l-phenylalanine.** A stirred mixture of diprotected 4-iodo-l-phenylalanine (10 mmol), hexamethylditin Me_3_SnSnMe_3_ (4.59 g, 14 mmol), Pd(OAc)_2_ (90 mg, 0.4 mmol) and PPh_3_ (210 mg, 0.8 mmol) in dry toluene (40 mL) is flushed with Ar for 15 min at room temperature and then heated at 100 °C for 15 min under Ar. The brown mixture is filtered through a short pad of silica gel, diluted with ether. The filtrate is washed twice with water, dried over Na_2_SO_4_ and evaporated. The residue is chromatographied on silica gel.

***N*-Boc-4-trimethylstannyl-l-phenylalanine methyl ester (5a).** The residue obtained following the general method for the reaction of **4a** (4.05 g, 10 mmol) was purified by flash chromatography using as eluent hexanes/EtOAc 92:8. The product was isolated in 84% yield (3.71 g). [*α*]_D_^20^ = +37 (*c* 2.0, CHCl_3_); ^1^H NMR (300 MHz, CDCl_3_): δ = 7.42 (d, *J* = 7.9 Hz, 2H), 7.10 (d, *J* = 7.4 Hz, 2H), 5.02 (d, *J* = 8.1 Hz, 1H), 4.59 (dt, *J* = 6.1, 7.4 Hz, 1H), 3.72 (s, 3H), 3.11 (dd, *J* = 5.5, 13.8 Hz, 1H), 3.01 (dd, *J* = 6.1, 13.8 Hz, 1H), 1.41 (s, 9H), 0.27 (t, *J* = 27.6 Hz, 9H); ^13^C NMR (100 MHz, CDCl_3_): δ = 172.35, 155.12, 140.48, 136.07, 136.00 (2C), 129.06 (2C), 79.75, 54.46, 52.13, 38.26, 28.32 (3C), −9.57 (3C); IR (KBr, cm^−1^) 3366, 1749, 1717, 1507, 1366, 1168.

***N*-[(Benzyloxy)carbonyl]-4-trimethylstannyl-l-phenylalanine *p*-nitrobenzyl ester (5b).** The residue obtained following the general procedure for the reaction of **4b** (1.27 g, 2.27 mmol) was purified by flash chromatography using as eluent hexanes/AcOEt 7:3. **5b** was isolated as an oil (1.0 g, 75%). ^1^H NMR (400 MHz, CDCl_3_): δ = 8.198 (d, *J* = 8.38 Hz, 2H), 7.377 (m, 9H), 7.076 (d, *J* = 7.83 Hz, 2H), 5.24 (s, 3H), 5.13 (s, 2H), 4.75 (dd, *J* = 6.3, 7.8 Hz, 1H), 3.13 (m, 2H), 0.30 (s, 9H); ^13^C NMR (100 MHz, CDCl_3_): δ = 171.06, 154.48, 147.0, 144.3, 141.98, 139.2, 137.2 (2C), 136.00, 134.98, 131.4 (2C), 129 (2C), 128.8 (2C), 127.8 (2C), 124 (2C), 67.28, 65.2, 54.00, 38, −8.9 (3C).

**General procedure for the synthesis of tetraprotected bis-alanyl benzophenones.** A mixture of diprotected 4-iodo-l-phenylalanine (1.0 mmol), diprotected trimethylstannyl-l-phenylalanine (1.1 mmol), PdCl_2_ (0.05 mmol) and PPh_3_ (0.10 mmol) in dry DMF (20 mL), is purged with carbon monoxide for 5 min. After, the mixture is stirred under a CO balloon at 90 °C overnight. The day after the reaction mixture is diluted with EtOAc (100 mL), and stirred at room temperature with saturated KF aqueous solution (3 mL) for 30 min. The precipitate is removed by filtration. The organic filtrate is washed with water (3×), dried over Na_2_SO_4_, filtered and evaporated. The residue is purified by flash chromatography on silica gel.

**Tetraprotected bis-amino acid α-oxodiphenylmethane (3a).** The solid residue obtained by following the general procedure for the reaction of **4c** (1.4 g, 2.66 mmol) and **5a** (1.29 g, 2.93 mmol) was purified by flash chromatography using gradient elution (DCM/AcOEt 100:0 to 90:10). The bis-amino acid **3a** was isolated as a brown solid in 61% yield (1.14 g). mp 54–55 °C; [*α*]_D_^20^ = +46 (*c* 2.0, CHCl_3_); ^1^H NMR (300 MHz, CDCl_3_): δ = 7.78 (dd, *J* = 8.1, 6.3 Hz, 4H), 7.41 (s, 5H), 7.30 (m, 4H), 5.34 (d, *J* = 8.2 Hz, 1H), 5.17 (s, 2H), 5.09 (d, *J* = 7.8 Hz, 1H), 4.73 (m, 2H), 4.28 (m, 2H), 3.81 (s, 3H), 3.25 (m, 4H), 1.49 (s, 9H), 1.06 (d, *J* = 7.5 Hz, 1H), 1.03 (d, *J* = 7.5 Hz, 1H), 0.11 (s, 9H); ^13^C NMR (100 MHz, CDCl_3_): δ = 195.77, 171.99, 171.29, 155.60, 155.02, 141.13, 140.96, 136.36 (2C), 136.21, 130.27 (4C), 129.58 (2C), 129.34 (2C), 128.54 (2C), 128.25, 128.10 (2C), 80.12, 67.04, 64.16, 54.76, 54.25, 52.35, 38.42, 38.28, 28.29 (3C), 17.42, −1.53 (3C); IR (KBr, cm^−1^) 3410, 2954, 1763, 1719, 1708, 1608, 1516, 1251, 1177; MS calcd for C_38_H_48_N_2_O_9_NaSi [M+Na]^+^: 727.3052, found 727.3027.

**Tetraprotected bis-amino acid α-oxodiphenylmethane (3b).** The solid residue obtained by following the general procedure for the reaction of **4c** (1.24 g, 2.36 mmol) and **5b** (1.55 g, 2.59 mmol) was purified by flash chromatography using gradient elution (DCM/AcOEt, 0–6%). The bis-amino acid **3b** was isolated as a brown solid in 53% yield (1.07 g). mp 60–62 °C; [*α*]_D_^26^ = +20.5 (*c* 1.1, CHCl_3_); ^1^H NMR (400 MHz, CDCl_3_): δ = 8.203 (d, *J* = 8.51 Hz, 2H), 7.703 (d, *J* = 8.2 Hz, 2H), 7.683 (d, *J* = 8.13 Hz, 2H), 7.421 (d, *J* = 8.41 Hz, 2H), 7.355 (m, 10H), 7.25 (d, *J* = 7.9 Hz, 2H), 7.218 (d, *J* = 8 Hz, 2H), 5.322 (d, *J* = 8.51 Hz, 1H), 5.292 (d, *J* = 8.51 Hz, 1H), 5.238 (m, 2H), 5.127 (s, 4H), 4.796 (dt, *J* = 6.88, 7.8 Hz, 1H), 4.695 (dt, *J* = 5.8, 7.8 Hz, 1H), 4.243 (m, 2H), 3.23 (m, 4H), 1.003 (t, *J* = 8.7 Hz, 2H), 0.0628 (s, 9H); ^13^C NMR (100 MHz, CDCl_3_): δ = 195.56, 171.262, 171.021, 155.59 (2C), 147.89, 141.971, 141.061, 140.355, 136.631, 136.19, 135.97 (2C), 130.404 (2C), 130.274 (2C), 129.43 (2C), 129.182 (2C), 128.606 (2C), 128.552 (2C), 128.39 (2C), 128.26 (2C), 128.177 (2C), 128.120 (2C), 123.879 (2C), 67.276, 67.059, 65.77, 64.219, 54.73 (2C), 38.26 (2C), 17.42, −1.519 (3C); IR (ATR, cm^−1^) 3329, 3005, 2988, 1716, 1652, 1606, 1519, 1344, 1275, 1260, 1177, 1051, 930, 836, 749, 695; MS calcd for C_46_H_50_N_2_O_14_Si [M+H]^+^: 882.3031, found 882.3019.

**Tetraprotected bis-amino acid α-oxodiphenylmethane (3c).** The solid residue obtained by following the general procedure for the reaction of **4d** (0.75 g, 1.69 mmol) and **5a** (0.98 g, 1.86 mmol) was purified by flash chromatography using gradient elution (DCM/AcOEt, 2–10%). The bis-amino acid **3c** was isolated as a brown solid in 56% yield (0.66 g). mp 125–129 °C; [*α*]_D_^26^ = +49 (*c* 1.0, CHCl_3_); ^1^H NMR (400 MHz, CDCl_3_): δ = 7.78 (d, *J* = 7.54 Hz, 2H), 7.74 (d, *J* = 8.17 Hz, 4H), 7.59 (d, *J* = 7.86 Hz, 2H), 7.42 (t, *J* = 7.86 Hz, 2H), 7.33 (t, *J* = 7.23 Hz, 2H), 7.26 (d, *J* = 8.8 Hz, 2H), 7.22 (d, *J* = 7.8 Hz, 2H), 5.32 (d, *J* = 8.02 Hz, 1H), 5.05 (d, *J* = 7.7 Hz, 1H), 4.74 (dt, *J* = 5.9, 7.7 Hz, 1H), 4.67 (dt, *J* = 6.3, 7.0 Hz, 1H), 4.51 (dd, *J* = 7.36, 11.56 Hz, 1H), 4.42 (dd, *J* = 7.0, 11.56 Hz, 1H), 4.23 (t, *J* = 6.7 Hz, 1H), 3.78 (s, 3H), 3.76 (s, 3H), 3.3–3.09 (m, 4H), 1.44 (s, 9H); ^13^C NMR (100 MHz, CDCl_3_): δ = 195.86, 172.03, 171.61, 155.54, 155.0, 143.67 (2C), 141.38, 141.14, 140.77 (2C), 136.46, 136.32, 130.35 (2C), 130.28 (2C), 129.33 (4C), 127.78 (2C), 127.09 (2C), 125.04 (2C), 120.02 (2C), 80.18, 66.96, 54.59, 54.26, 52.59, 51.41, 47.09, 38.46, 38.25, 28.32 (3C); IR (ATR, cm^−1^) 3329, 2970, 1736, 1654, 1607, 1523, 1447, 1365, 1275, 1260, 1216, 1160, 1053, 930, 838, 794; MS calcd for C_41_H_42_N_2_O_9_Na [M+Na]^+^: 729.2788, found 729.2795.

**Cleavage of TMSE group of 3a (8).** A solution of protected bis-amino acid **3a** (0.625 g, 0.88 mmol) in DMF (3.5 mL) was treated with tetrabutylammonium fluoride 1 M in THF (2.23 mL, 2.23 mmol). The mixture was stirred under inert atmosphere, at r.t. for 1 h. Disappearance of the starting product was followed by TLC (CH_2_Cl_2_/AcOEt 6%). Then, H_2_O and EtOAc were added, and the mixture was acidified with 2 N HCl to pH 3. The organic phase was washed with H_2_O (4×) and saturated NaCl, dried over Na_2_SO_4_, filtered and evaporated to dryness to yield the free carboxylic acid **8** as a white solid (0.457 g, 85.3%). mp 83–85 °C; [*α*]_D_^20^ = +49.5 (*c* 2.0, CHCl_3_); ^1^H NMR (300 MHz, CDCl_3_): δ = 7.75 (m, 4H), 7.40 (m, 3H), 7.32 (m, 6H), 5.34 (d, *J* = 8.1 Hz, 1H), 5.15 (d, *J* = 12.4 Hz, 1H), 5.12 (d, *J* = 12.4 Hz, 1H), 5.07 (d, *J* = 7.1 Hz, 1H), 4.81 (dt, *J* = 6.7, 7.3 Hz, 1H), 4.70 (dt, *J* = 6.7, 7.6 Hz, 1H), 3.78 (s, 3H), 3.26 (m, 4H), 1.48 (s, 9H); ^13^C NMR (100 MHz, CDCl_3_): δ = 196.03, 173.95, 172.03, 155.83, 155.12, 141.17, 140.87, 136.93, 136.36, 136.27, 130.33 (4C), 129.43 (2C), 129.32 (2C), 128.56 (2C), 128.29, 128.11 (2C), 80.28, 67.18, 54.45, 54.24, 52.40, 38.45, 37.85, 28.27 (3C); IR (KBr, cm^−1^) 3345, 2955, 1717, 1652, 1608, 1507, 1287, 1167, 1056; MS calcd for C_33_H_36_N_2_O_9_Na [M+Na]^+^: 627.2334, found 627.2319.

**Cleavage of Boc group of 3a (9).** Protected bis-amino acid **3a** (0.423 g, 0.60 mmol) in TFA/CH_2_Cl_2_ 10% (14.5 mL) was stirred under inert atmosphere at r.t. for 2 h. After, the solution was rotary evaporated to dryness. The residue obtained was dissolved in EtOAc and the solution washed with H_2_O, dried over Na_2_SO_4_ and filtered. Solvent was removed under reduced pressure to yield the amine trifluoracetate **9** (0.384 g, 100%). mp 98–100 °C; [*α*]_D_^20^ = +21 (*c* 2.0, CH_2_Cl_2_); ^1^H NMR (300 MHz, CDCl_3_): δ = 7.65 (d, *J* = 7.7 Hz, 4H), 7.31 (m, 5H), 7.20 (d, *J* = 7.9 Hz, 4H), 5.37 (d, *J* = 8.1 Hz, 1H), 5.07 (m, 2H), 4.64 (m, 1H), 4.35 (t, *J* = 6.6 Hz, 1H), 4.19 (m, 2H), 3.75 (s, 3H), 3.25 (m, 4H), 0.96 (t, *J* = 8.82 Hz, 2H), 0.03 (s, 9H); ^13^C NMR (100 MHz, CDCl_3_): δ = 196.22, 171.41, 169.15, 155.80, 141.58, 138.51, 137.05, 136.12, 135.75, 130.55 (2C), 130.45 (2C), 129.42 (4C), 128.54 (2C), 128.25, 128.06 (2C), 67.09, 64.28, 54.76, 54.14, 53.25, 38.23, 36.04, 17.36, −1.56 (3C); IR (KBr, cm^−1^) 3427, 2956, 1684, 1608, 1540, 1417, 1277, 1203, 1136, 1058; MS calcd for C_33_H_41_N_2_O_7_Si [M+H]^+^: 605.2660, found 605.2683.

**Cleavage of PNB group in 3b (10).** **3b** (0.8 g, 1.43 mmol), tin chloride (II) (5.45 g, 23 mmol) and phenol (0.27 g, 2.8 mmol) were added to a solution of HCl 0.02 M in dioxane (40 mL). The mixture was refluxed at 65 °C overnight. The day after solvents were eliminated by rotary evaporation and the solid residue dissolved in AcOEt. The remaining insoluble solid was filtered off and the solution washed with HCl 1 N, H_2_O and saturated NaCl. The resulting solution was dried over MgSO_4_, filtered and evaporated to dryness to yield **10** as a brown solid (1.72 g, 70%). ^1^H NMR (400 MHz, CDCl_3_): δ = 7.70 (m, 4H), 7.34 (m, 4H), 7.25 (m, 6H), 6.89 (m, 4H), 5.58 (m, 2H), 5.125 (s, 4H), 4.741 (m, 2H), 4.26 (m, 2H), 3.29 (m, 2H), 3.17 (m, 2H), 1.01 (t, *J* = 8.5 Hz, 2H), 0.07 (s, 9H); ^13^C NMR (100 MHz, CDCl_3_): δ = 196.52, 173.92, 171.622 (2C), 156.107 (2C), 141.18 (2C), 136.18, 135.99 (2C), 130.48 (2C), 130.44 (2C), 129.45 (2C), 129.39 (2C), 128.59 (2C), 128.326 (2C), 128.132 (2C), 120.31 (2C), 115.45 (2C), 67.286 (2C), 64.443, 54.56, 54.86, 38.204, 37.83, 17.83, −1.519 (3C); IR (ATR, cm^−1^) 3318, 2952, 1697, 1651, 1605, 1499, 1248, 1178, 1051, 930, 836, 752, 692; MS calcd for C_40_H_44_N_2_O_9_SiNa [M+Na]^+^: 747.2714, found 747.2714.

**Cleavage of Fmoc group in 3c (11).** Piperidine (634 μL, 6.34 mmol) was added dropwise to a solution of **3c** (0.845 g, 1.19 mmol) in CH_2_Cl_2_ while stirred in an ice bath to maintain the mixture at 0 °C. After, the mixture was stirred until room temperature for approximately 30 min. Solvent was removed by rotary evaporation and the solid residue dissolved in ethyl acetate. The organic phase was washed with H_2_O (2×) and dried over MgSO_4_, filtered and rotary evaporated. The solid residue was purified by flash chromatography using gradient elution (hexanes/AcOEt 2:4 to hexanes/AcOEt/MeOH 1:2:20). **11** (*R_f_* = 0.20) was collected and evaporated to dryness to yield a white solid (0.37 g, 64% yield). ^1^H NMR (400 MHz, CDCl_3_): δ = 7.76 (m, 4H), 7.33 (d, *J* = 8.2 Hz, 2H), 7.26 (d, *J* = 8.0 Hz, 2H), 5.05 (d, *J* = 7.9 Hz, 1H), 4.66 (q, *J* = 6.4 Hz, 1H), 3.80 (dd, *J* = 5.1, 7.8 Hz, 1H), 3.76 (s, 6H), 3.19 (m, 3H), 2.97 (dd, *J* = 7.9, 13.5 Hz, 1H), 1.44 (s, 9H); ^13^C NMR (100 MHz, CDCl_3_): δ = 195.85, 171.59 (2C), 154.99, 141.35 (2C), 136.46, 136.19, 130.28 (4C), 129.35 (4C), 80.18, 54.53, 54.33, 52.54, 51.41, 38.48, 38.34, 28.35 (3C); MS calcd for C_26_H_32_N_2_O_7_Na [M+Na]^+^: 507.2231, found 507.5236.

**Acid 14.** Tetrapeptide **12** (0.44 g, 0.369 mmol) dissolved in DMF (8 mL) was treated with tetrabutylammonium fluoride 1 M in THF (0.91 mL, 0.91 mmol). The mixture was stirred under inert atmosphere until disappearance of the starting product (followed by TLC (CH_2_Cl_2_/AcOEt 6%)). Then, H_2_O and EtOAc were added, and the mixture acidified with 2 N HCl to pH 3. The organic phase was washed with H_2_O (4×) and saturated NaCl, dried over Na_2_SO_4_, filtered and evaporated to yield the free carboxylic acid **14** as a white solid (0.371 g, 92%). mp 85–87 °C; [*α*]_D_^20^ = +38.6 (*c* 2.2, CHCl_3_); ^1^H NMR (300 MHz, CDCl_3_): δ = 7.6 (m, 8H), 7.32 (m, 17H), 7.1 (m, 1H), 6.3 (m, 1H), 5.53 (bb, 1H), 5.45 (m, 1H), 5.12 (m, 5H), 4.89 (m, 1H), 4.76 (m, 1H), 4.64 (m, 2H), 3.74 (s, 3H), 3.71 (s, 3H), 3.33–3.06 (m, 8H), 1.43 (s, 9H); ^13^C NMR (100 MHz, CDCl_3_): δ = 195.86 (2C), 171.69 (2C), 171.05, 162.83, 155.97, 155.70, 155.10, 141.19 (4C), 136.31 (4C), 135.96 (2C), 130.29 (8C), 129.29 (8C), 128.55 (4C), 128.26 (2C), 128.08 (4C), 80.22, 67.23, 67.06, 54.31, 53.06, 52.52 (2C), 52.35 (2C), 38.39, 38.21, 37.88, 36.07, 28.27 (3C); IR (KBr, cm^−1^) 3312, 2953, 1699, 1654, 1608, 1540, 1279, 1057; MS calcd for C_61_H_62_N_4_O_15_Na [M+Na]^+^: 1113.4064, found 1113.4109.

**Acid 16.** Tetrapeptide **13** (0.556 g, 0.467 mmol) dissolved in DMF (8 mL) was treated with tetrabutylammonium fluoride 1 M in THF (1.15 mL, 1.15 mmol). The mixture was stirred under inert atmosphere until disappearance of the starting product, followed by reverse phase HPLC (50:50 to 0:100 ACN (0.1% TFA)/water (0.1% TFA) in 10 min). Then, H_2_O and EtOAc were added, and the mixture was acidified with 2 N HCl to pH 3. The organic phase was washed with H_2_O (4×) and saturated NaCl, dried over Na_2_SO_4_, filtered and evaporated to yield the free carboxylic acid **16** as a white solid (0.409 g, 80%). mp 183–185 °C; ^1^H NMR (400 MHz, CDCl_3_): δ = 7.66 (m, 8H), 7.40–7.10 (m, 18H), 6.45 (s, 1H), 5.51 (s, 1H), 5.44 (s, 1H), 5.12 (m, 5H), 4.85 (m, 1H), 4.75 (m, 1H), 4.62 (m, 1H), 4.50 (m, 1H), 3.74 (s, 3H), 3.69 (s, 3H), 3.33–3.04 (m, 8H), 1.43 (s, 9H); ^13^C NMR (100 MHz, CDCl_3_): δ = 195.60, 195.64, 172.1, 171.0, 170.11, 155.50 (2C), 155.00, 141.13, 141.03, 140.49 (2C), 136.50 (2C), 136.30 (2C), 136.18, 135.90, 130.46 (4C), 130.40 (4C), 129.37 (2C), 129.20 (2C), 128.50 (2C), 128.45 (2C), 128.30 (4C), 128.26 (2C), 128.00 (4C), 80.16, 67.39, 67.01, 55.92, 54.72, 54.20, 53.00, 52.59, 52.39, 38.40 (2C), 37.92 (2C), 28.29 (3C); MS calcd for C_61_H_61_N_4_O_15_ [M+H]^+^: 1089.4133, found 1089.4139; *t*_r_ = 7.03 min [Agilent Eclipse XDB-C18, 4.6 × 150 mm, 5 μm; λ = 254 nm; Mobil phase: gradient 50–100% ACN (0.1% TFA) in water (0.1% TFA) in 10 min, flow: 1 mL/min].

**Amino acid 15.** Acid **14** (0.244 g, 0.204 mmol) in TFA/CH_2_Cl_2_ 10% (10 mL) was stirred under inert atmosphere at r.t. for 2 h. Solvents were removed under reduced pressure. The oil obtained was triturated and co-evaporated with diethyl ether twice to remove excess of TFA. Finally, the solid obtained **15** was dried in vacuo (0.217 g, 98%). ^1^H NMR (300 MHz, CDCl_3_): δ = 7.52 (m, 8H), 7.14 (m, 18H), 6.34 (m, 1H), 5.7 (m, 2H), 4.88 (m, 4H), 4.83 (m, 1H), 4.65 (m, 2H), 4.44 (m, 1H), 3.61 (s, 3H), 3.58 (s, 3H), 3.08 (m, 8H); ^13^C NMR (100 MHz, CDCl_3_/MeOD): δ = 196.83 (2C), 172.21, 171.67, 171.42, 168.74, 156.49 (2C), 142.23 (4C), 136.10 (4C), 135.85 (2C), 130.13 (8C), 129.27 (8C), 128.29 (4C), 127.91 (2C), 127.62 (4C), 66.05 (2C), 55.64, 54.76, 53.25 (2C), 52.83, 52.13, 38.73, 37.58, 37.39, 36.05; MS calcd for C_56_H_55_N_4_O_13_ [M+H]^+^: 991.3745, found 991.3766; *t*_r_ = 3.188 min [ZORBAX Eclipse XDB-C18, 4.6 × 150 mm, 5 μm; λ = 254 nm; Mobil phase: ACN (0.1% TFA) 50–100% in water (0.1% TFA) in 10 min, flow: 1 mL/min].

**Amino acid 17.** Acid **16** (0.305 g, 0.279 mmol) in TFA/CH_2_Cl_2_ 10% (12 mL) was stirred under inert atmosphere at r.t. for 3 h. Solvents were removed under reduced pressure. The oil obtained was triturated and co-evaporated with diethyl ether twice to remove excess of TFA. Finally, the solid obtained **17** was dried in vacuo (0.26 g, 94%). mp 141–142 °C; ^1^H NMR (400 MHz, CDCl_3_/MeOD): δ = 7.65 (m, 8H), 7.34–7.12 (m, 18H), 5.12 (m, 4H), 4.83 (m, 1H), 4.73 (m, 1H), 4.60 (m, 1H), 4.48 (m, 1H), 3.74 (s, 3H), 3.70 (s, 3H), 3.33–3.04 (m, 8H); IR (ATR, cm^−1^) 3289, 2952, 1752, 1694, 1655, 1605, 1537, 1262, 1175, 1134, 1053, 930, 835, 766, 692; MS calcd for C_56_H_54_N_4_O_13_Na [M+Na]^+^: 1013.3585, found 1013.3628; *t*_r_ = 3.158 min [ZORBAX Eclipse XDB-C18, 4.6 × 150 mm, 5 μm; λ = 254 nm; Mobil phase: ACN (0.1% TFA) 50–100% in water (0.1% TFA) in 10 min, flow: 1 mL/min].

**Tetraprotected Bis-Dipeptide all-*S*-12.** Trifluoracetate **9** (0.557 g, 0.775 mmol) and acid **8** (0.48 g, 0.794 mmol) were dissolved in anhydrous DMF (10 mL), followed by addition of HATU (0.506 g, 1.30 mmol) and *N*-methylmorpholine (0.173 g, 1.68 mmol). The reaction mixture was stirred at room temperature under inert atmosphere overnight. Then the mixture was dissolved in EtOAc, washed with saturated NaHCO_3_, citric acid 50 mM, H_2_O, and saturated NaCl, dried over Na_2_SO_4_, filtered and rotary evaporated. The residue was purified by flash chromatography using gradient elution (DCM/acetone 96:4 to 9:1) to obtain a mixture of diastereomers of **12** (0.501 g, 55%). Pure diastereomer (17*S*,18*S*,55*S*,56*S*)-**12** was isolated from this mixture by preparative reverse phase HPLC [Column: ZORBAX Eclipse XDB-C18, 4.6 × 150 mm; detector at λ = 254 nm; Mobil phase: gradient 60–90% ACN (0.1% TFA) in water (0.1% TFA), *t* = 30 min, flow: 1 mL/min] in 41% yield (*t*_r_ = 22.3 min). mp 93–95 °C; [*α*]_D_^20^ = +31.6 (*c* 2.15, CH_2_Cl_2_); ^1^H NMR (400 MHz, CDCl_3_): δ = 7.73 (m, 6H), 7.69 (d, *J* = 6.8 Hz, 2H), 7.33 (m, 14H), 7.24 (d, *J* = 7.6 Hz, 2H), 7.17 (d, *J* = 7.6 Hz, 2H), 6.34 (d, *J* = 5.9 Hz, 1H), 5.30 (m, 2H), 5.09 (m, 5H), 4.84 (dt, *J* = 5.9, 7.1 Hz, 1H), 4.67 (m, 2H), 4.45 (m, 1H), 4.23 (m, 2H), 3.75 (s, 3H), 3.71 (s, 3H), 3.29–3.35 (m, 8H), 1.44 (s, 9H), 0.99 (t, *J* = 8.6 Hz, 2H), 0.06 (s, 9H); ^13^C NMR (100 MHz, CDCl_3_): δ = 195.86 (2C), 172.01, 171.31, 171.05, 170.33, 155.67 (3C), 141.18, 141.09 (2C), 140.52, 136.43 (2C), 136.22 (2C), 135.92, 135.74, 130.43 (4C), 130.29 (4C), 129.37 (2C), 129.31 (2C), 129.23 (2C), 128.56 (2C), 128.28 (4C), 128.08 (2C), 127.92 (4C), 80.17, 67.26, 67.03, 64.20, 55.9, 54.72, 54.22, 53.14, 52.58, 52.39, 38.37, 38.21, 37.86 (2C), 28.28 (3C), 17.39, −1.53 (3C); IR (KBr, cm^−1^) 3413, 2952, 1717, 1653, 1608, 1558, 1507, 1280, 1055; MS calcd for C_66_H_74_N_4_O_15_NaSi [M+Na]^+^: 1213.4808, found 1213.4818; *t*_r_ = 6.02 min [Column: ZORBAX Eclipse XDB-C18, 4.6 × 150 mm; detector at λ = 254 nm; Mobil phase: gradient 50–100% ACN (0.1% TFA) in water (0.1% TFA), *t* = 10 min, flow: 1 mL/min].

**Tetraprotected Bis-Dipeptide all-*S*-13.** Amine **11** (0.38 g, 0.78 mmol) and acid **10** (0.625 g, 0.86 mmol) were dissolved in anhydrous DMF (25 mL), followed by addition of HATU (0.511 g, 1.32 mmol) and *N*-methylmorpholine (0.173 g, 1.68 mmol). The reaction mixture was stirred at room temperature under inert atmosphere overnight. Then the mixture was dissolved in EtOAc, washed with saturated NaHCO_3_, citric acid 50 mM, H_2_O, and saturated NaCl, dried over Na_2_SO_4_, filtered and rotary evaporated. The residue was purified by flash chromatography using gradient elution (DCM/acetone 98:2 to 9:1) to obtain (17*S*,18*S*,47*S*,48*S*)-**13** 95% pure (0.616 g, 66%). mp 93–95 °C; [*α*]_D_^28^ = +33 (*c* 1.2, CHCl_3_); ^1^H NMR (400 MHz, CDCl_3_): δ = 7.71 (m, 8H), 7.34 (m, 10H), 7.27 (d, *J* = 7.9 Hz, 4H), 7.24 (d, *J* = 8.6 Hz, 2H), 7.17 (d, *J* = 7.05 Hz, 2H), 6.38 (d, *J* = 6.7 Hz, 1H), 5.32 (d, *J* = 6.7 Hz, 2H), 5.10 (m, 4H), 5.06 (m, 1H), 4.85 (dt, *J* = 6.0, 7.3 Hz, 1H). 4.66 (m, 2H), 4.65 (m, 2H), 4.46 (m, 1H), 4.23 (m, 2H), 3.74 (s, 3H), 3.7 (s, 3H), 3.29–3.06 (m, 8H), 1.44 (s, 9H), 0.99 (t, *J* = 8.6 Hz, 2H), 0.06 (s, 9H); ^13^C NMR (100 MHz. CDCl_3_): δ = 195.77, 195.68, 172.0, 171.28, 170.99, 170.10, 155.59 (2C), 155.01, 141.17, 141.00, 140.45 (2C), 136.48 (2C), 136.26 (2C), 136.18, 135.92, 130.45 (4C), 130.30 (4C), 129.37 (2C), 129.22 (2C), 128.59 (2C), 128.55 (2C), 128.34 (4C), 128.26 (2C), 128.10 (4C), 80.16, 67.29, 67.0, 64.21, 55.91, 54.71, 54.24, 53.12, 52.59, 52.39, 38.42, 38.28, 37.92 (2C), 28.30 (3C), 17.42, −1.52 (3C); IR (ATR, cm^−1^) 3336, 2951, 1709, 1655, 1606, 1509, 1276, 1175, 1049, 929, 836, 694; MS calcd for C_66_H_74_N_4_O_15_NaSi [M+Na]^+^: 1213.4818, found 1213.4868; *t*_r_ = 28.1 min [ZORBAX Eclipse XDB-C18, 4.6 × 150 mm, 5 μm; λ = 254 nm; Mobil phase: gradient 60–80% ACN (0.1% TFA) in water (0.1% TFA) in 30 min, flow: 1 mL/min].

**Macrocyclic receptor all-*S*-1.** PyAOP (0.16 g, 0.30 mmol) and DIEA (0.74 mL, 0.81 mmol) were dissolved in anhydrous DMF (100 mL). This solution was stirred under inert atmosphere while a solution of **15** (0.20 g, 0.20 mmol) in anhydrous DMF (10 mL) was added dropwise through a syringe pump for 12 h. After that period, DMF was removed under reduced pressure heating at 40 °C. When almost DMF was removed AcOEt was added. The resulting solution was washed with saturated NaHCO_3_, citric acid 50 mM, water and saturated NaCl. The solution was dried over Na_2_SO_4_, filtered and the filtrate evaporated to dryness. The resultant residue was dissolved in DCM causing the precipitation of a white solid which was filtered off. The filtrate was rotary evaporated to dryness. The resulting solid was purified by flash chromatography using as eluent DCM/acetone 9:1 (*R_f_* = 0.68). The fraction collected was purified by preparative normal phase HPLC [Column: “Waters Spherisorb® S5W, 20 × 250 mm, Semi-Prep Column”; λ = 254 nm; Mobil phase: isocratic DCM/EtOH 98:2, 30 min, flow: 17 mL/min; sample concentration: 5 mg/mL in DCM; volume injection 1.4 mL] collecting the product with *t*_r_ = 10.9 min. This fraction was evaporated to dryness to obtain (16*S*,20*S*,38*S*,42*S*)-**1** as a white solid (0.41 g, 21%). mp 241.5–242.5 °C; [*α*]_D_^29^ = +80 (*c* 1.0, CHCl_3_); ^1^H NMR (400 MHz, CDCl_3_): δ = 7.58 (d, *J* = 6.2 Hz, 4H), 7.50 (d, *J* = 7.9 Hz, 4H), 7.35 (s, 10H), 7.30 (m, 4H), 6.80 (d, *J* = 7.9 Hz, 4H), 5.92 (m, 2H), 5.29 (m, 2H), 5.17 (m, 4H), 4.97 (m, 2H), 4.61 (m, 2H), 3.82 (s, 6H), 3.48 (d, *J* = 12.9 Hz, 2H), 3.18 (dd, *J* = 4.8, 13.6 Hz, 2H), 3.07 (dd, *J* = 5.1, 13.9 Hz, 2H), 2.91 (m, 2H); ^13^C NMR (100 MHz, CDCl_3_): δ = 194.63 (2C), 170.83 (2C), 169.78 (2C), 155.85 (2C), 140.05 (4C), 136.29 (2C), 135.89 (4C), 130.52 (4C), 130.37 (4C), 129.62 (4C), 128.88 (2C), 128.68 (4C), 128.48 (4C), 128.26 (4C), 67.54 (2C), 55.70 (4C), 52.60, 52.25, 38.13 (2C), 37.13 (2C); IR (ATR, cm^−1^) 3293, 2953, 1732, 1690, 1650, 1607, 1531, 1438, 1256, 1177, 1037, 929, 753, 697; MS calcd for C_56_H_52_N_4_O_12_Na [M+Na]^+^: 995.3479, found 995.3500; *t*_r_ = 10 min [Column ZORBAX Eclipse XDB-C18, 4.6 × 150 mm, 5 μm; λ = 254 nm; Mobil phase: gradient 55–65% ACN (0.1% TFA) in water (0.1% TFA) in 30 min, flow: 1 mL/min]. Anal. calcd for C_56_H_52_N_4_O_12_: C 69.1287%; H 5.39%; N 5.76%; O 19.70%; found: C 69.12%; H 5.39%; N 5.76%; O 19.73%.

**Macrocyclic receptor all-*S*-2.** PyAOP (0.231 g, 0.425 mmol) and DIEA (0.148 mL, 1.13 mmol) were dissolved in anhydrous DMF (150 mL). This solution was stirred under inert atmosphere while a solution of **17** (0.313 g, 0.283 mmol) in anhydrous DMF (10 mL) was added dropwise through a syringe pump for 20 h. After that period, DMF was removed under reduced pressure heating at 40 °C. When almost all DMF was removed AcOEt was added. The solution was washed with saturated NaHCO_3_, citric acid 50 mM, water and saturated NaCl. The solution was dried over Na_2_SO_4_, filtered and the filtrate evaporated to dryness. The resultant residue was dissolved in DCM (8 mg/mL) and purified by preparative normal phase HPLC [Column: “Waters Spherisorb® S5W, 20 × 250 mm, Semi-Prep Column”; λ = 254 nm; Mobil phase: isocratic DCM/EtOH 98:2, 30 min, flow: 17 mL/min; volume injection 1.4 mL] collecting the product with *t*_r_ = 10.56 min. This fraction was evaporated to dryness to obtain (15*S*,19*S*,36*S*,40*S*)-**2** as a white solid (0.69 g, 25%). mp 235.6–236.7 °C; [*α*]_D_^28^ = +14 (*c* 1.3, CHCl_3_); ^1^H NMR (400 MHz, CDCl_3_): δ = 7.61 (d, *J* = 8.2 Hz, 4H), 7.51 (d, *J* = 8.2 Hz, 4H), 7.40 (s, 10H), 7.31 (m, 4H), 6.88 (d, *J* = 8.2 Hz, 4H), 5.83 (m, 2H), 5.49 (m, 2H), 5.21 (d, *J* = 12.17 Hz, 2H), 5.17 (d, *J* = 12.17 Hz, 2H), 4.86 (q, *J* = 6.3 Hz, 2H), 4.53 (dt, *J* = 2.9, 8.4 Hz, 2H), 3.81 (s, 6H), 3.46 (dd, *J* = 1.47, 12.32 Hz, 2H), 3.13 (m, 4H), 2.91 (dd, *J* = 10.2, 11.7 Hz, 2H); ^13^C NMR (100 MHz, CDCl_3_): δ = 194.68 (2C), 170.86 (2C), 170.03 (2C), 155.83 (2C), 140.08 (4C), 136.20 (2C), 135.94 (4C), 130.55 (4C), 130.36 (4C), 129.57 (4C), 128.81 (2C), 128.68 (4C), 128.47 (4C), 128.28 (4C), 67.43 (2C), 55.88 (4C), 52.63 (2C), 38.52 (2C), 37.00 (2C); IR (ATR, cm^−1^) 3290, 2949, 1727, 1692, 1647, 1607, 1525, 1441, 1240, 1178, 1013, 929, 752, 695; MS calcd for C_56_H_52_N_4_O_12_Na [M+Na]^+^: 995.3479, found 995.3453; *t*_r_ = 17 min [Column ZORBAX Eclipse XDB-C18, 4.6 × 150 mm, 5 μm; λ = 254 nm; Mobil phase: gradient 50–60% ACN (0.1% TFA) in water (0.1% TFA) in 30 min, flow: 1 mL/min].

***N*-t-Boc-4-iodo-l-phenylalanine methyl ester (4a)**

^1^H NMR, 300 MHz, CDCl_3_

**`**
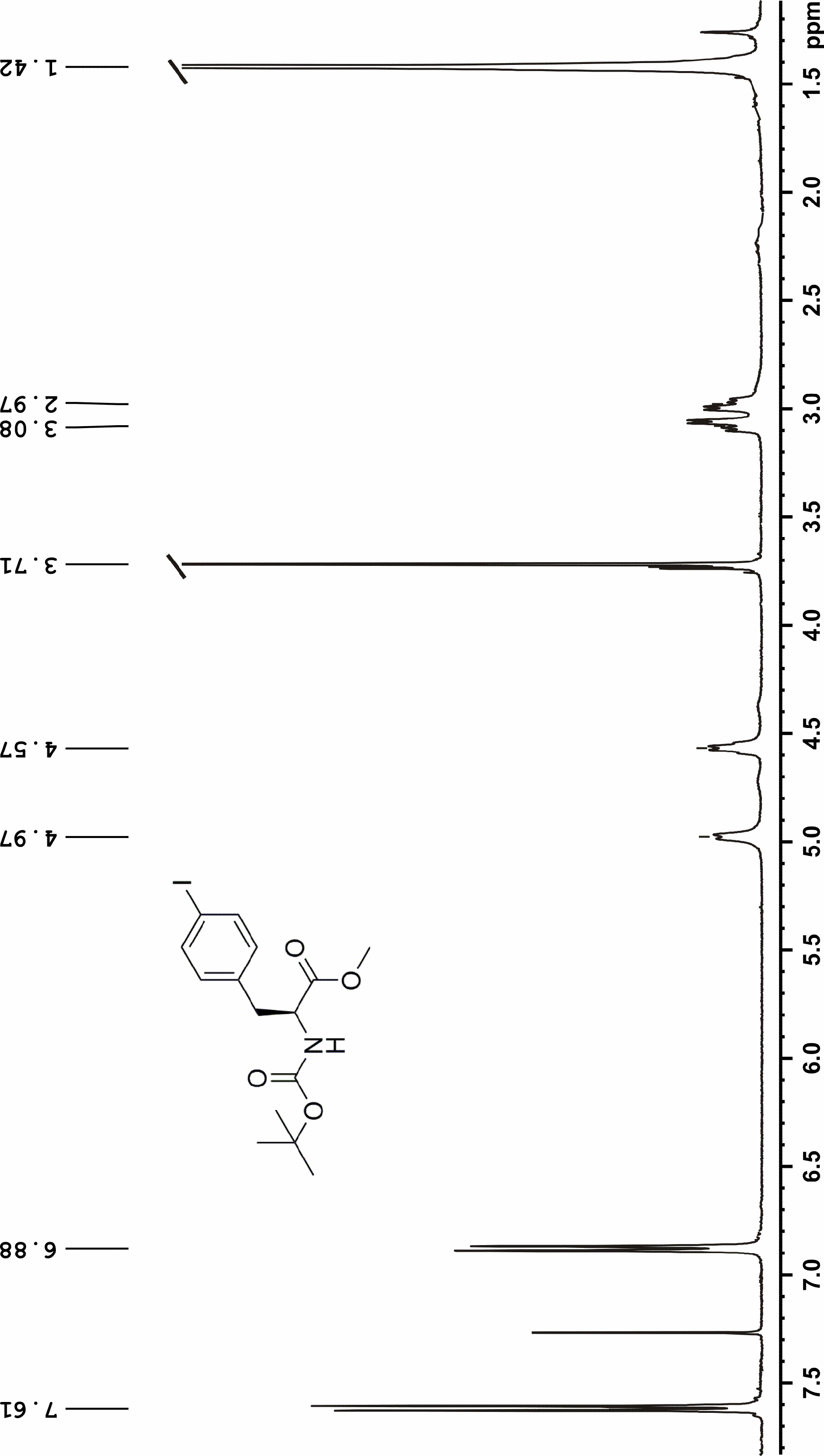


^13^C NMR, 100 MHz, CDCl_3_


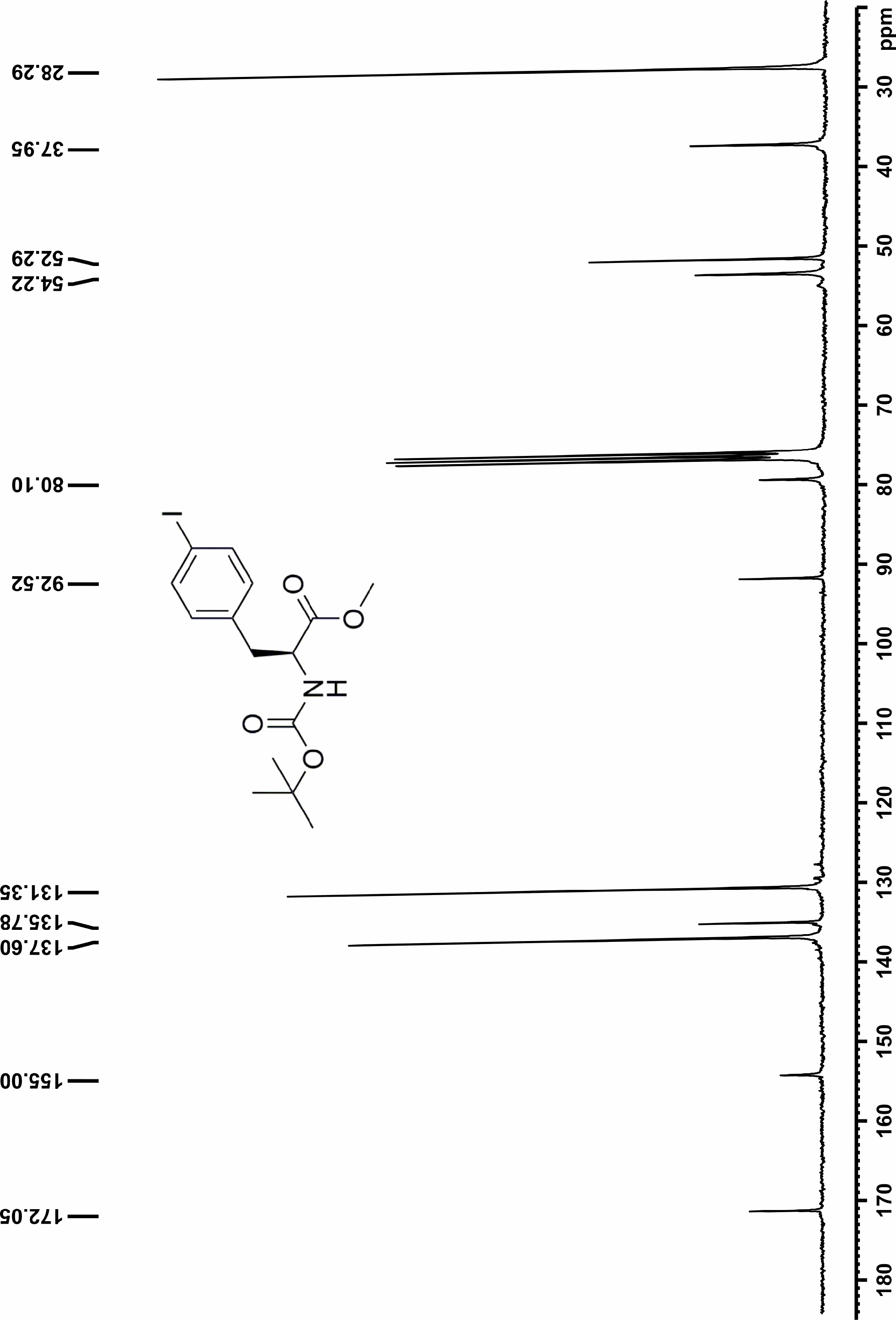


***N*-[(Benzyloxy)carbonyl]-4-iodo-l-phenylalanine (2-trimethylsilyl)ethyl ester (4c)**

^1^H NMR, 300 MHz, CDCl_3_


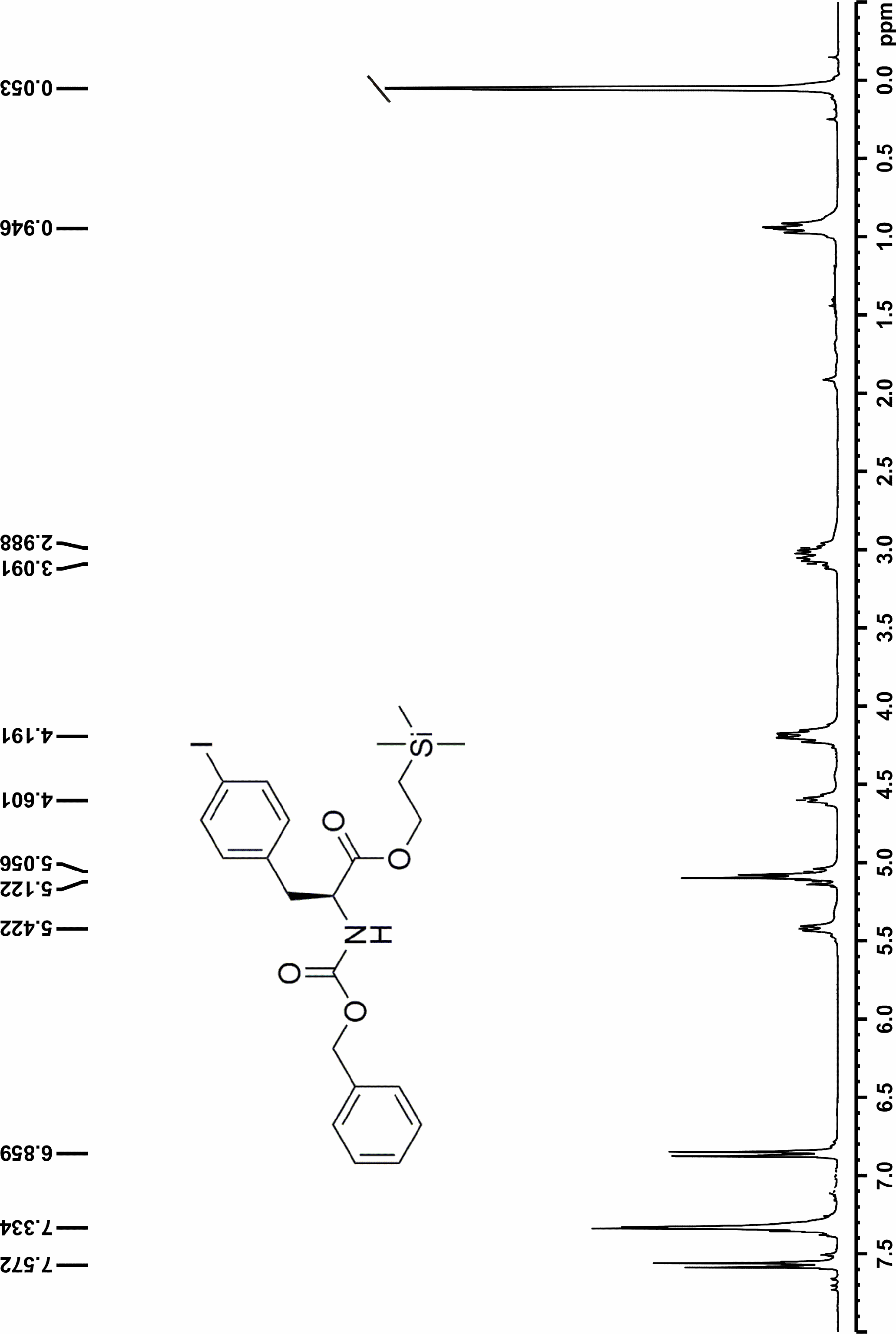


^13^C NMR, 100 MHz, CDCl_3_


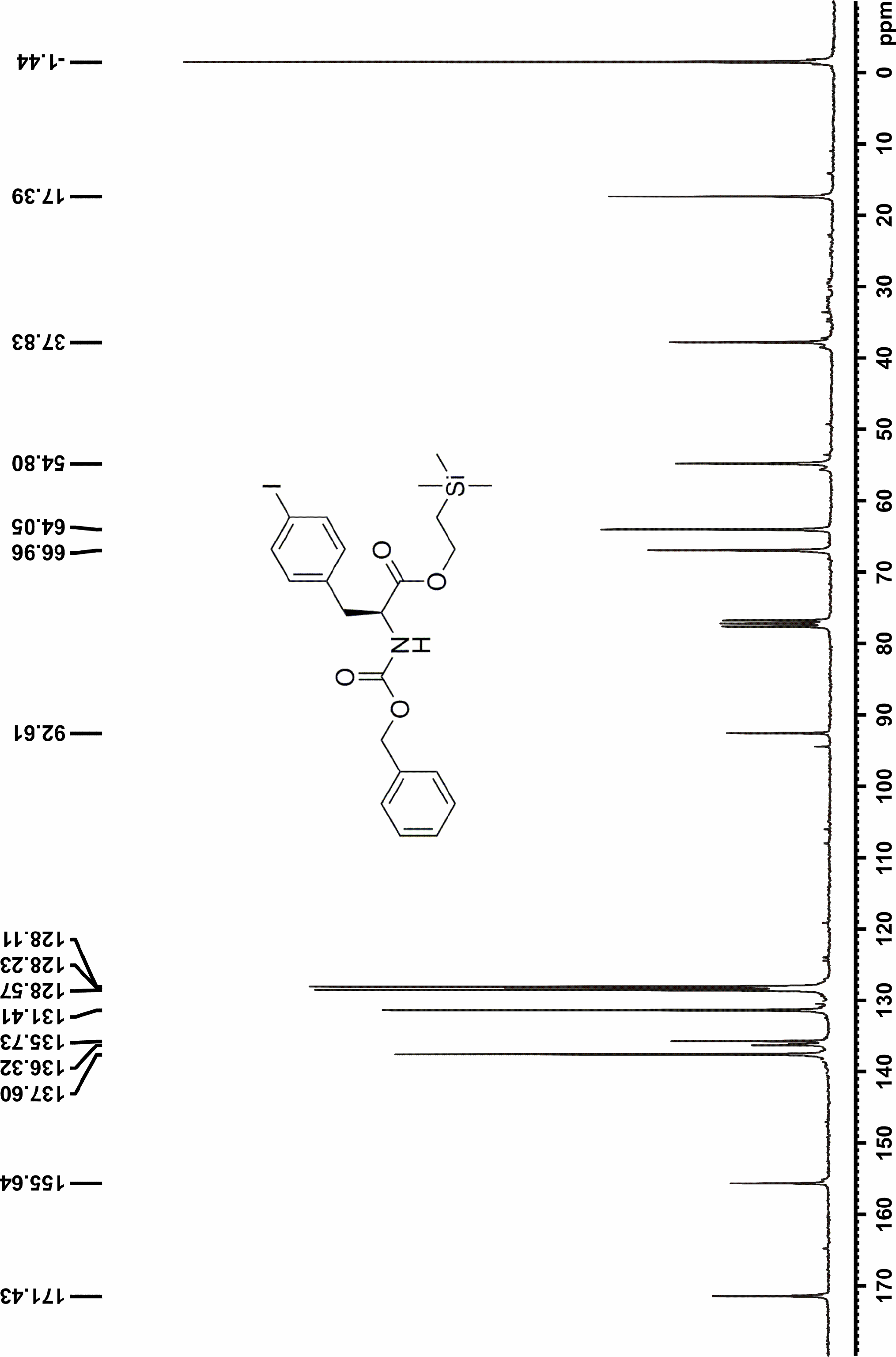


***N*-[(Benzyloxy)carbonyl]-4-iodo-l-phenylalanine *p*-nitrobenzyl ester (4b)**

^1^H NMR, 400 MHz, CDCl_3_


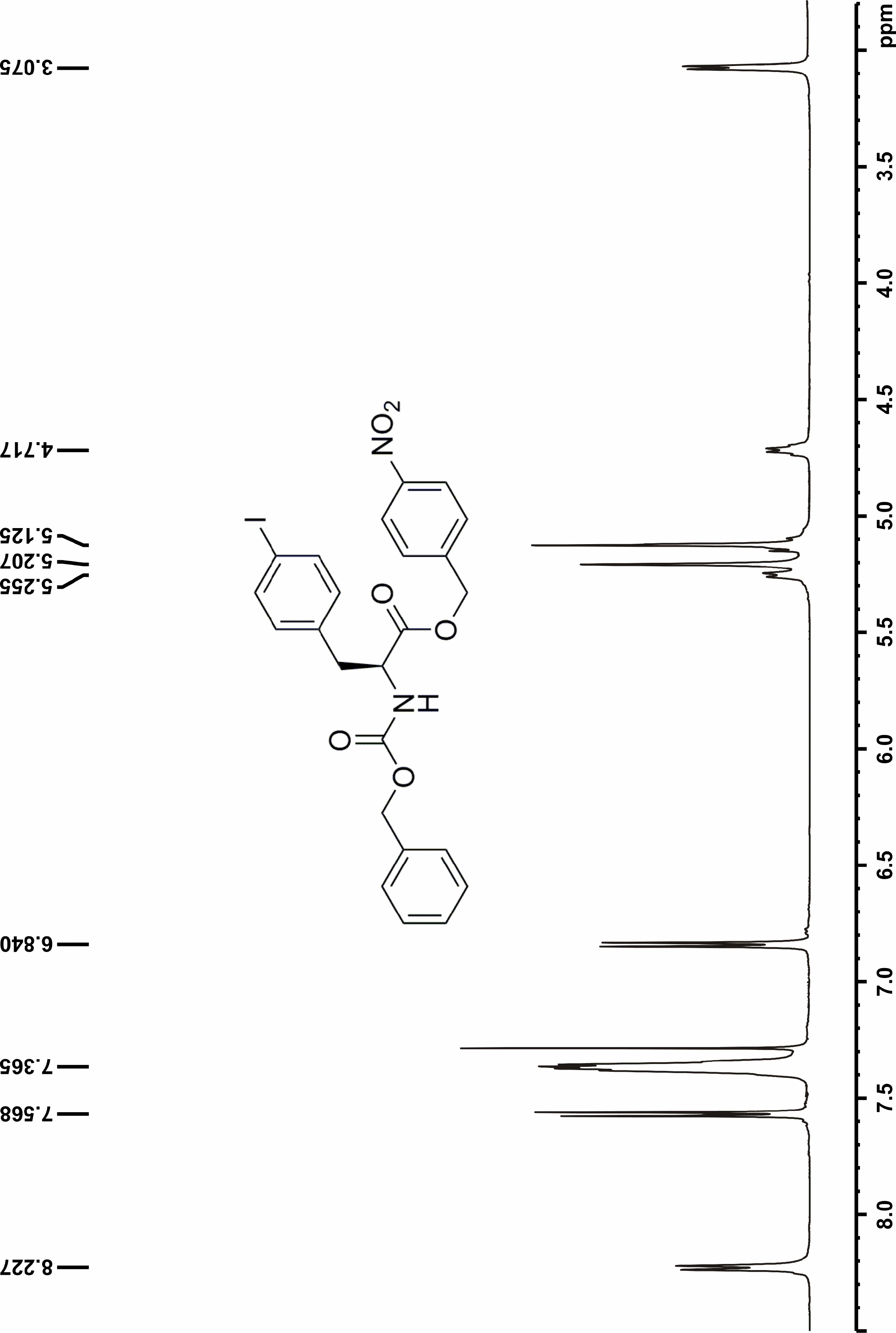


^13^C NMR, 100 MHz, CDCl_3_


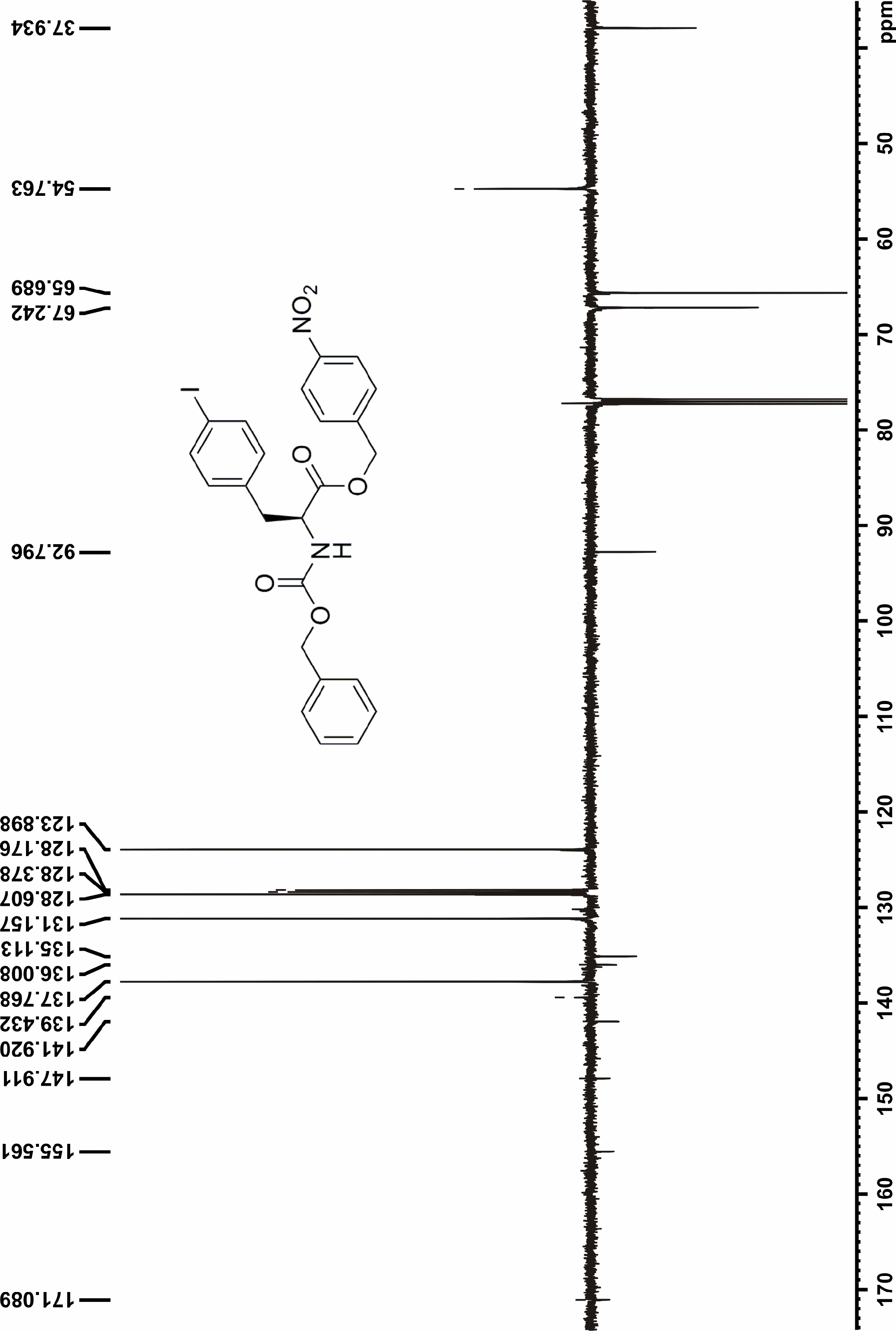


***N*-(9-Fluorenylmethoxycarbonyl)-4-iodo-l-phenylalanine methyl ester (4d)**

^1^H NMR, 400 MHz, CDCl_3_


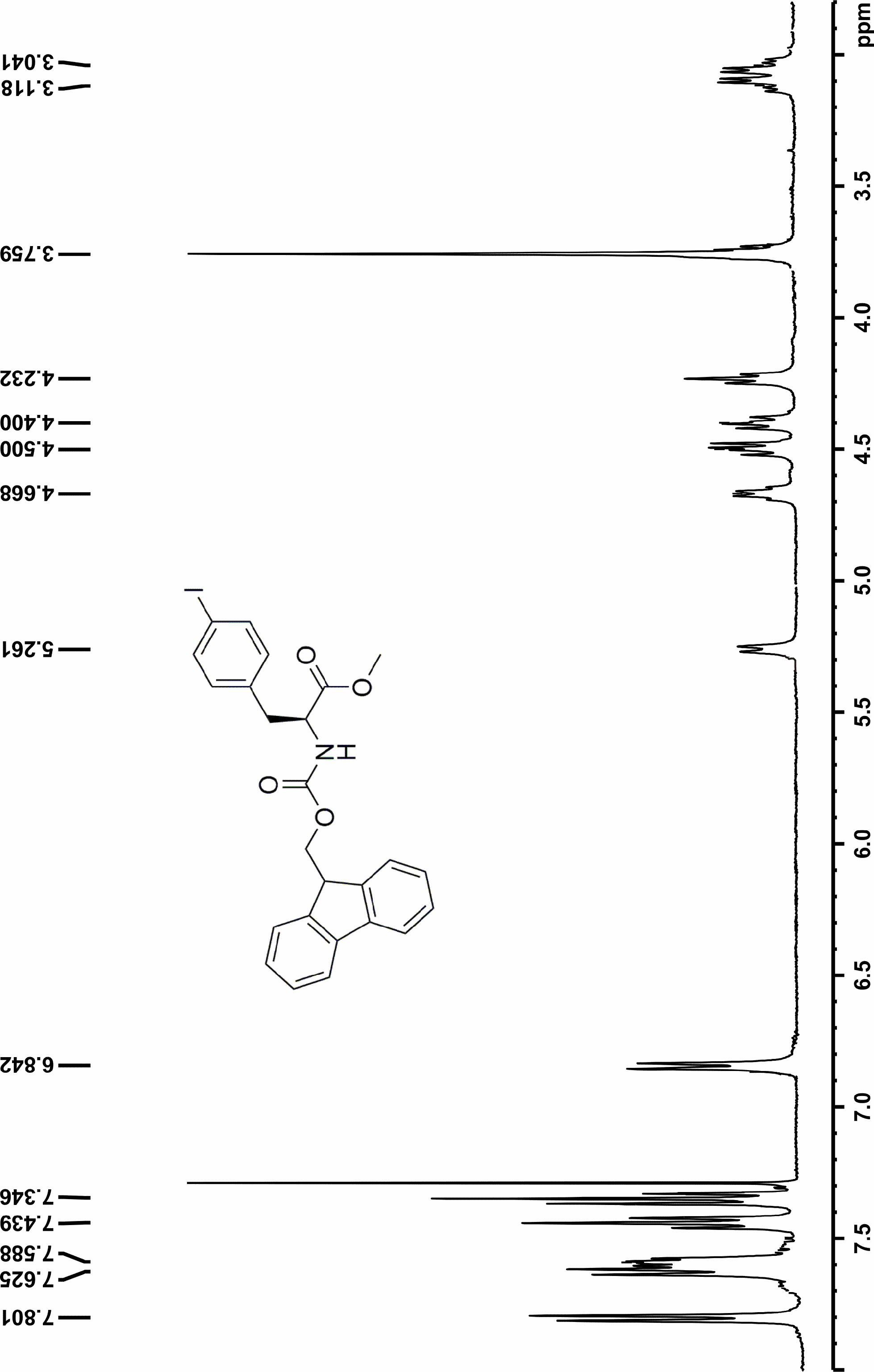


^13^C NMR, 400 MHz, CDCl_3_


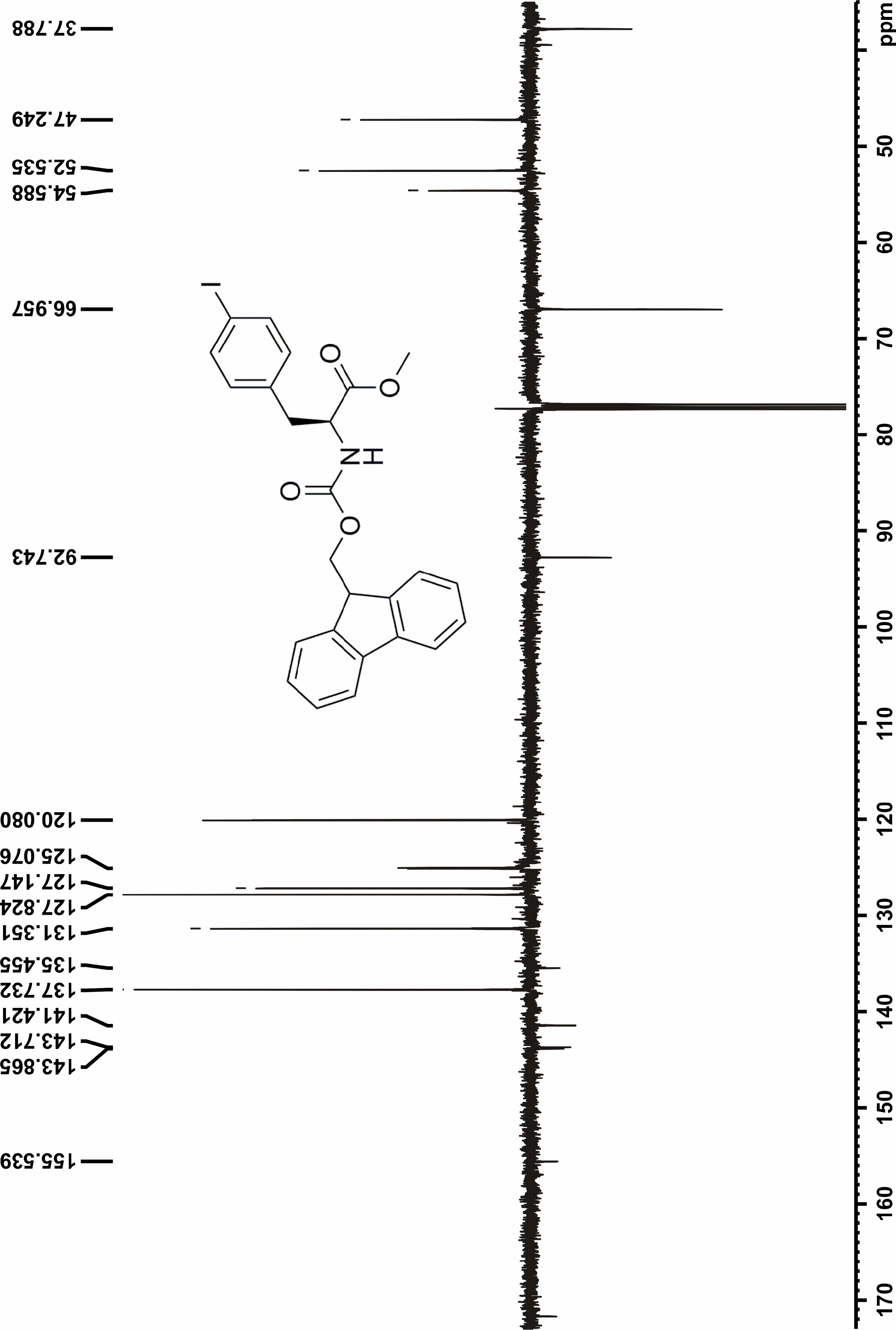


***N*-Boc-4-trimethylstannyl-l-phenylalanine methyl ester (5a)**

^1^H NMR, 300 MHz, CDCl_3_


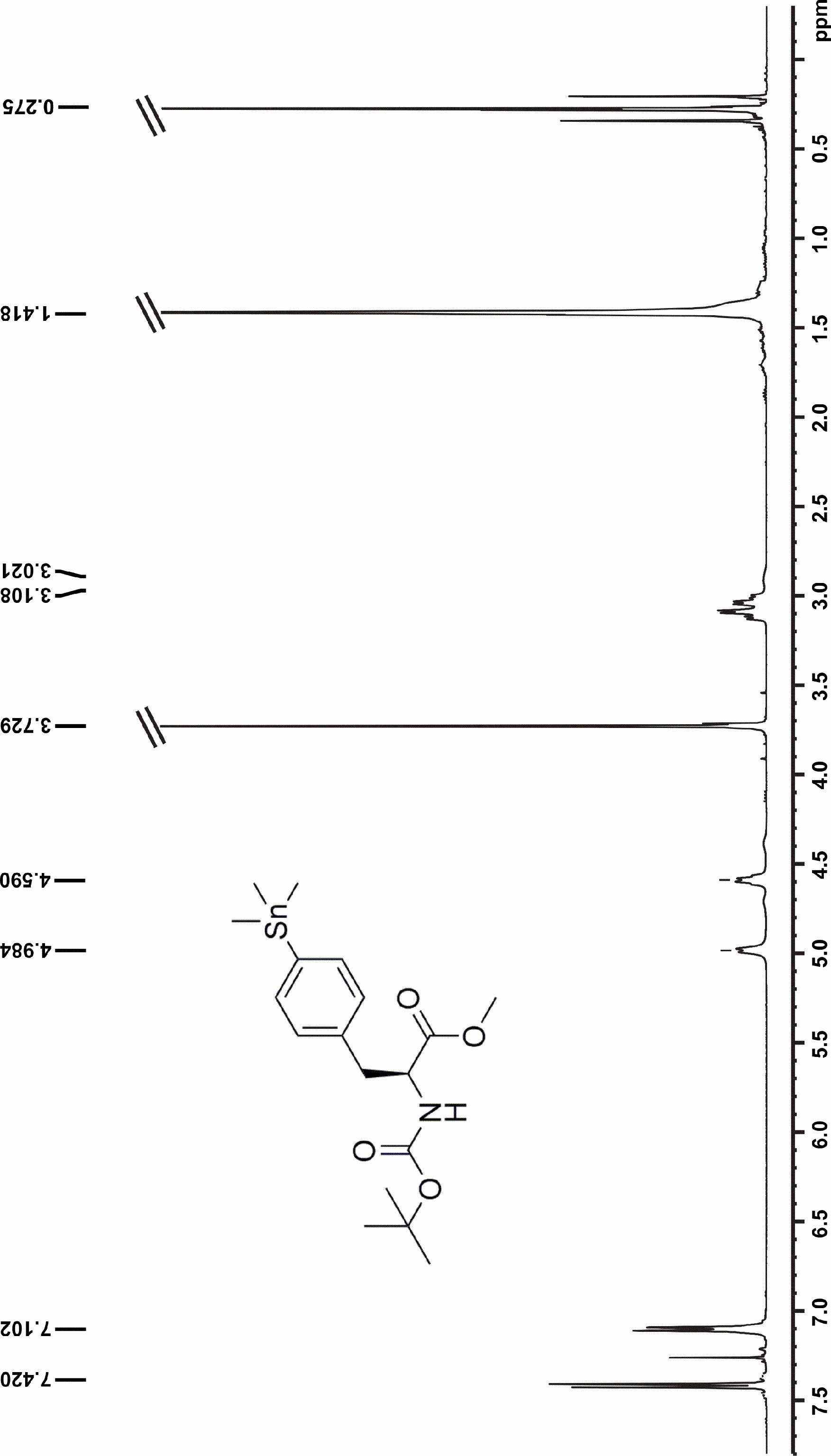


***N*-[(Benzyloxy)carbonyl]-4-trimethylstannyl-l-phenylalanine *p*-nitrobenzyl ester (5b)**

^1^H NMR, 400 MHz, CDCl_3_


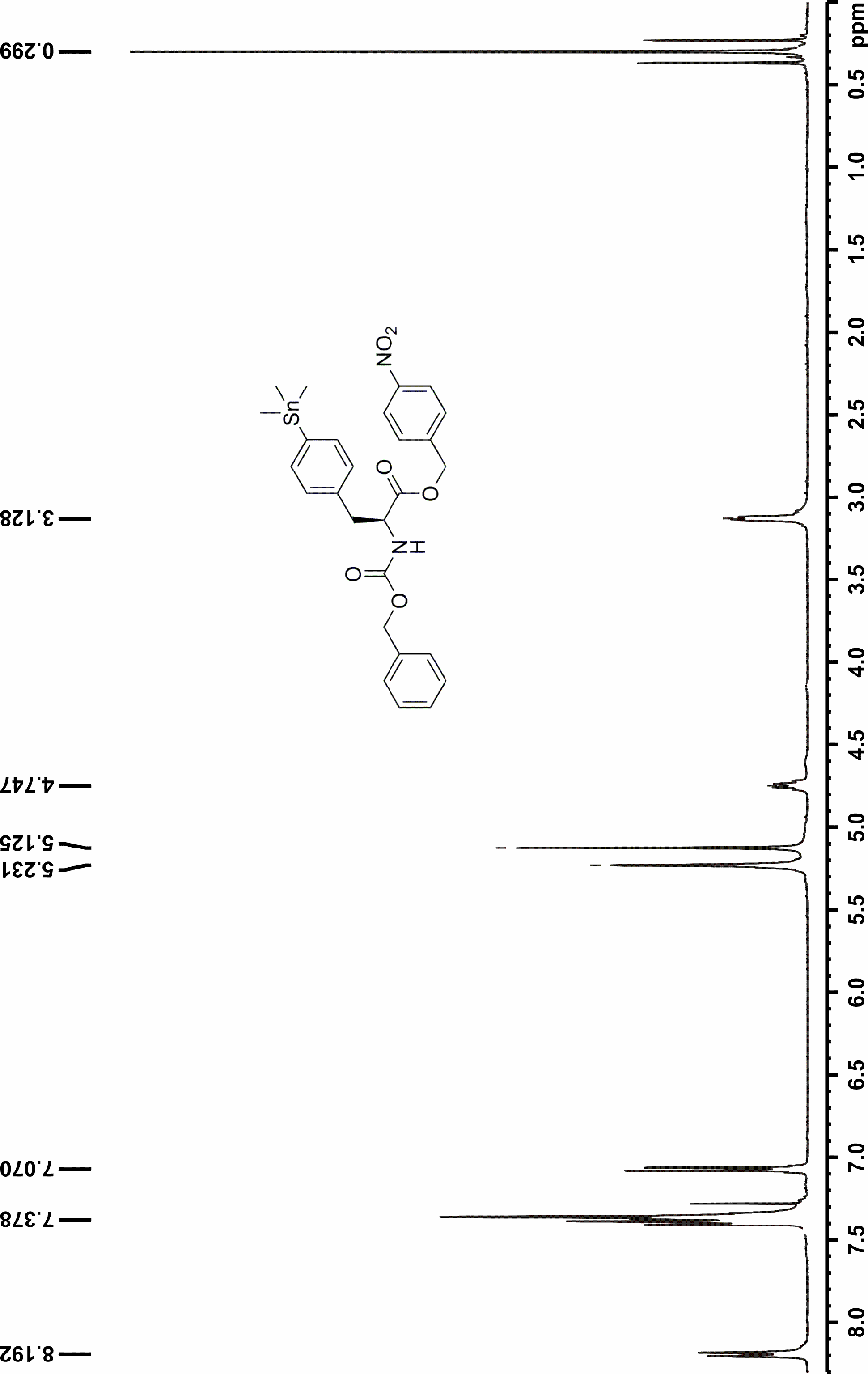


**Tetraprotected bis-amino acid α-oxodiphenylmethane (3a)**

^1^H NMR, 300 MHz, CDCl_3_


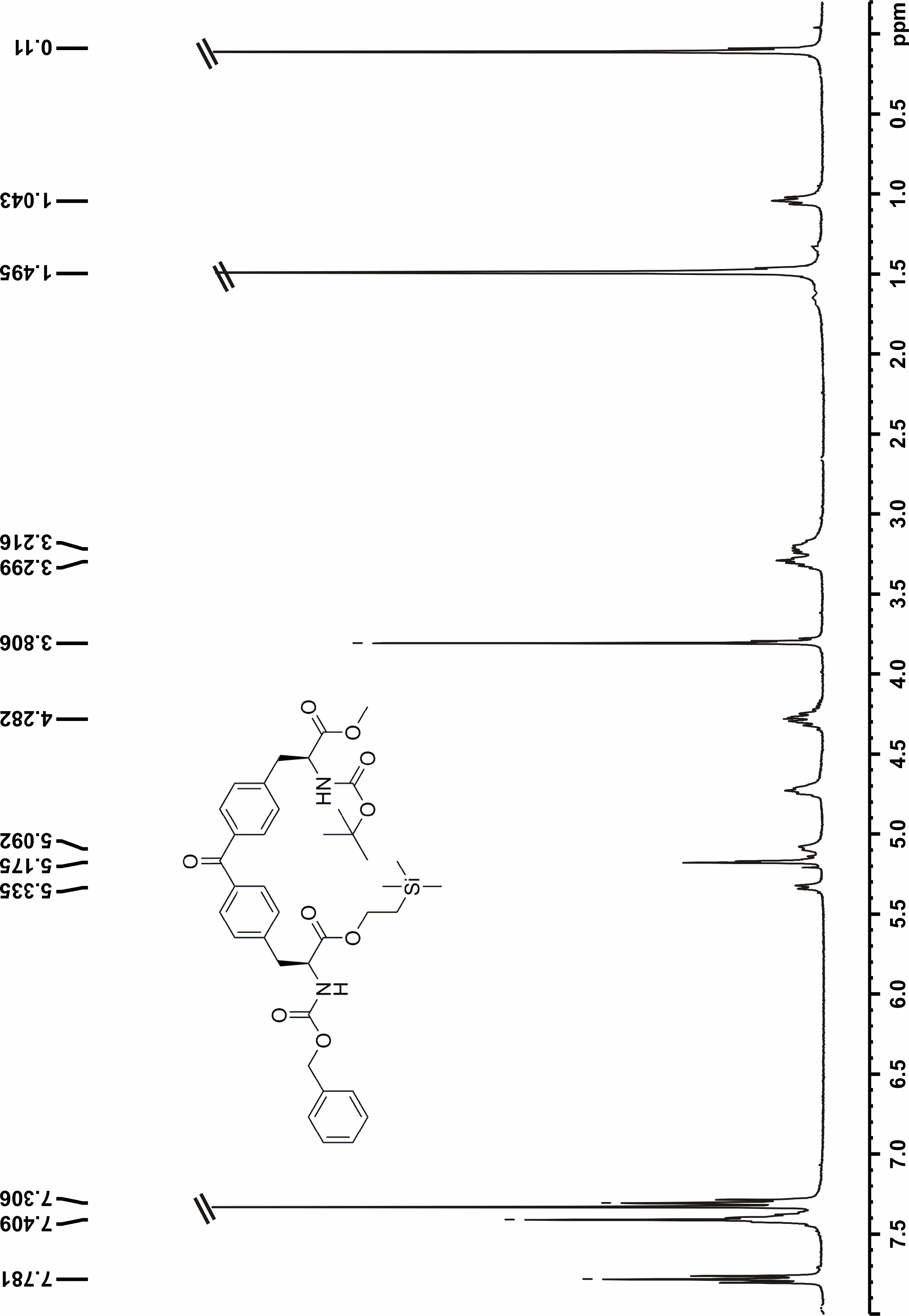


^13^C NMR, 100 MHz, CDCl_3_


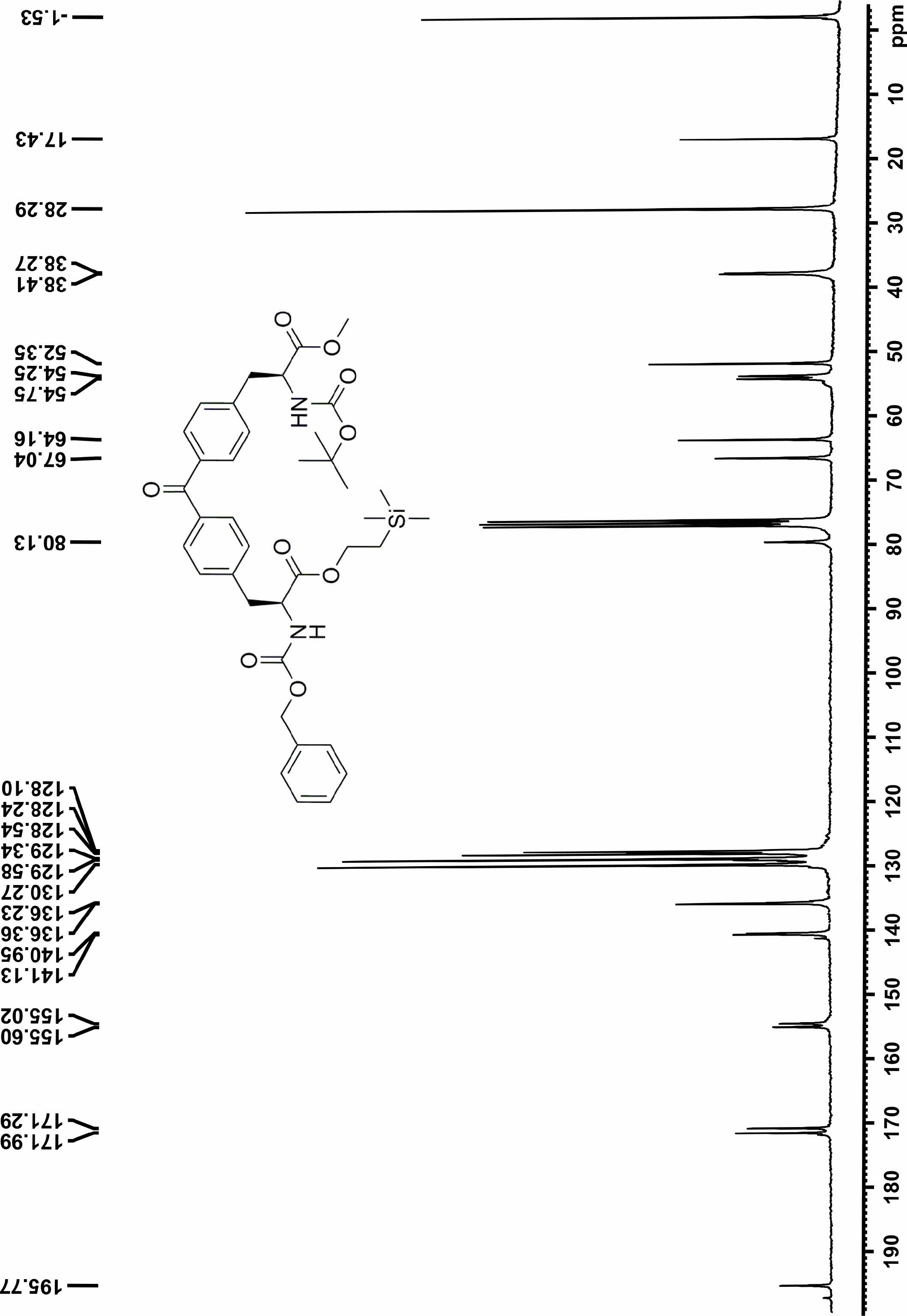


**Tetraprotected bis-amino acid α-oxodiphenylmethane (3b)**

^1^H NMR, 400 MHz, CDCl_3_


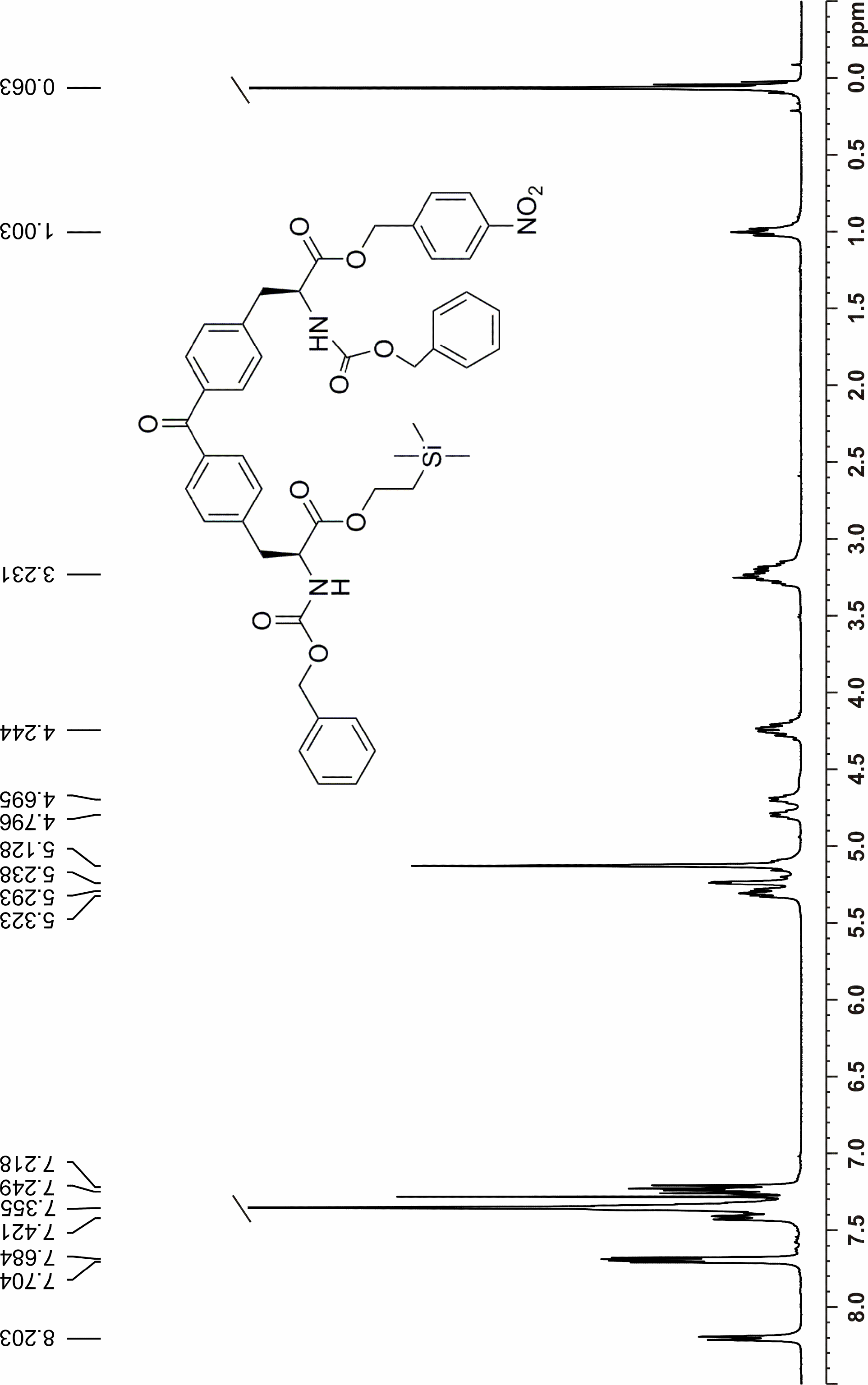


^13^C NMR, 100 MHz, CDCl_3_


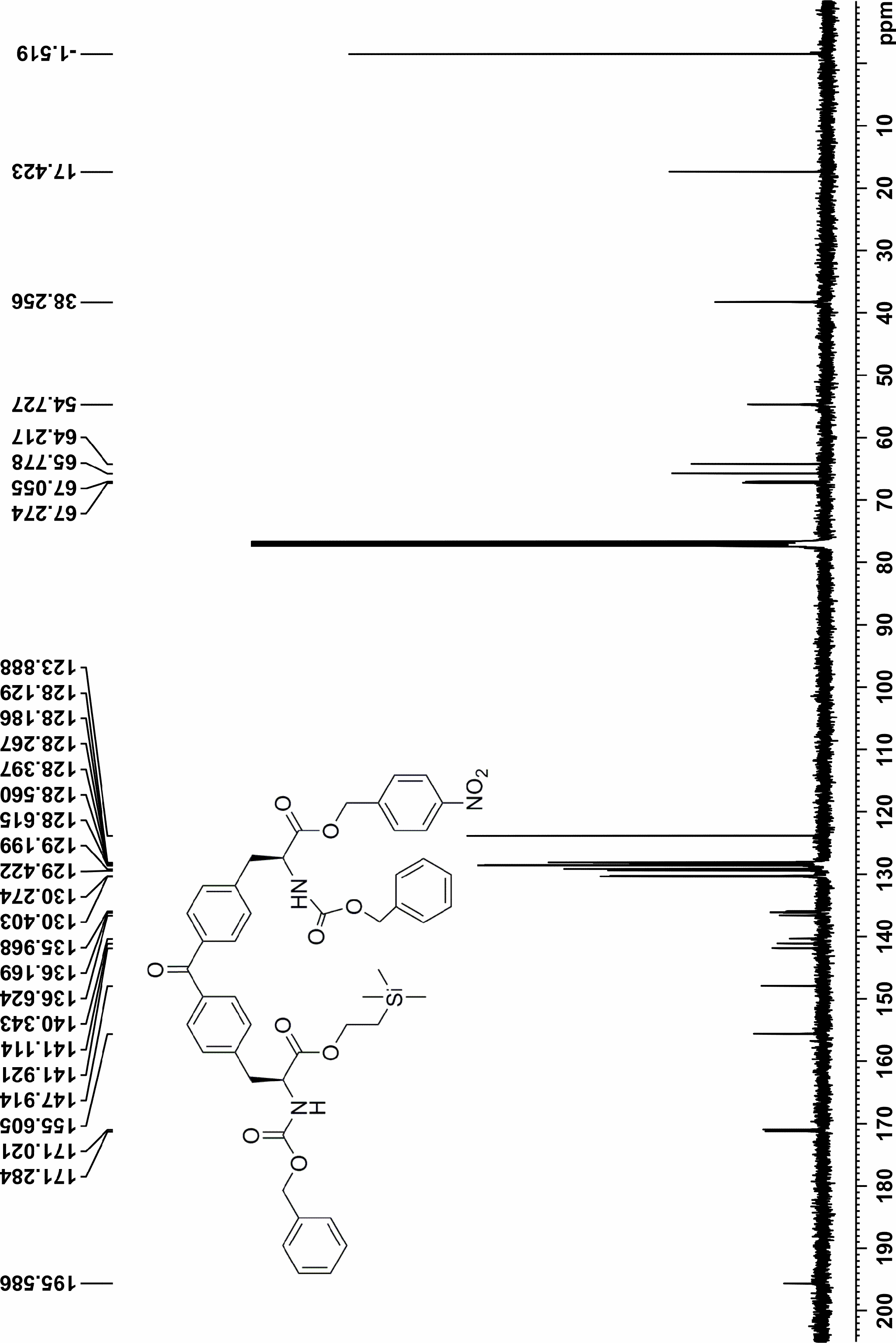


**Tetraprotected bis-amino acid α-oxodiphenylmethane (3c)**

^1^H NMR, 400 MHz, CDCl_3_


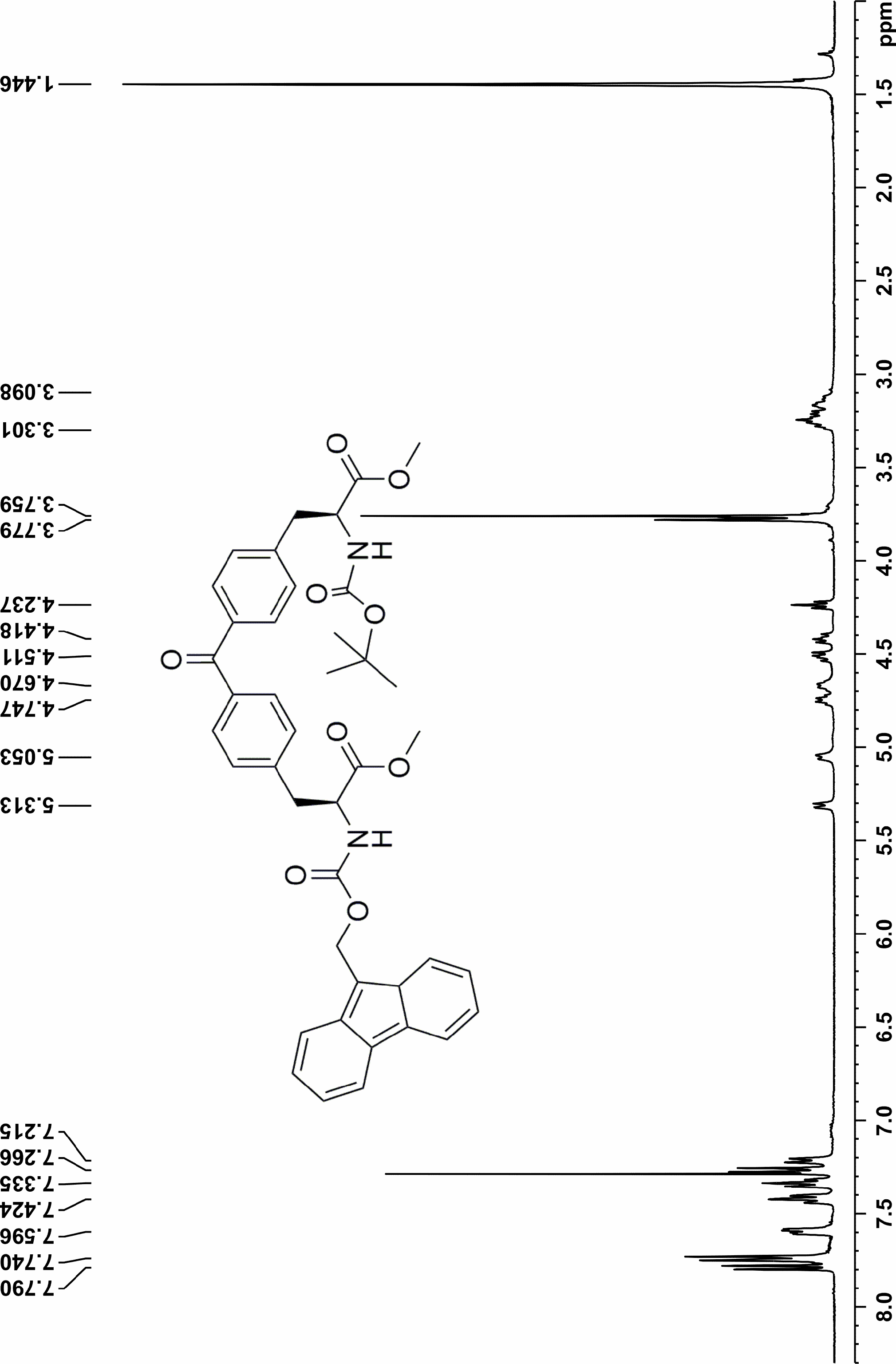


^13^C NMR, 100 MHz, CDCl_3_


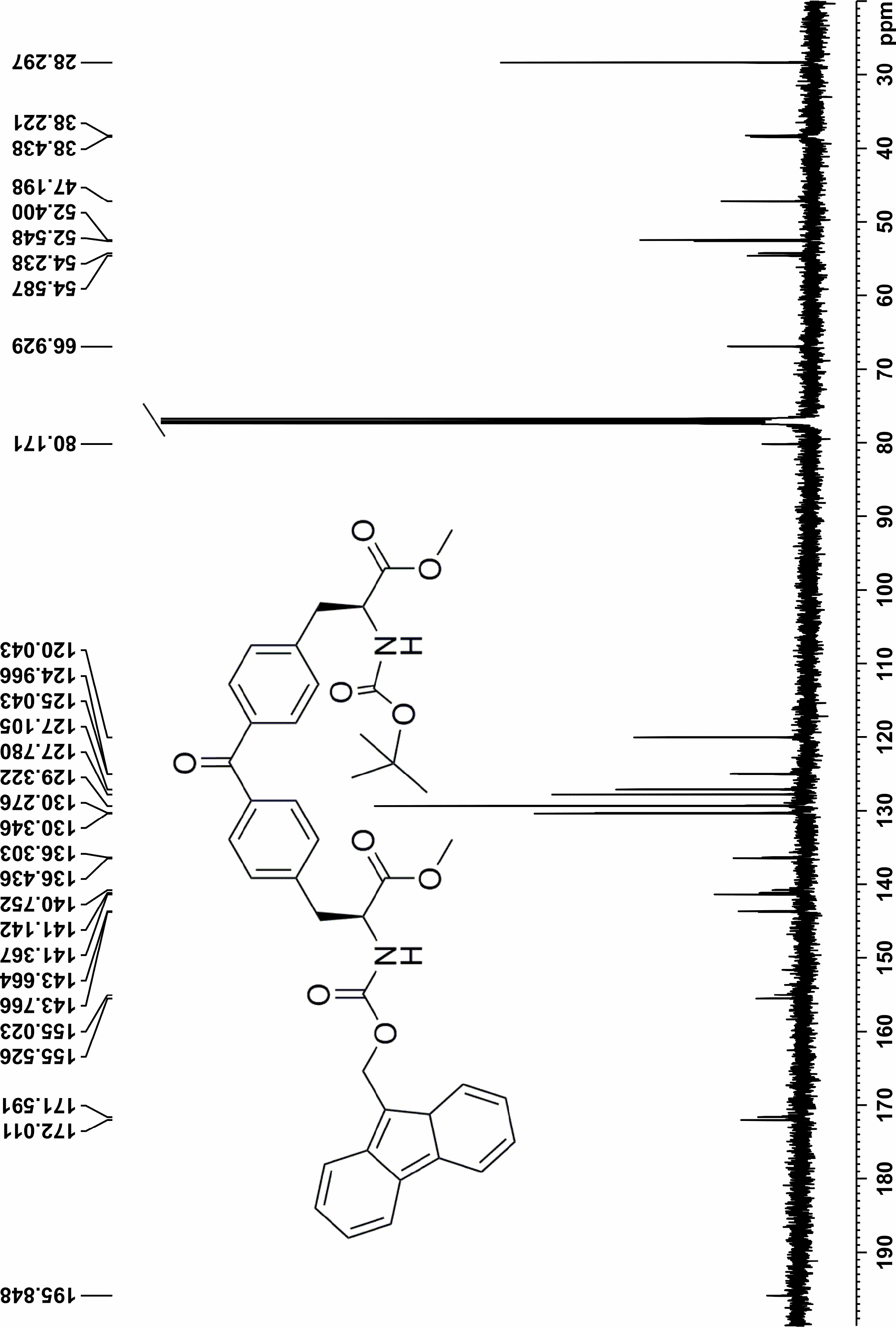


**Compound 8**

^1^H NMR, 300 MHz, CDCl_3_


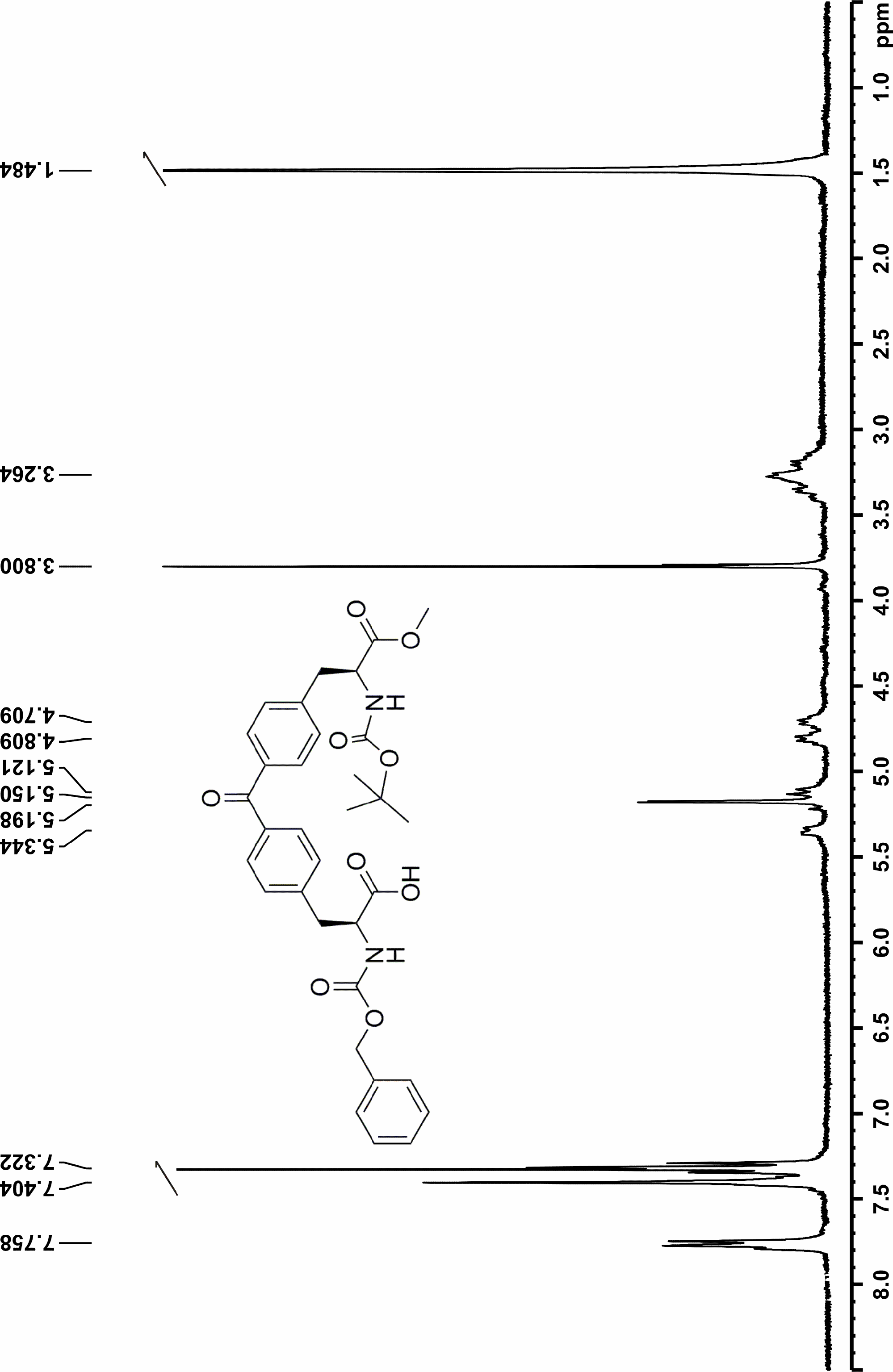


^13^C NMR, 100 MHz, CDCl_3_


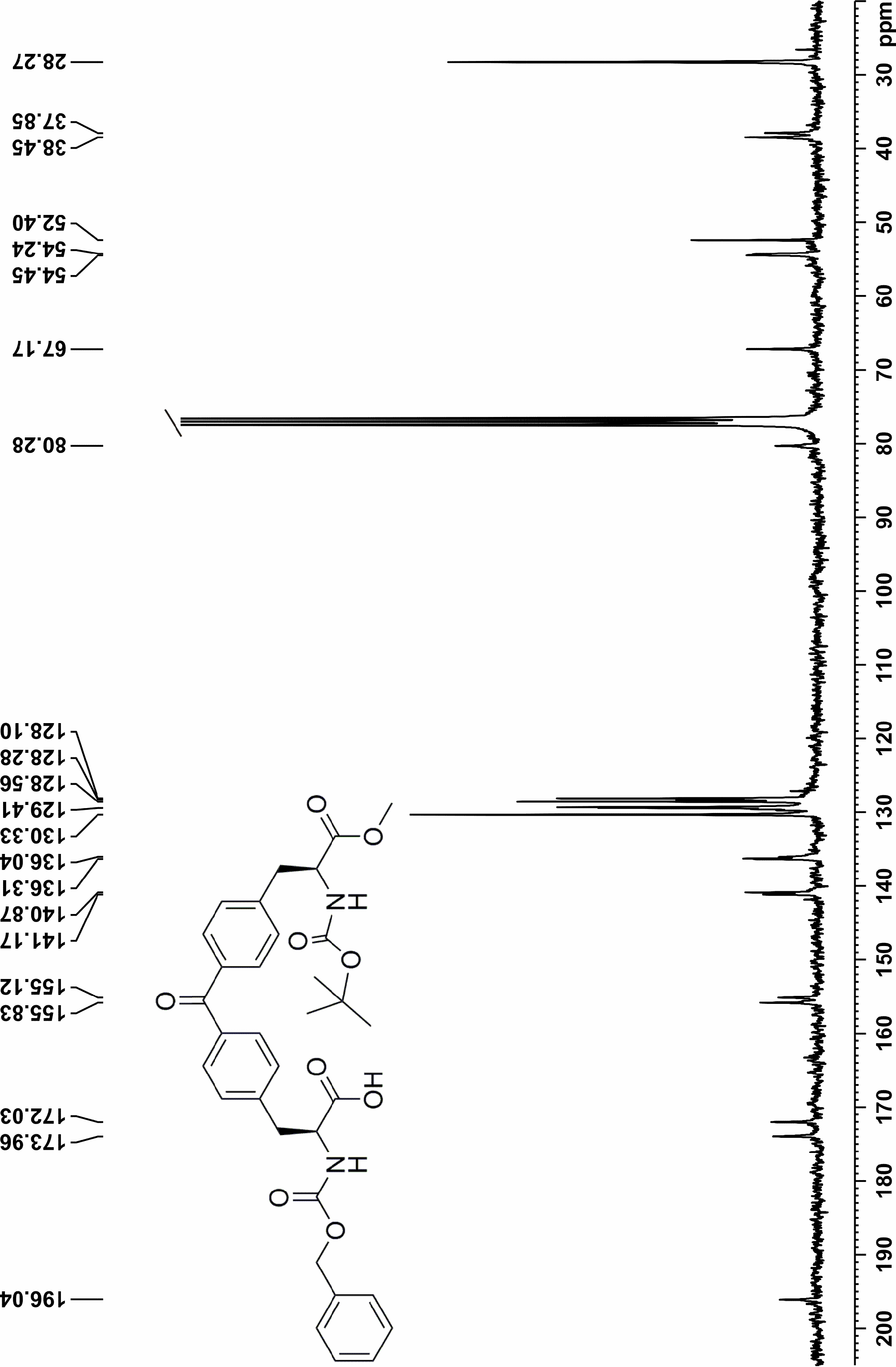


**Compound 9**

^1^H NMR, 300 MHz, CDCl_3_


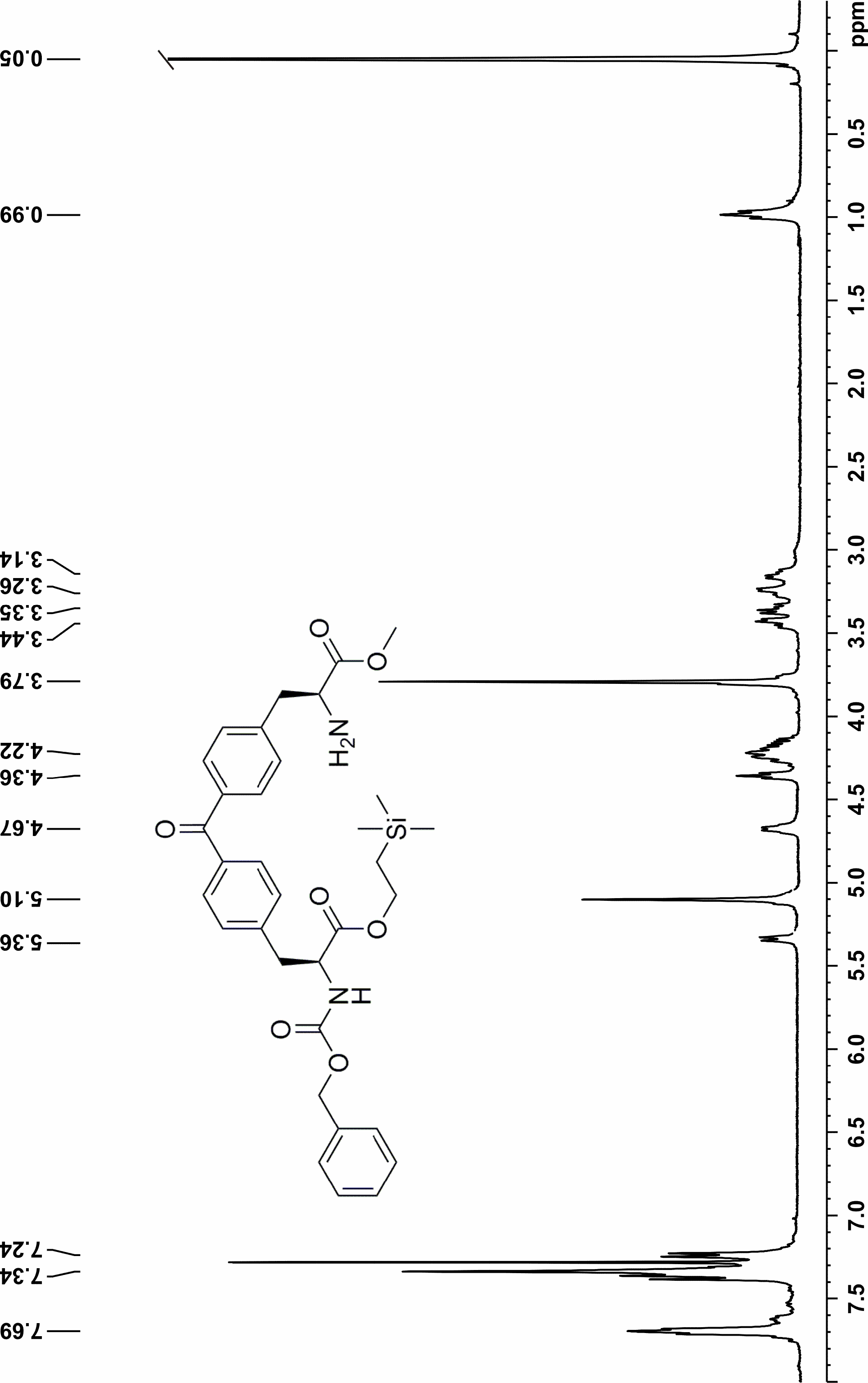


^13^C NMR, 100 MHz, CDCl_3_


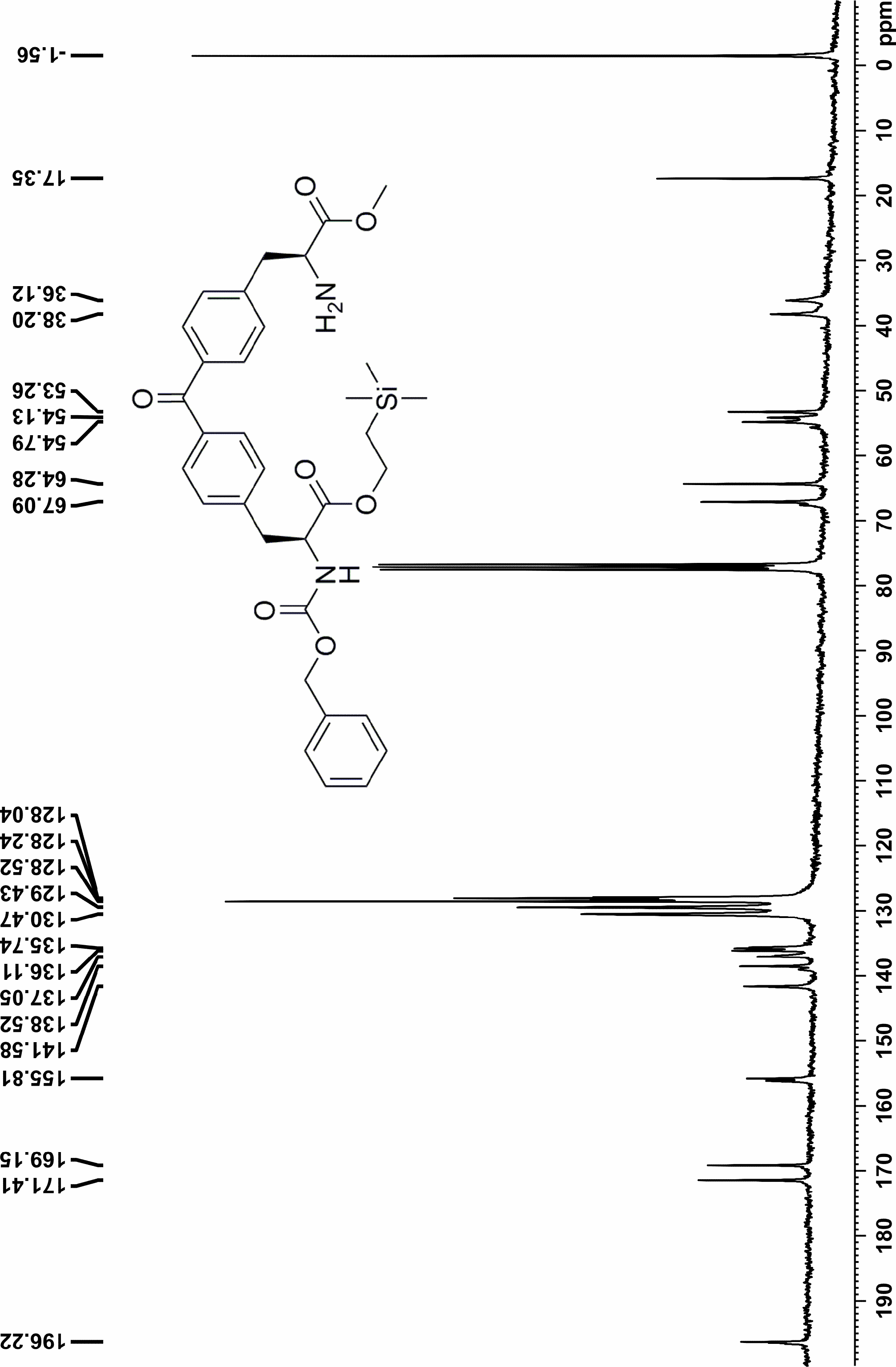


**Compound 10**

^1^H NMR, 400 MHz, CDCl_3_


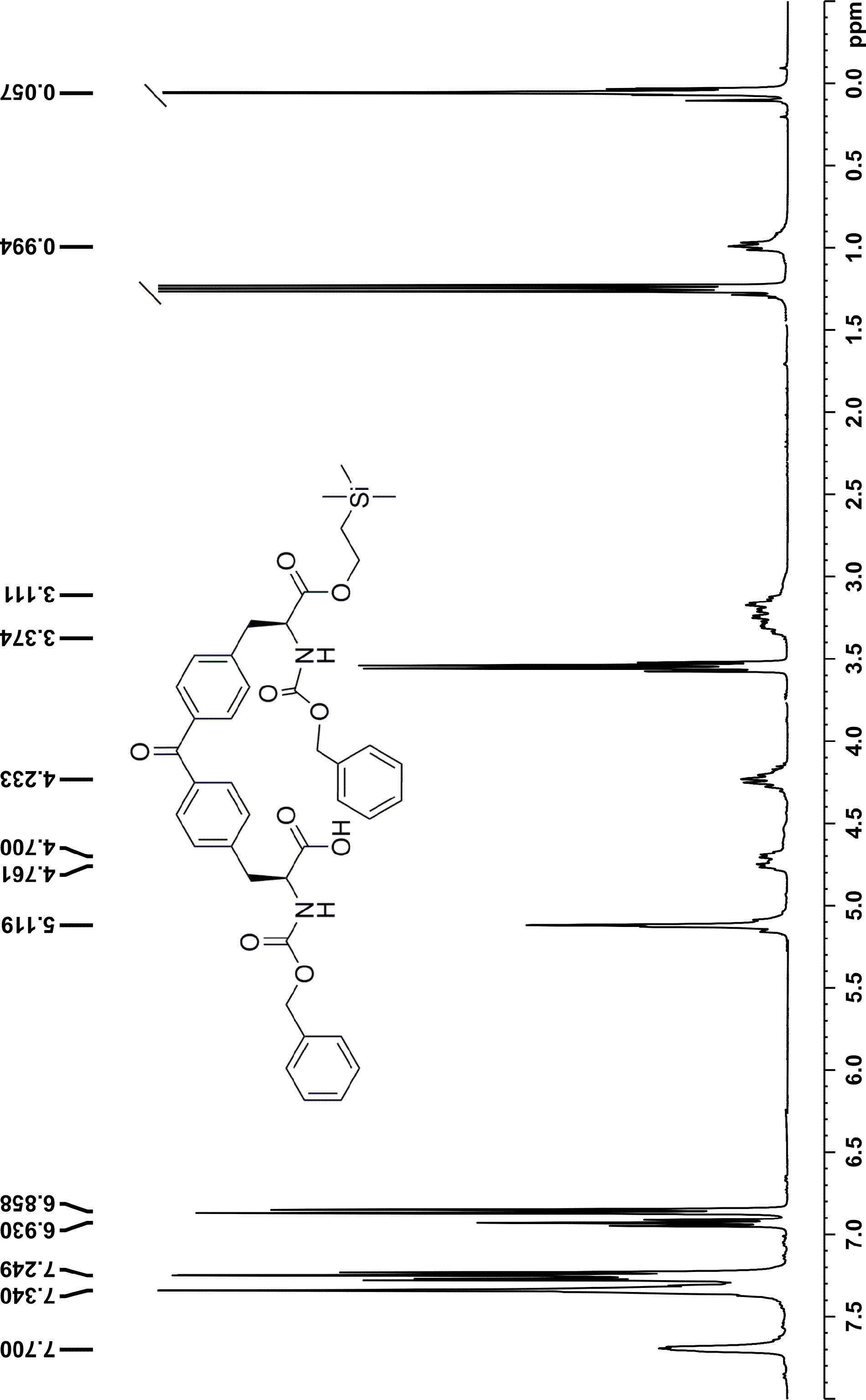


^13^C NMR, 100 MHz, CDCl_3_


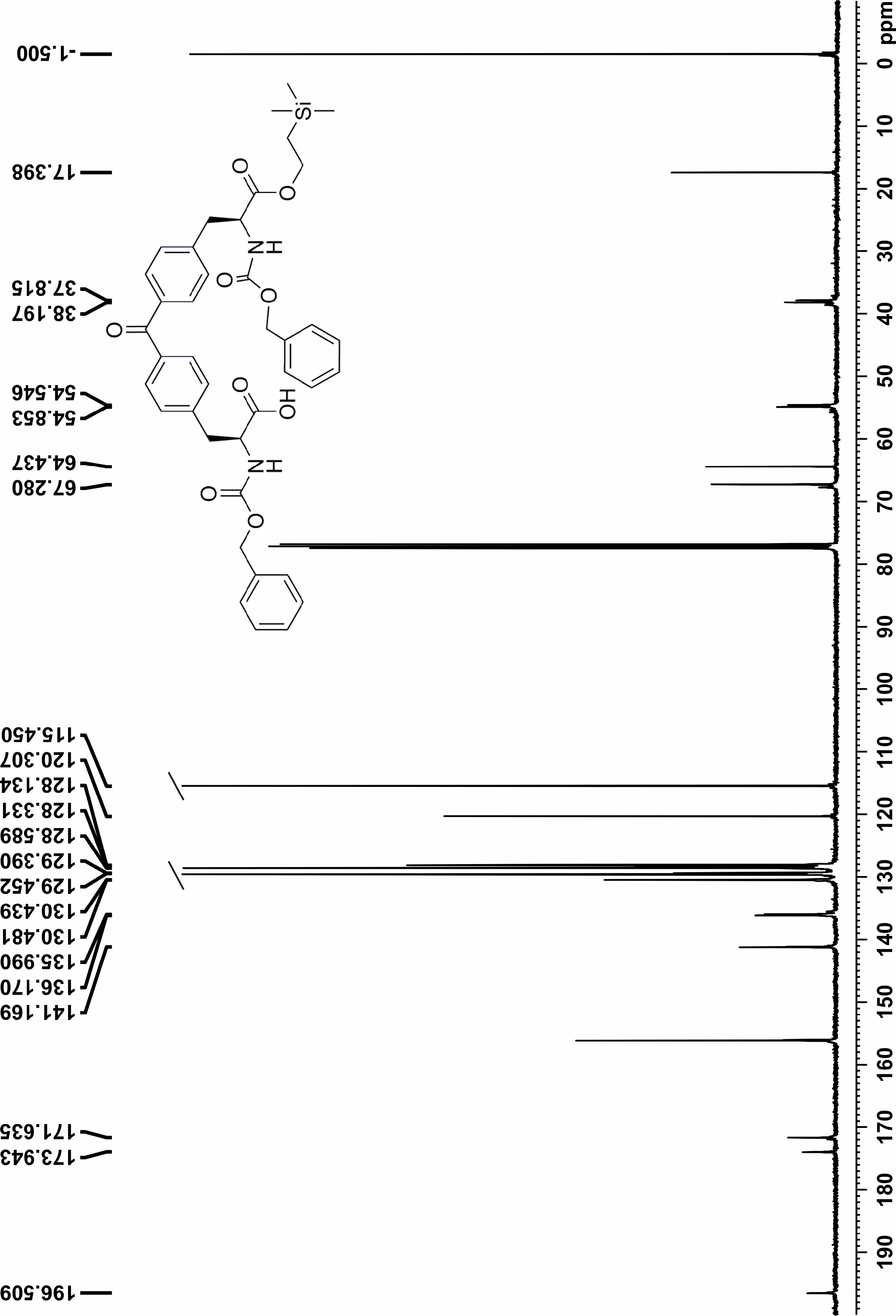


**Compound 11**

^1^H NMR, 400 MHz, CDCl_3_


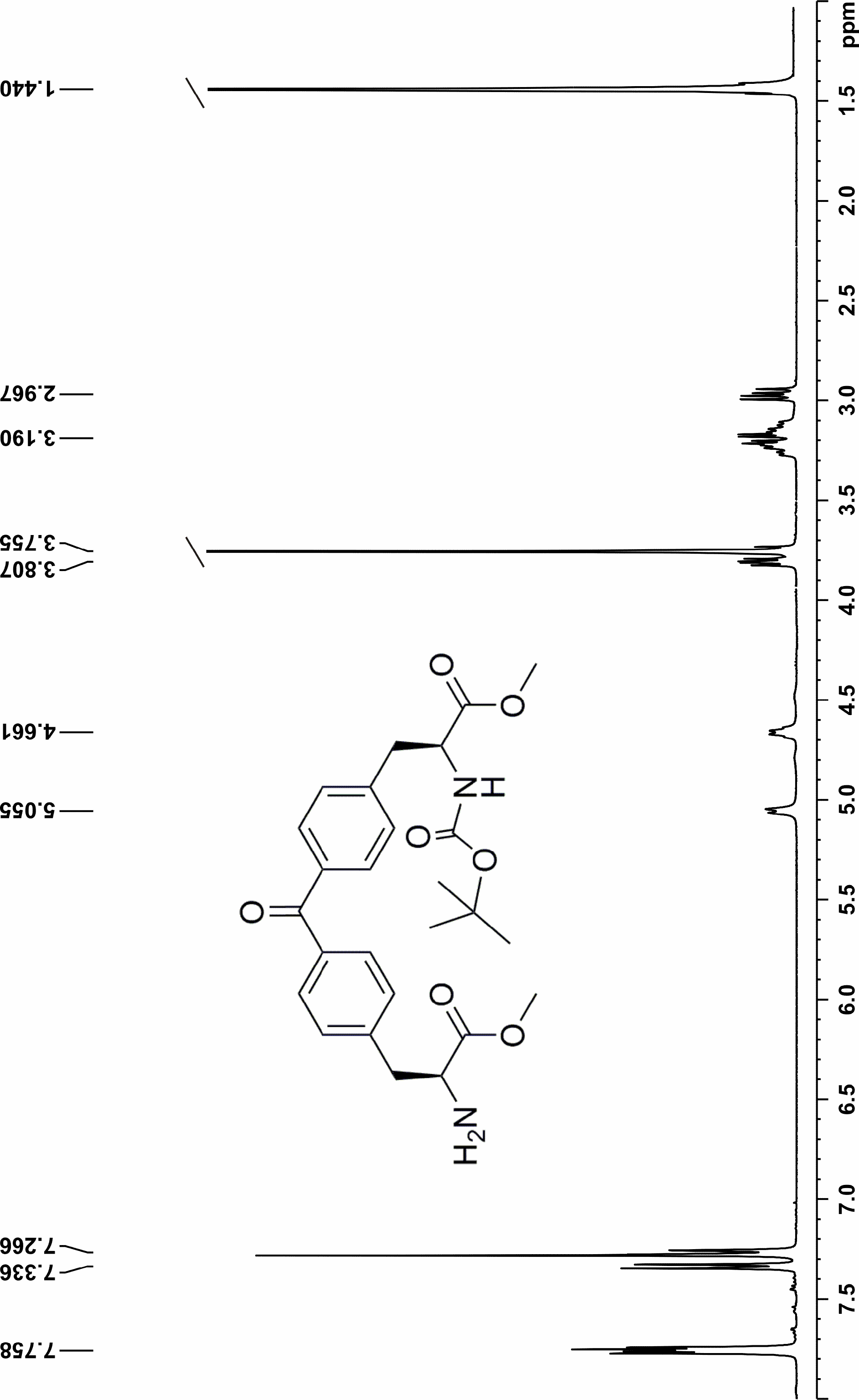


**Tetraprotected Bis-Dipeptide all-*S*-12**

^1^H NMR, 400 MHz, CDCl_3_


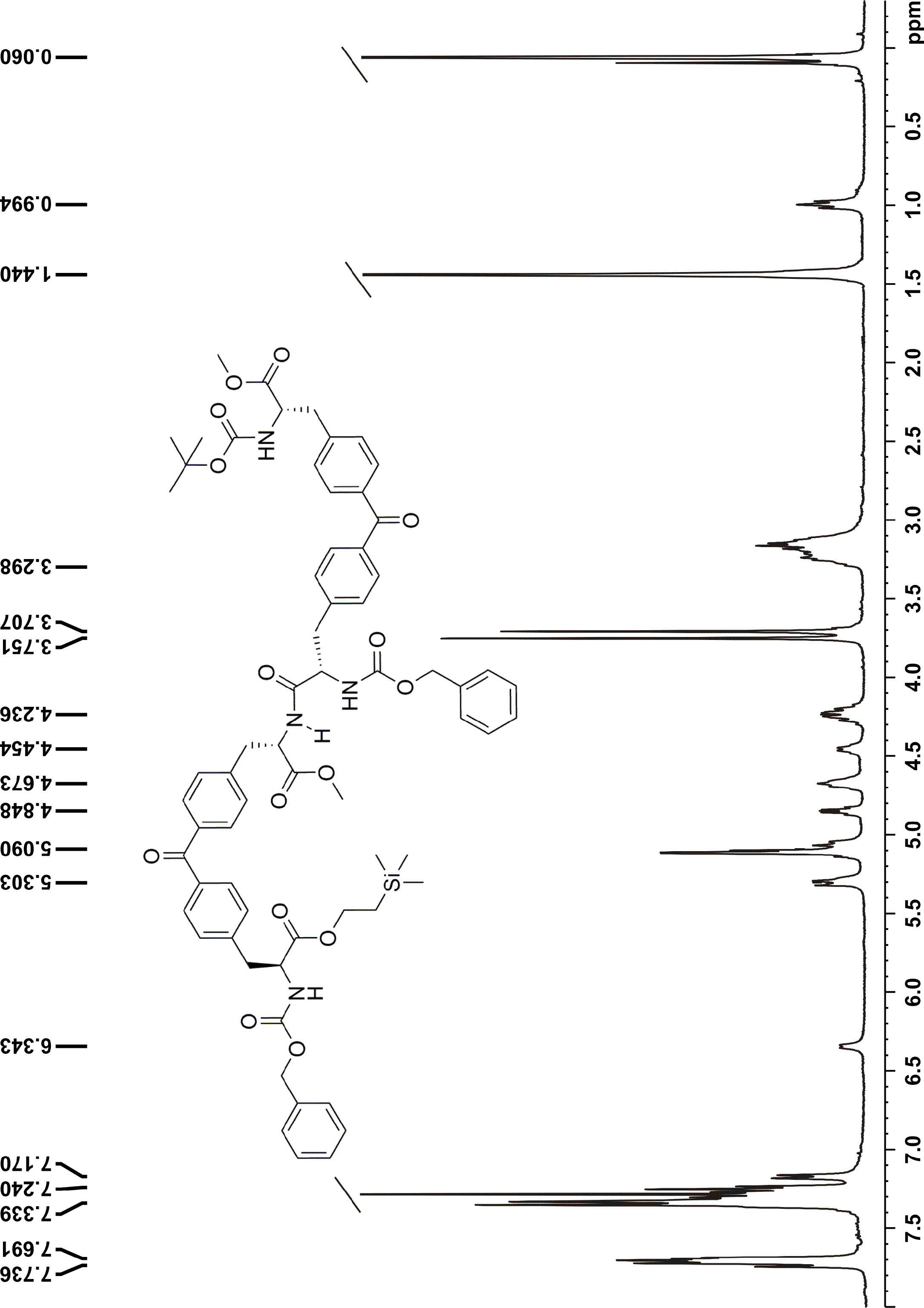


^13^C NMR, 100 MHz, CDCl_3_


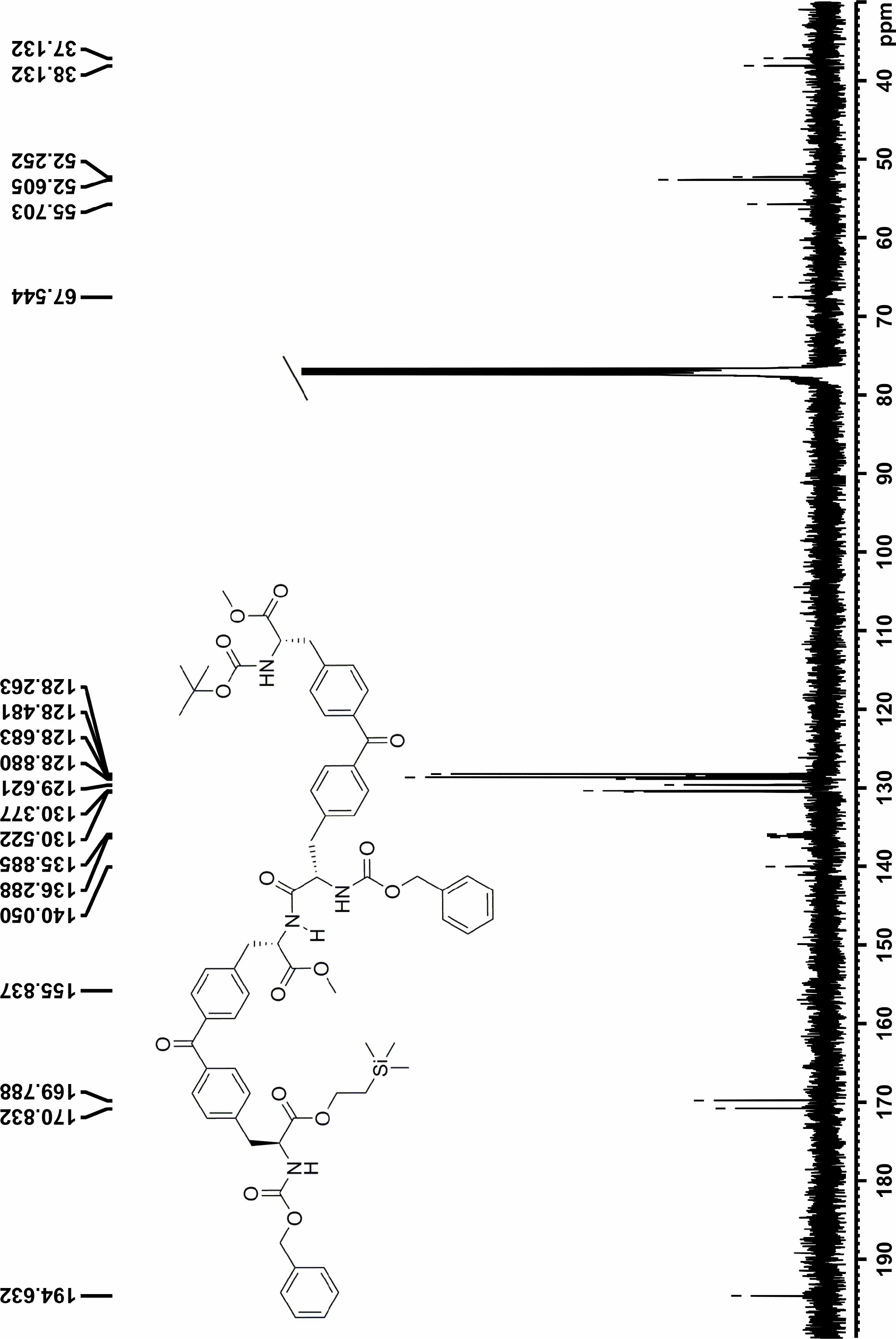


**Tetraprotected Bis-Dipeptide all-*S*-13**

^1^H NMR, 400 MHz, CDCl_3_


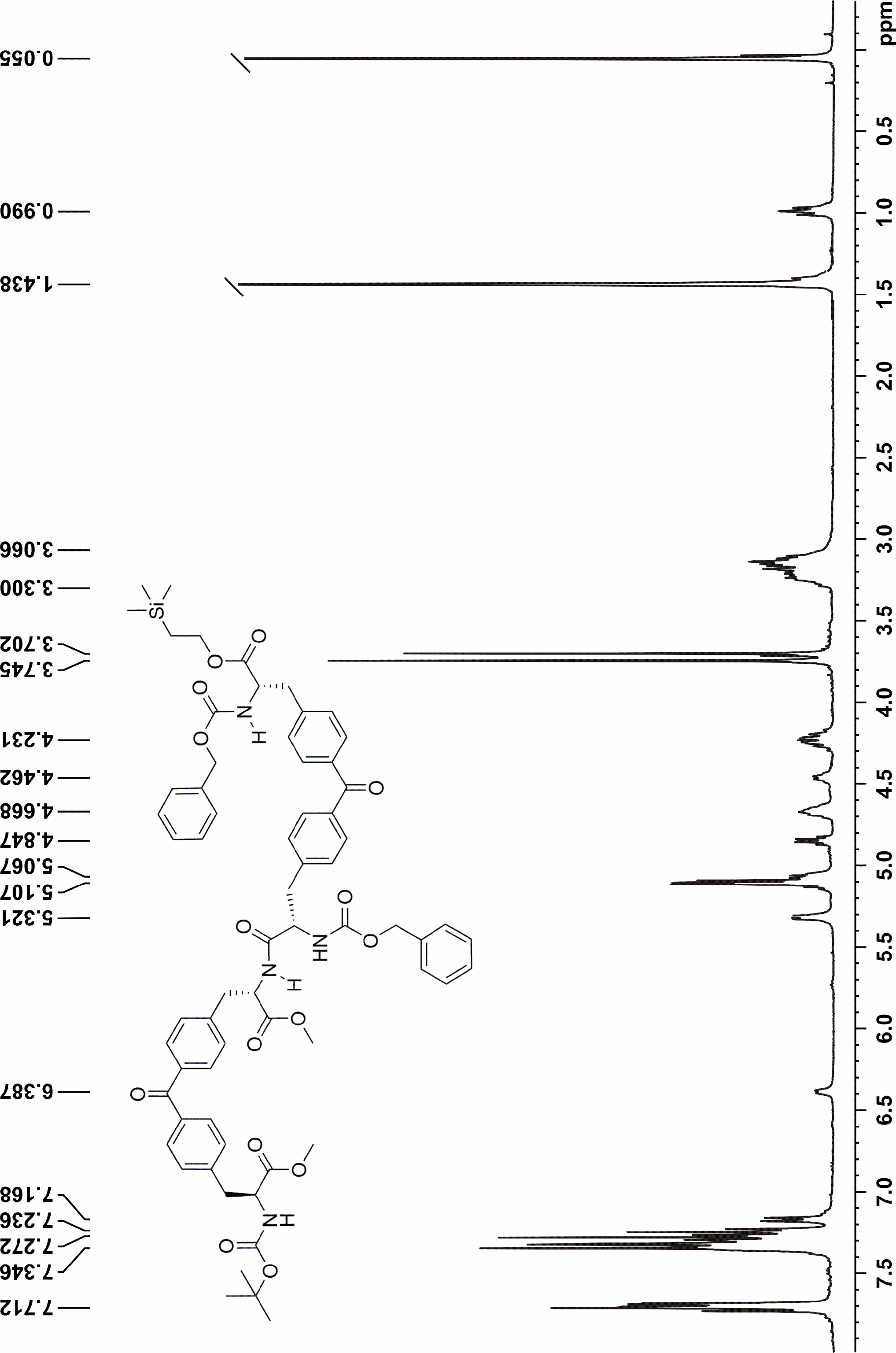


^13^C NMR, 100 MHz, CDCl_3_


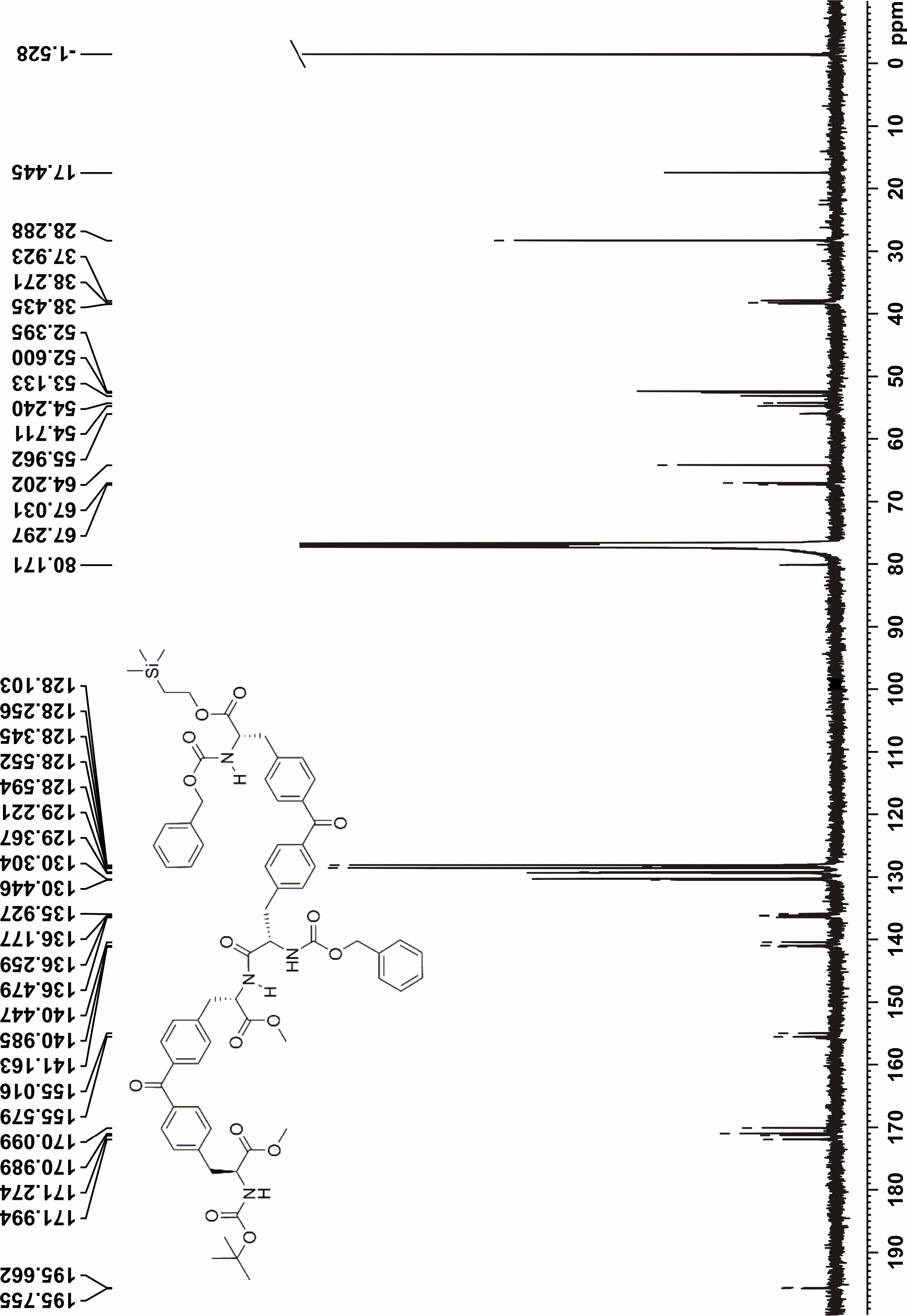


**Acid 14**

^1^H NMR, 300 MHz, CDCl_3_


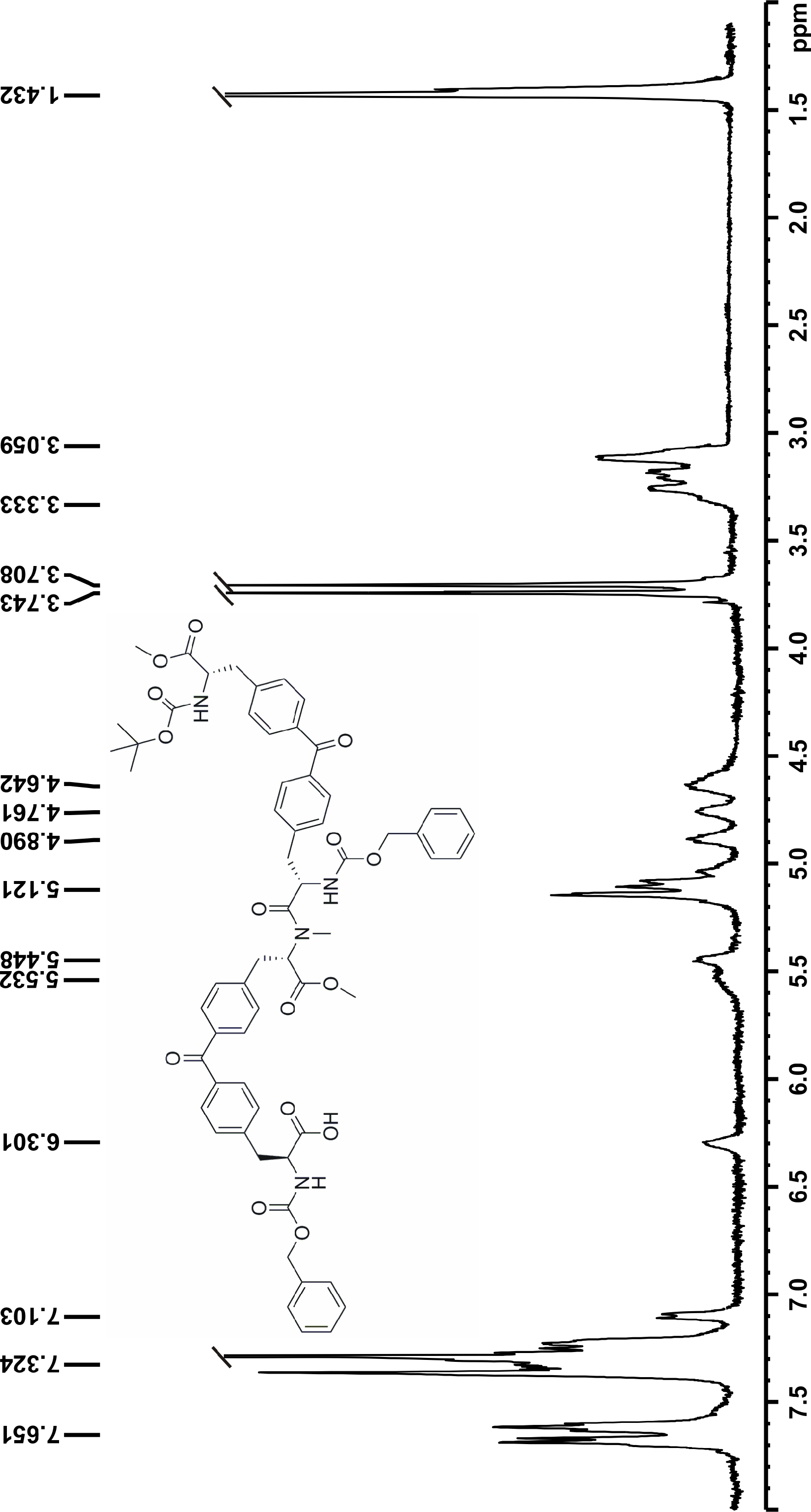


^13^C NMR, 100 MHz, CDCl_3_


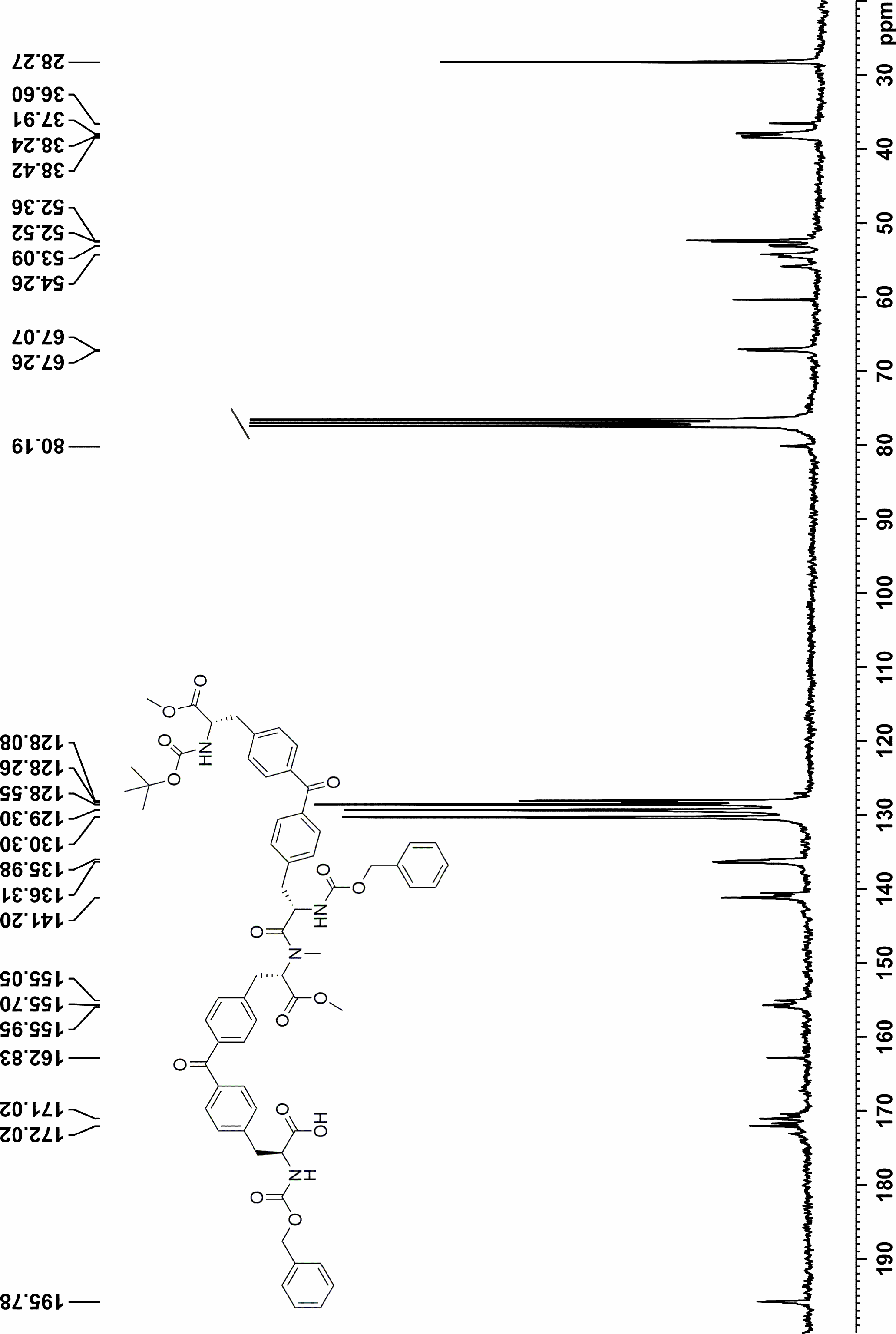


**Acid 16**

^1^H NMR, 400 MHz, CDCl_3_


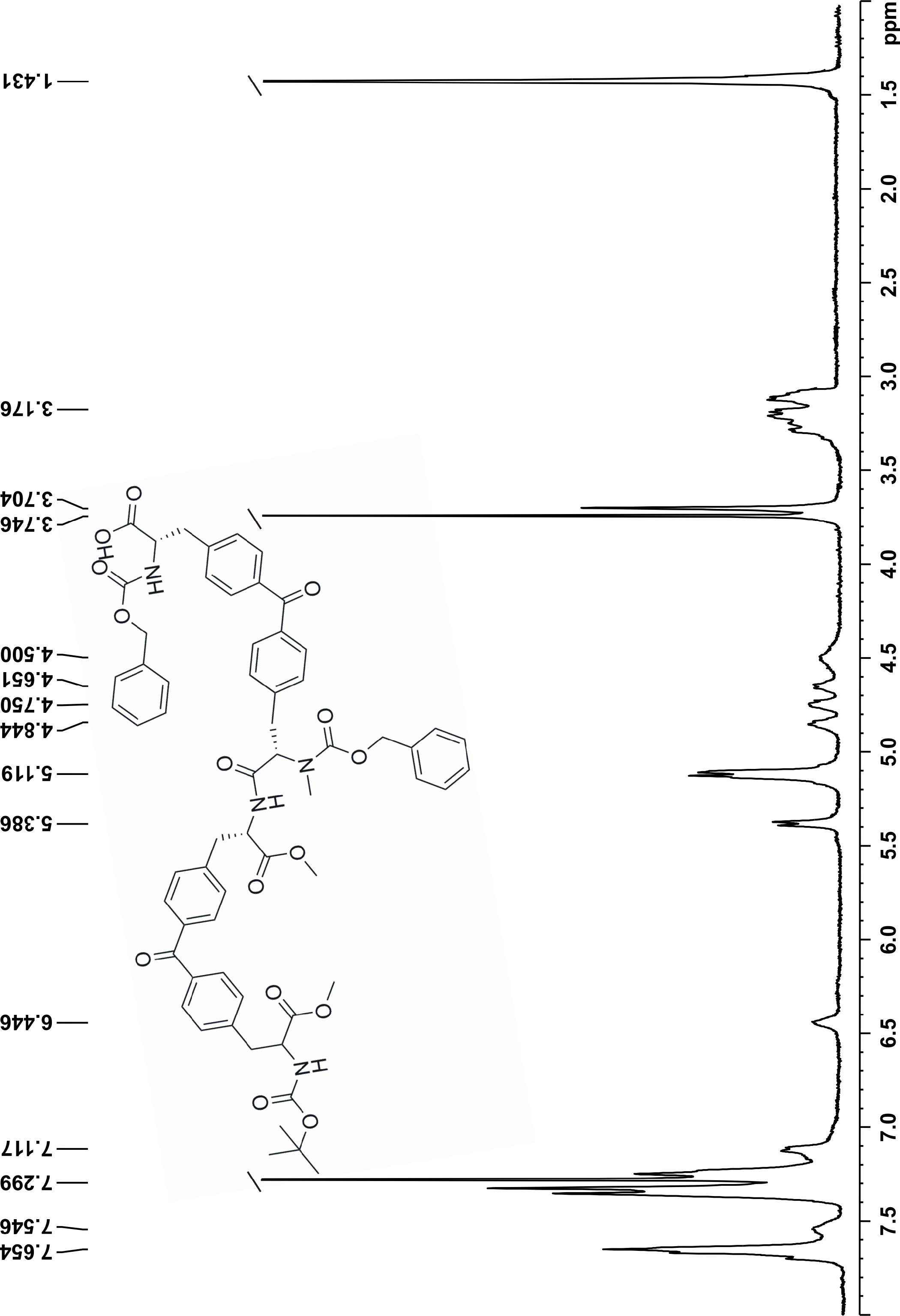


**Amino acid 15**

^1^H NMR, 300 MHz, CDCl_3_


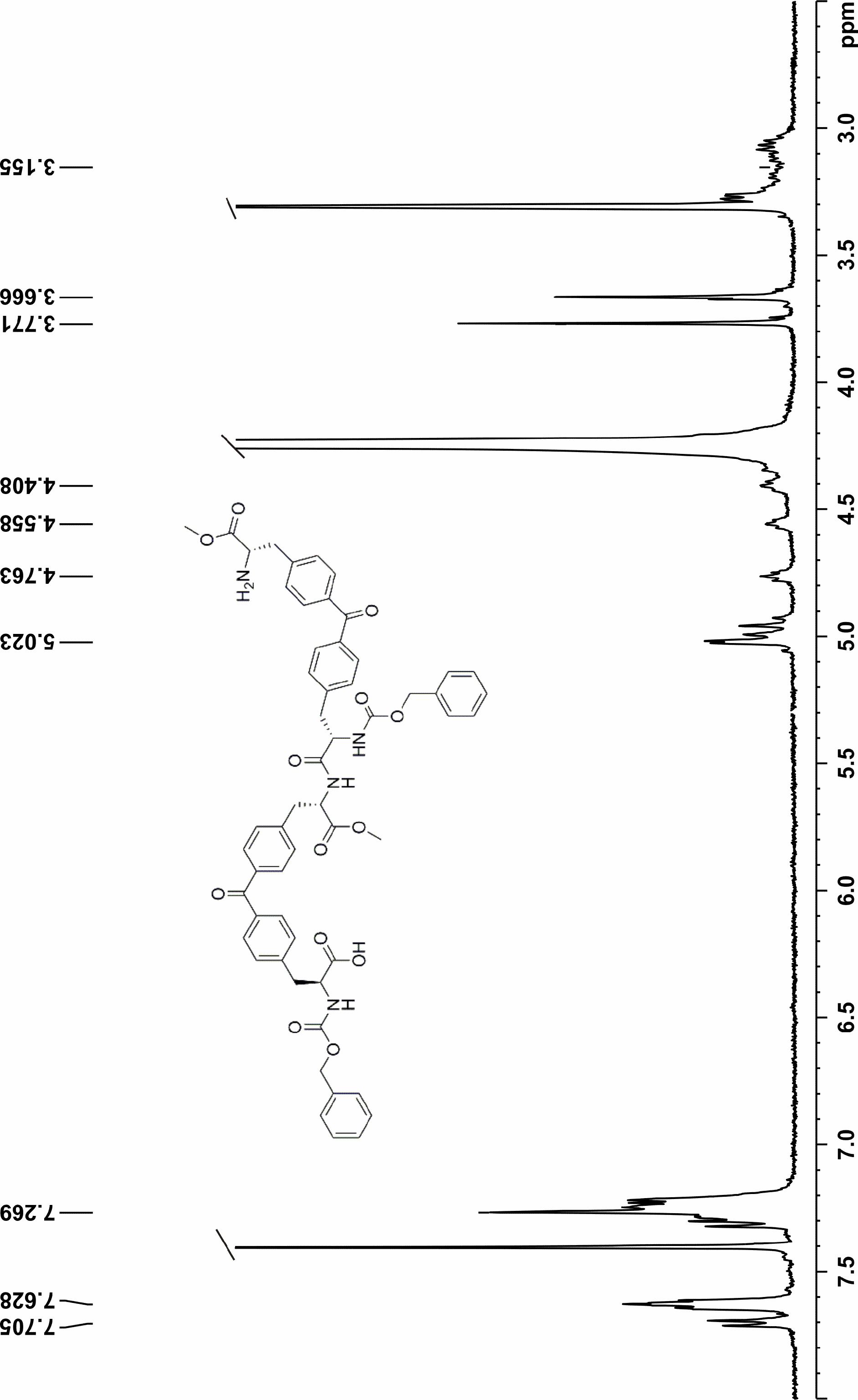


^13^C NMR, 100 MHz, CDCl_3_/MeOH


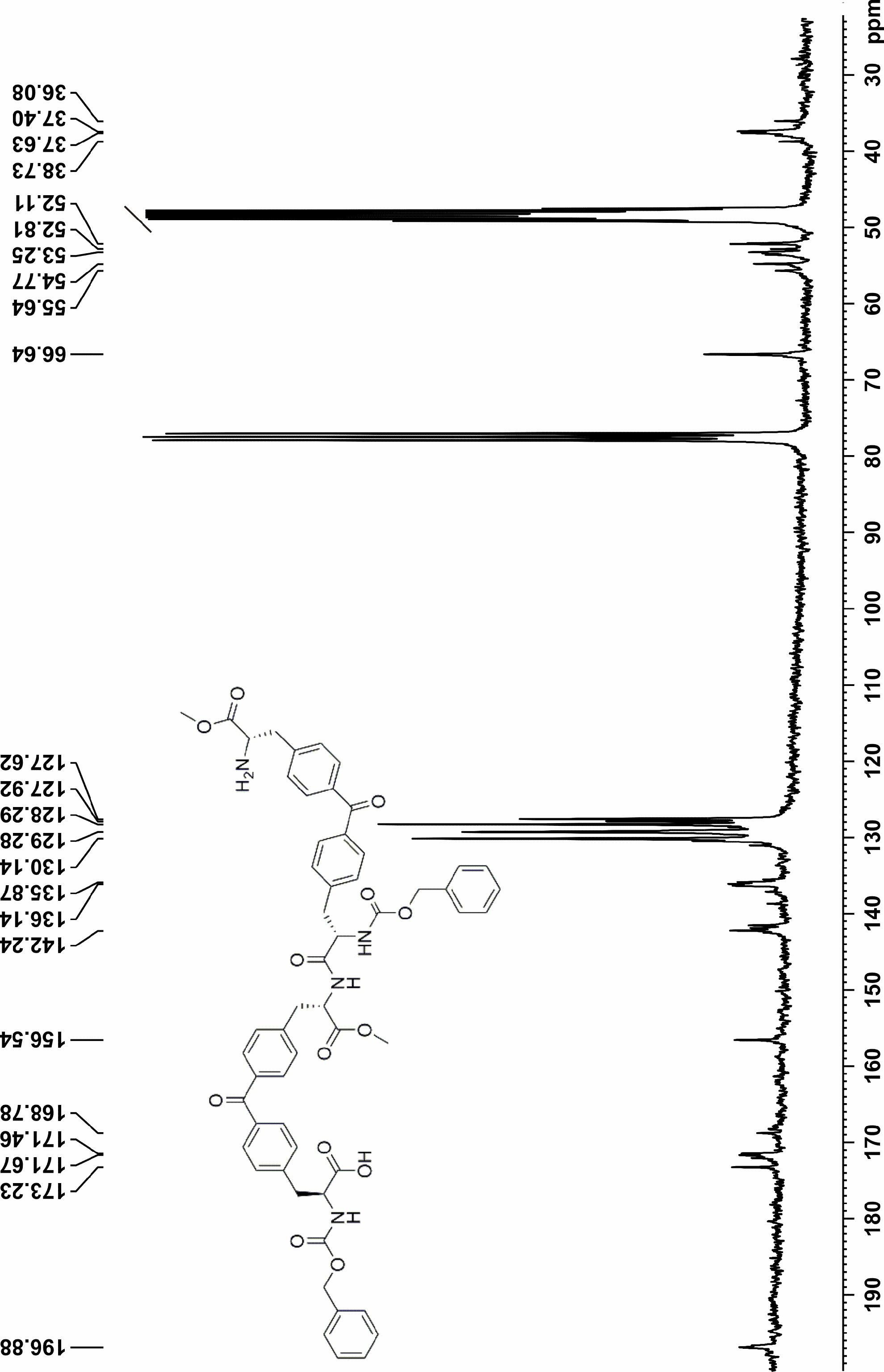


**Amino acid 17**

^1^H NMR, 400 MHz, CDCl_3_/MeOH


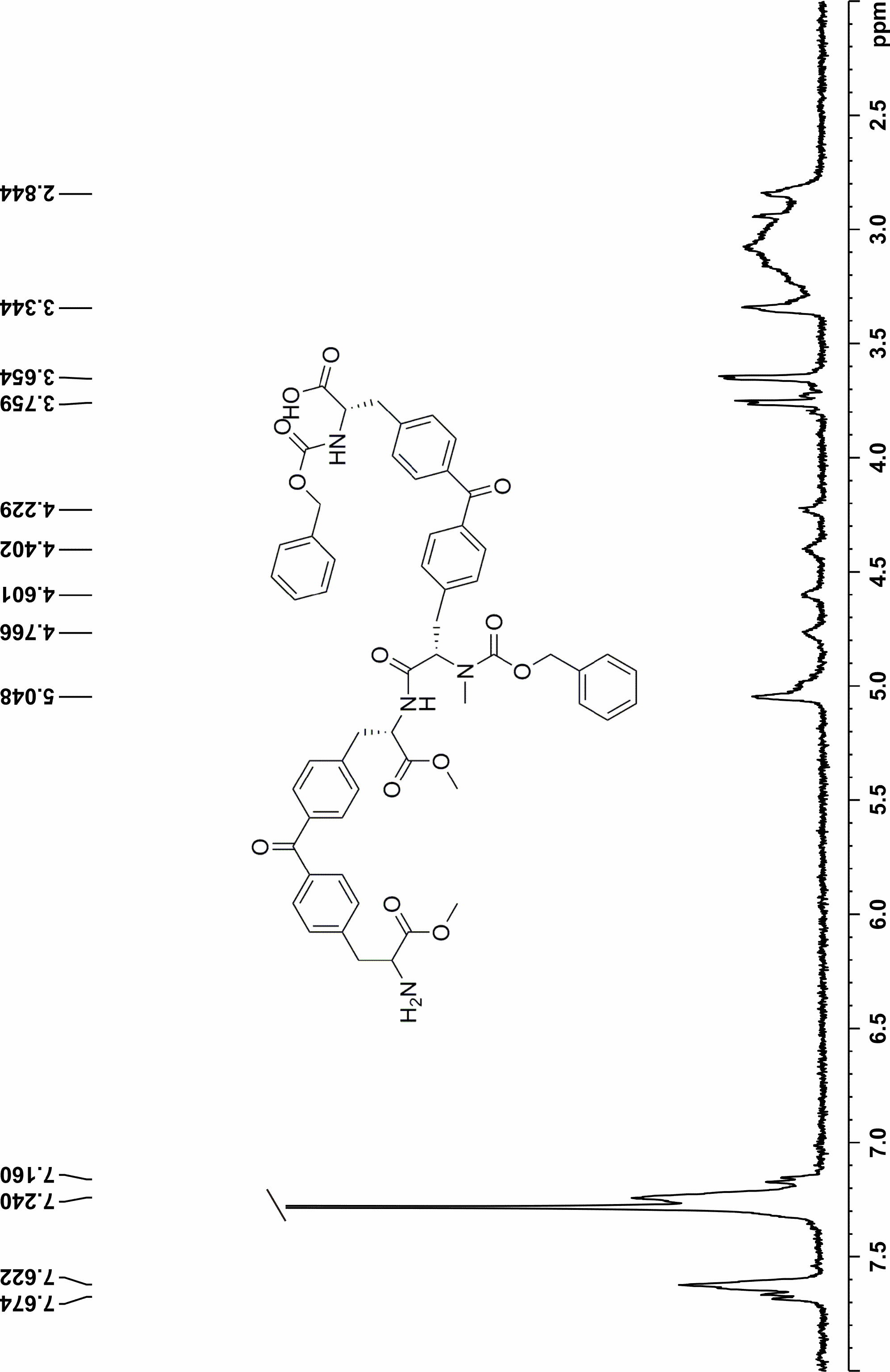


**Macrocyclic Bis-Dipeptide all-*S*-1**

^1^H NMR, 400 MHz, CDCl_3_


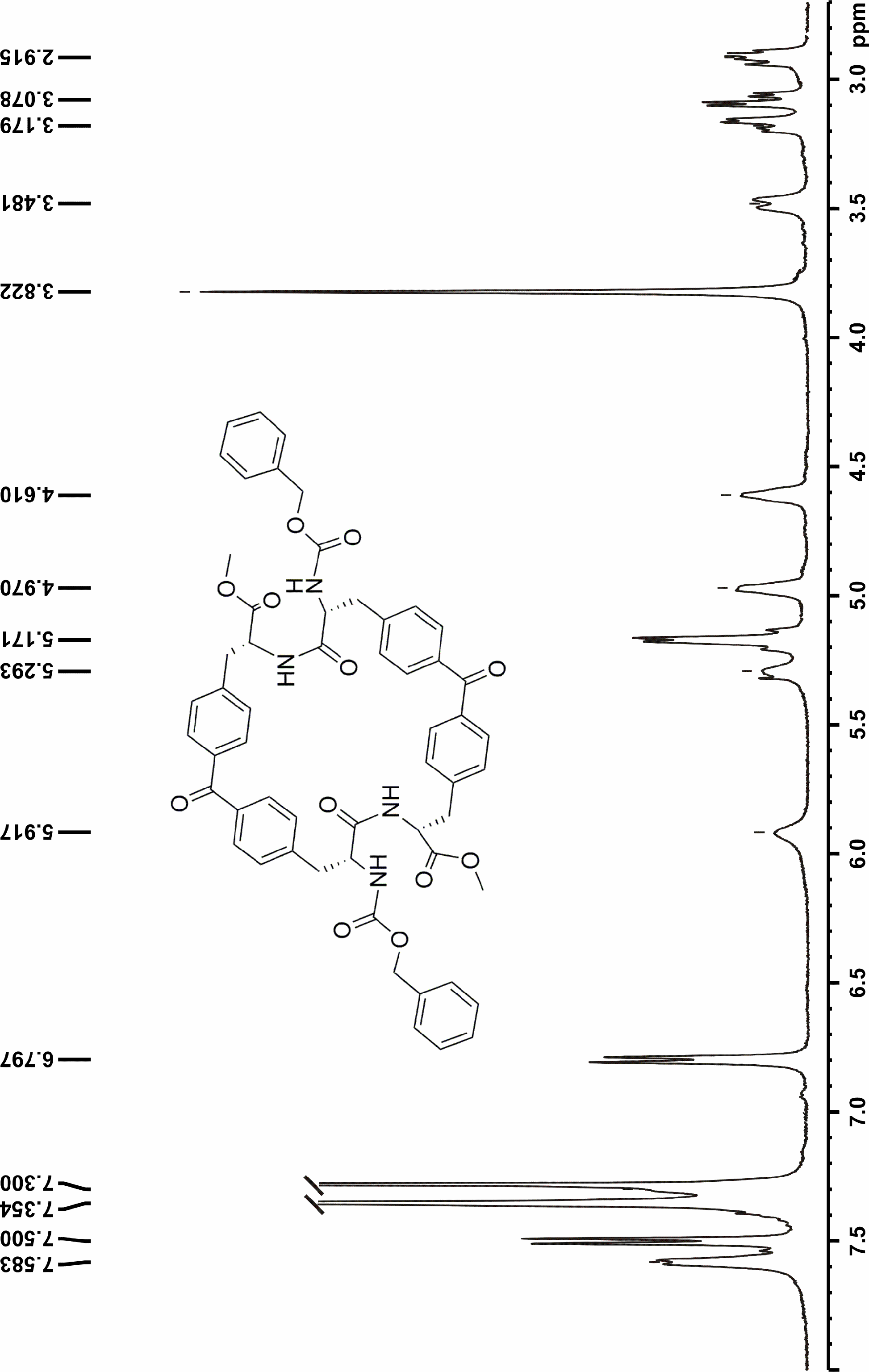


^13^C NMR, 100 MHz, CDCl_3_


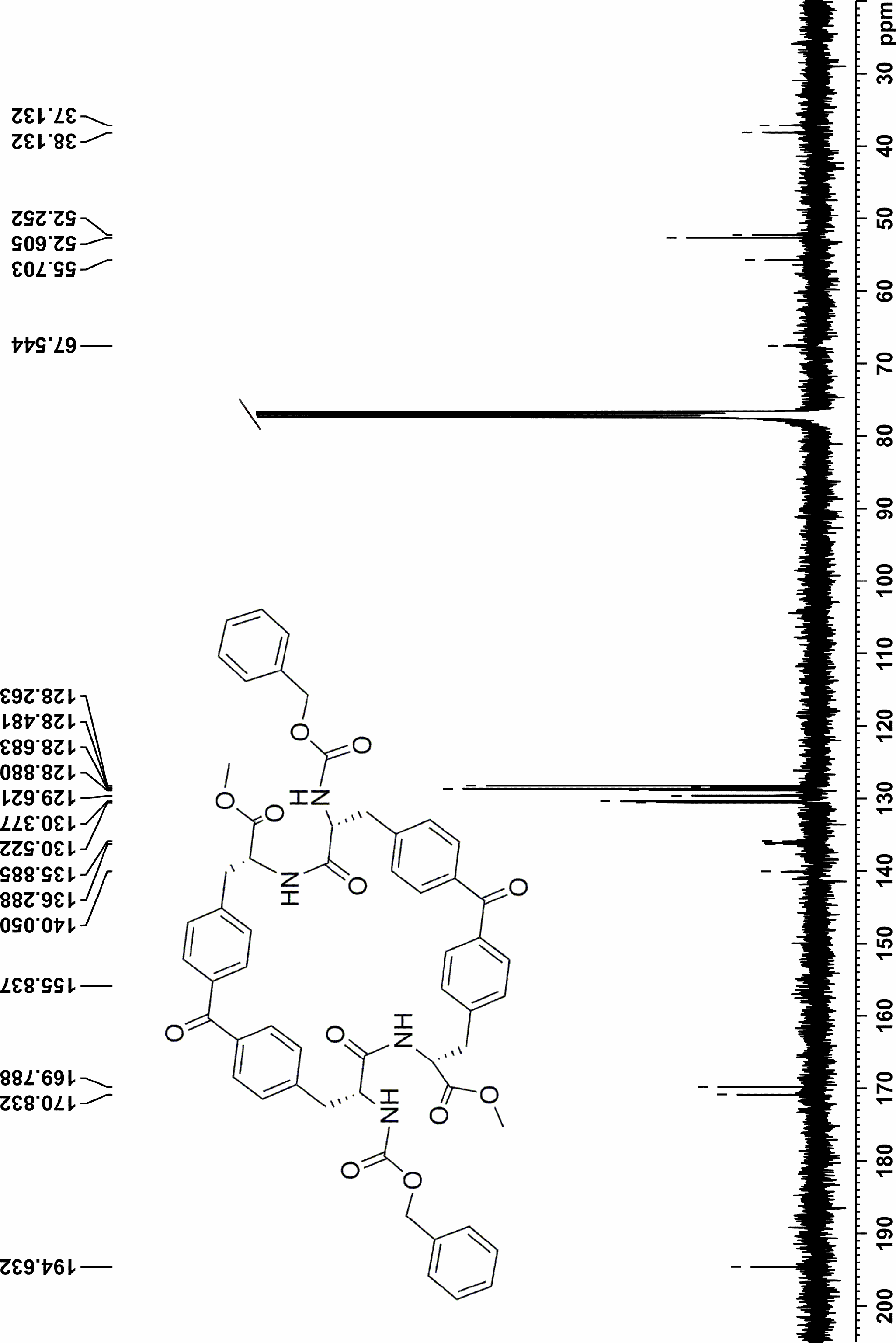


**Macrocyclic Bis-Dipeptide all-*S*-2**

^1^H NMR, 400 MHz, CDCl_3_


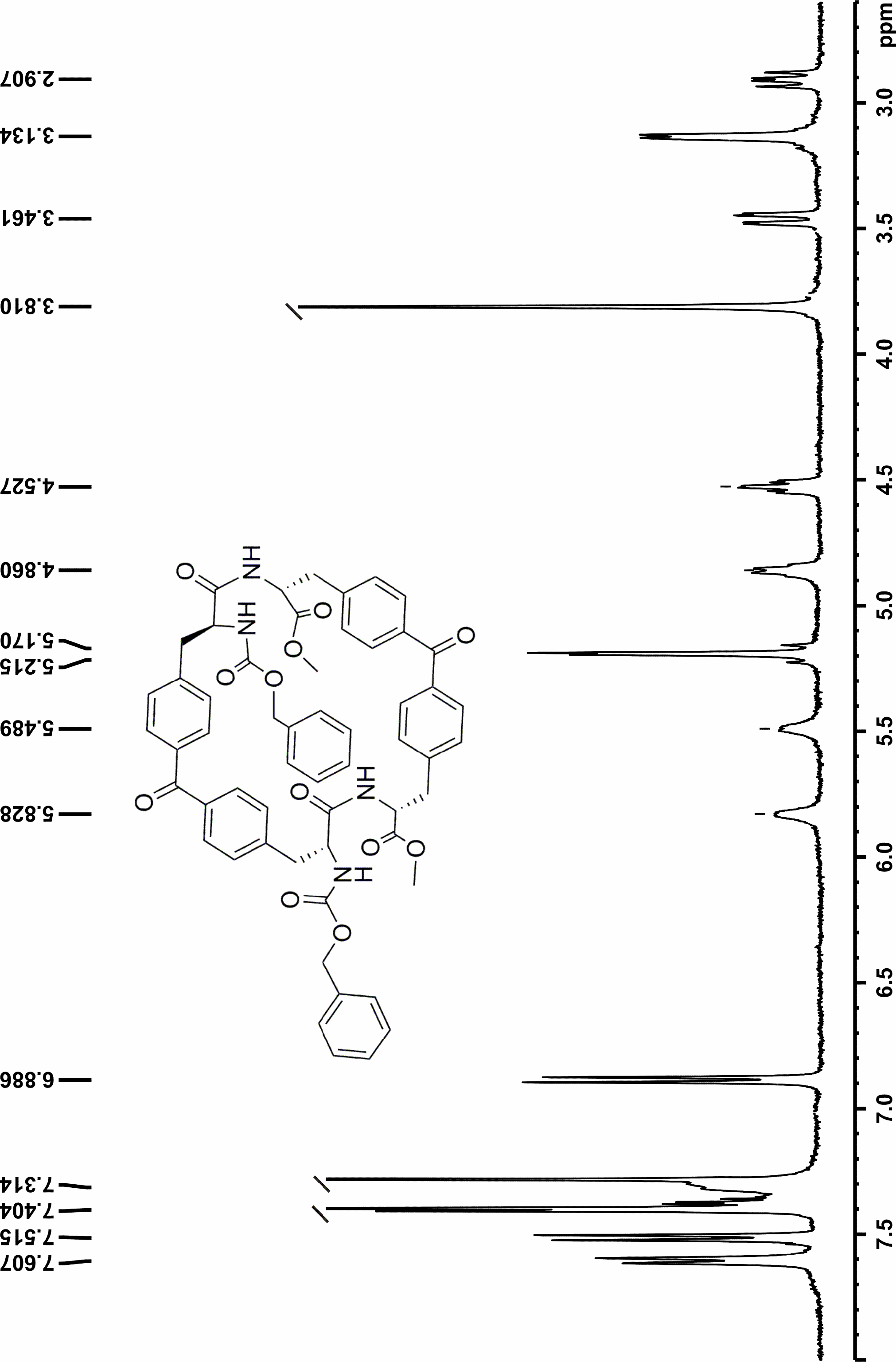


^13^C NMR, 100 MHz, CDCl_3_


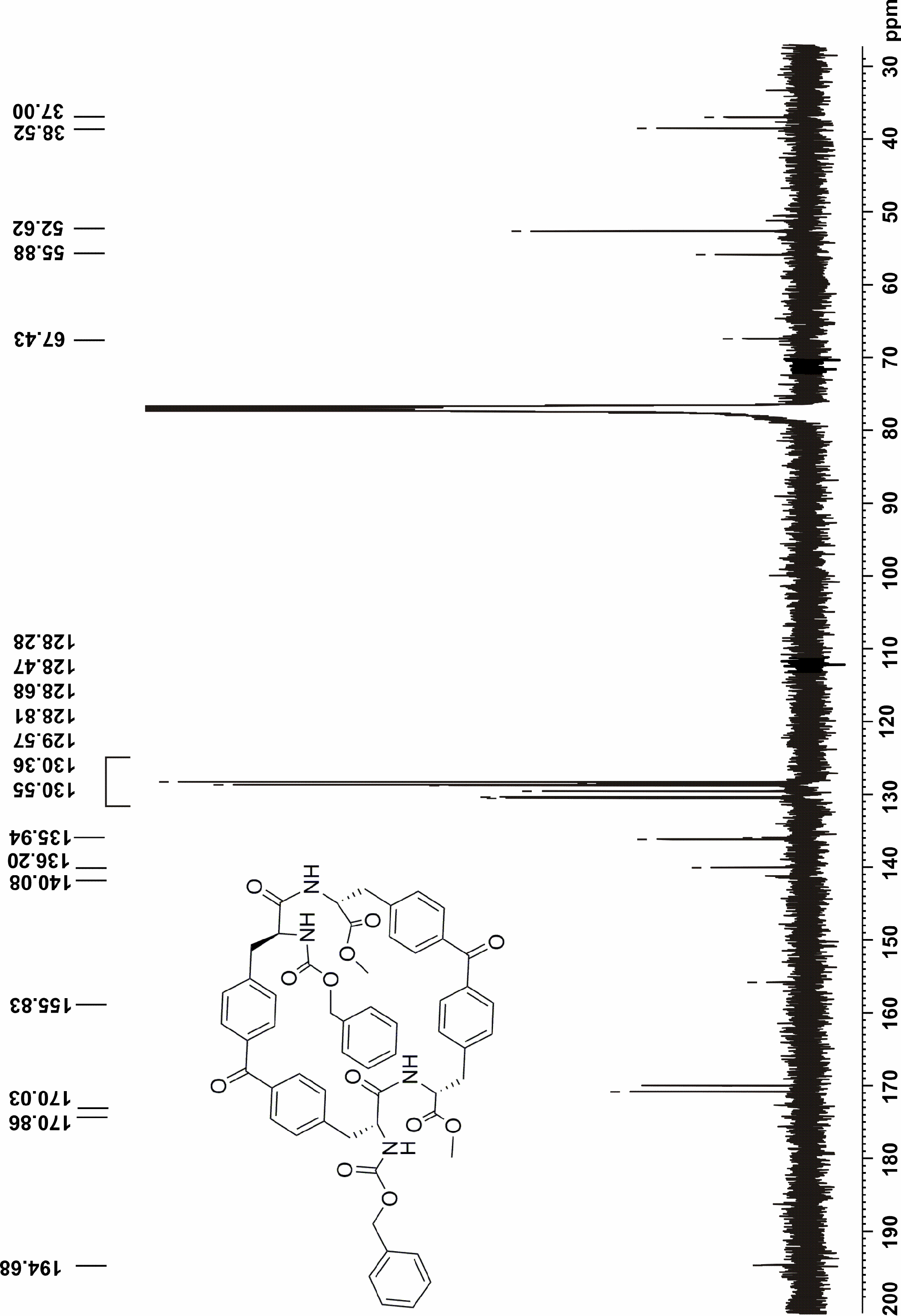

Supplement: File 1 — Synthesis and binding studies of two new macrocyclic receptors for the stereoselective recognition of dipeptides [file Beilstein_J_Org_Chem-06-05-s001.doc]
